# SATB1 is a targetable modulator of JAK-STAT signaling and cytokines in human Treg and Tconv cells

**DOI:** 10.1038/s44319-026-00812-6

**Published:** 2026-06-12

**Authors:** Saskia Kolb, Leonie Diekmann, Elizabeth E Lochert, Linda Warmuth, Julia Ritter, Gunter Schmidtke, Michael Weber, Markus Hoffmann, Markus List, Daniel Kotlarz, Isabelle Serr, Carolin Daniel, Dirk H Busch, Christian Schmidl, Kathrin Schumann

**Affiliations:** 1 Technical University of Munich (TUM), School of Medicine and Health, Department of Preclinical Medicine, Institute for Medical Microbiology, Immunology and Hygiene, Munich, Germany; 2 Department of Biology, Immunology, University of Konstanz, Konstanz, Germany; 3 Technical University of Munich (TUM), School of Life Sciences, Department of Data Science of Systems Biology, Freising, Germany; 4 TUM, German Heart Center. Department of Anaesthiology, Munich, Germany; 5 National Institute of Diabetes, Digestive, and Kidney Diseases, Bethesda, MD USA; 6 Department of Biochemistry and Molecular & Cellular Biology, Georgetown University Medical Center, Washington, D.C., USA; 7 Technical University of Munich, Munich Data Science Institute (MDSI), Garching, Germany; 8 Ludwig Maximilians University (LMU), Department of Pediatrics, Dr von Hauner Children’s Hospital, University Hospital, Munich, Germany; 9 Institute of Translational Genomics, Helmholtz Zentrum München, German Research Center for Environmental Health, Neuherberg, Germany; 10 German Center for Child and Adolescent Health (DZKJ), partner site Munich, Munich, Germany; 11 Institute for Metabolism and Immunology, Helmholtz Diabetes Center at Helmholtz Munich, Munich, Germany; 12 German Center for Diabetes Research (DZD), Munich, Germany; 13 Institute of Clinical Pharmacology, LMU University Hospital, LMU Munich, Munich, Germany; 14 German Center for Infection Research, Deutsches Zentrum für Infektionsforschung (DZIF), Partner Site Munich, Munich, Germany; 15 Epigenetic Immuno-oncology, Leibniz Institute for Immunotherapy, Regensburg, Germany

**Keywords:** Cancer, Immunology, Signal Transduction

## Abstract

The chromatin organizer SATB1 is indispensable for thymic regulatory T cell (Treg cell) development and T helper cell induction. Several gene loci have been described to be SATB1-controlled, including the transcription factor GATA3 and the cytokine loci IL-4 and IL-17. However, the global effects of SATB1 on fully differentiated human CD4 conventional T cells (Tconv cells) and Treg cells, and thus the potential of SATB1 as a target for T-cell engineering, are poorly understood. Here, we describe SATB1-regulated gene signatures as largely subset-specific, with broader effects on Treg cells. Despite distinct gene-regulatory patterns, we observe overarching dysregulated cytokine and JAK-STAT signaling after *SATB1* ablation. Functionally, *SATB1* KO reduces suppressive capacities of human Treg cells but boosts tumor clearance via CD4 CAR T cells in a preclinical, humanized mouse model. Taken together, Treg destabilization and simultaneous increased activation of CD4 CAR T cells by SATB1 modulation may be a strategy to boost the efficiency of CAR T cell therapies.

## Synopsis

The chromatin organizer SATB1 regulates large gene networks in Treg and Tconv cells. SATB1 ablation disrupts cytokine and JAK-STAT signaling, weakens Treg suppressive capacity, and enhances CD4 CAR T cell-mediated tumor clearance.

- SATB1 controls largely subset-specific gene networks, with broader effects in Treg cells.
- Loss of SATB1 disrupts cytokine-cytokine receptor and JAK-STAT signaling pathways.
- SATB1 knockout reduces Treg suppressive capacity but enhances tumor clearance mediated by CD4 CAR T cells.

## Abstract (web summary)

The chromatin organizer SATB1 regulates large gene networks in Treg and Tconv cells. SATB1 ablation disrupts cytokine and JAK-STAT signaling, weakens Treg suppressive capacity, and enhances CD4 CAR T cell-mediated tumor clearance.

## Graphical abstract

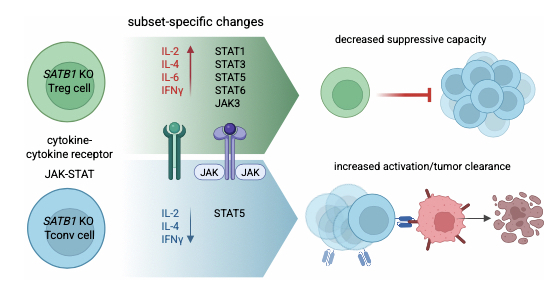

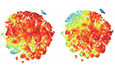

## Introduction

The impact of Satb1 (SATB Homeobox 1), a transcription factor and chromatin organizer, on T-cell function was first described in knockout (KO) mouse models. *Satb1* KO mice have a reduced overall survival as well as small thymi and spleens as a result of arrested T-cell development (Alvarez et al, 2000). During thymic development, Satb1 is induced in a TCR-dependent manner and, in turn, regulates the expression of lineage-defining genes such as *Runx3*, *Cd8*, *Cd4*, as well as the Treg master transcription factor *Foxp3* (Kakugawa et al, 2017; Kitagawa et al, 2017). Mice with conditional *SATB1* KO in CD4 T cells have lower Foxp3+ Treg cell numbers and reduced DNA hypomethylation at *Foxp3* conserved non-coding region 2 (CNS2), indicating cellular destabilization (Kitagawa et al, 2017). Also, in fully differentiated human Treg cells, SATB1 is a positive regulator of FOXP3 expression (Schumann et al, 2020). However, Foxp3 is also in turn regulating SATB1 expression (Beyer et al, 2011).

Also, Tconv cells depend on Satb1 in the steady state for proper cell function. *Satb1* KO Tconv cells have been described to be more susceptible to Treg cell-mediated suppression due to lower expression levels of CD25 and IL-2 (Gupta et al, 2022). Satb1 is further known as a regulator of different cytokine loci in Th2 cell differentiation in the periphery and a regulator of Th17 differentiation, effector tissue phenotype in experimental autoimmune encephalomyelitis (Ahlfors et al, 2010; Köhne et al, 2025; Yasuda et al, 2019). Transcription factors are increasingly the focus for novel chimeric antigen receptor (CAR) T cell engineering approaches to stabilize or induce certain effector phenotypes (Dai et al, 2024; Doan et al, 2024). However, SATB1-controlled gene signatures have not been dissected within this context, and their functional consequences are unclear. In general, our knowledge of Satb1/SATB1 function in fully differentiated human CD4 T-cell subsets is still incomplete, particularly during inflammation.

In this study, we analyzed the impact of SATB1 ablation in fully differentiated CD4 T cells on chromatin and mRNA levels and the resulting changes for key signaling pathways and effector functions in a cell-type-specific manner. Analyzing SATB1’s gene and chromatin modulation during pro-inflammatory stimulation reveals largely distinct chromatin and gene signatures in CD4 Tconv and Treg cells, with a greater impact on gene signatures in Treg cells. JAK-STAT and cytokine–cytokine receptor signaling pathways are dysregulated in both cell types in a pro-inflammatory microenvironment after SATB1 ablation. Functional validation of *SATB1* KO cells revealed diminished suppressive capacity in Treg cells and improved effector function in CAR CD4 T cells.

## Results

### SATB1 is a regulator of cytokine and FOXP3 expression in Treg and Tconv cells

**Figure 1:**
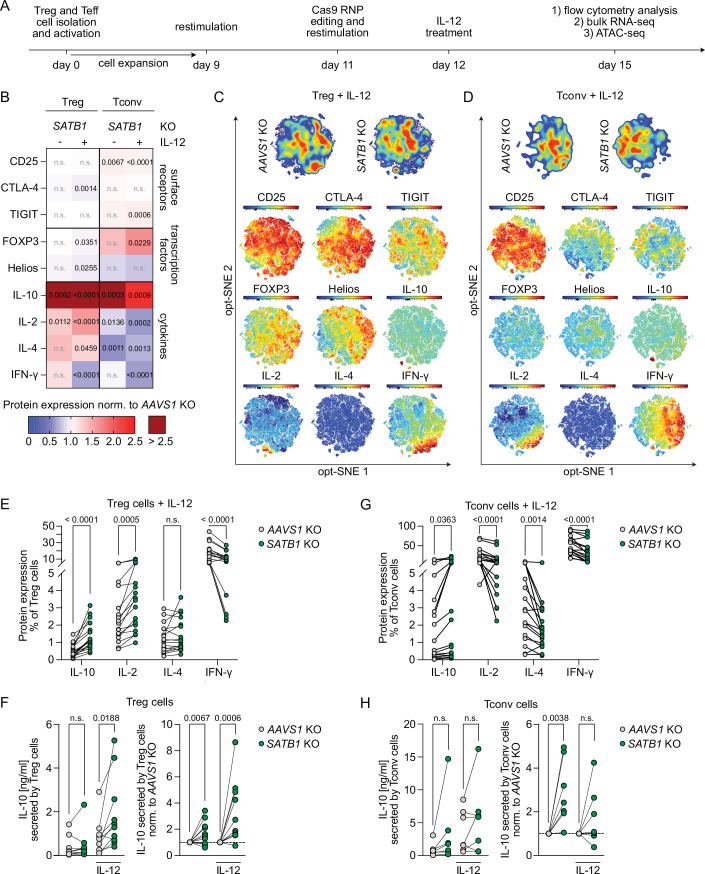
*SATB1* KO causes a destabilized Treg phenotype and enhanced activation in Tconv cells. (**A**) Workflow of in vitro *SATB1* KO experiments in human Treg and Tconv cells. Treg and Tconv cells were isolated from the blood of healthy donors, ex vivo expanded, CRISPR-edited, and challenged with or without IL-12. Cells were cultured with IL-2 throughout the experiment (Treg: 600 IU/ml; Tconv: 200 IU/ml). KO cells were phenotypically characterized by flow cytometry, bulk RNA-seq, and ATAC-seq analysis. (**B**) Heatmap displaying fold changes of pro- and anti-inflammatory flow cytometry markers in *SATB1* KO Treg cells and *SATB1* KO Tconv cells, normalized to their respective *AAVS1* KO controls. The fold change was calculated based on the percentage of the individual marker pregated on living cells. *n* = 12–18 (biological replicates), ratio paired *t* test. (**C**, **D**) opt-SNE contour plot of IL-12-treated *AAVS1* KO and *SATB1* KO Treg (**C**) and Tconv cells (**D**). Expression levels (MFI) of tested flow cytometry markers plotted on opt-SNE plot, *n* = 12–18 (biological replicates). (**E**) Absolute percentages of intracellular cytokine flow cytometry stainings of IL-12-treated *AAVS1* KO and *SATB1* KO Treg cells. *n* = 12–17 (biological replicates), paired *t* test. (**F**) Extracellular IL-10 levels determined by LEGENDplex^TM^ assay of IL-12-treated *AAVS1* KO and *SATB1* KO Treg cells. Absolute values (left), normalized to *AAVS1* KO controls (right), *n* = 10, paired *t* test (left), ratio paired t test (right). (**G**) Absolute percentages of intracellular cytokine flow cytometry stainings of IL-12-treated *AAVS1* KO and *SATB1* KO Tconv cells. *n* = 12–18 (biological replicates), paired *t* test. (**H**) Extracellular IL-10 levels determined by LEGENDplex^TM^ assay of IL-12 or control-treated *AAVS1* KO and *SATB1* KO Tconv. Absolute values (left), normalized to *AAVS1* KO controls (right), *n* = 7 (biological replicates), paired *t* test (left), ratio paired *t* test (right). Source data are available online for this figure.

To dissect the impact of SATB1 gene regulation in human CD4 T cells in the steady state and during inflammation, we ablated either the safe-harbor locus Adeno-associated virus integration site 1 (*AAVS1*) as a negative control, or the transcription factor *SATB1* using Cas9 ribonucleoprotein (Cas9 RNP) nucleofection in vitro with IL-2 expanded Treg and CD4 Tconv cells (Fig. 1A) (Schumann et al, 2020). To mimic an inflammatory microenvironment, as observed in inflammatory bowel disease or also in solid tumors, both KO T-cell subsets were challenged with high doses of pro-inflammatory IL-12 in addition to continuous IL-2 supplementation (Mirlekar and Pylayeva-Gupta, 2021; Verstockt et al, 2023). Currently, several strategies to locally administer IL-12 to tumors to boost the efficiency of adoptive T-cell therapies are being tested in the clinic (Minnar et al, 2023). Interestingly, exposure of Treg cells to high doses of IL-12 can additionally induce a Th1-like, destabilized Treg cell phenotype (Daniel et al, 2015; Dominguez-Villar et al, 2011; Koenecke et al, 2012).

**Figure EV1:**
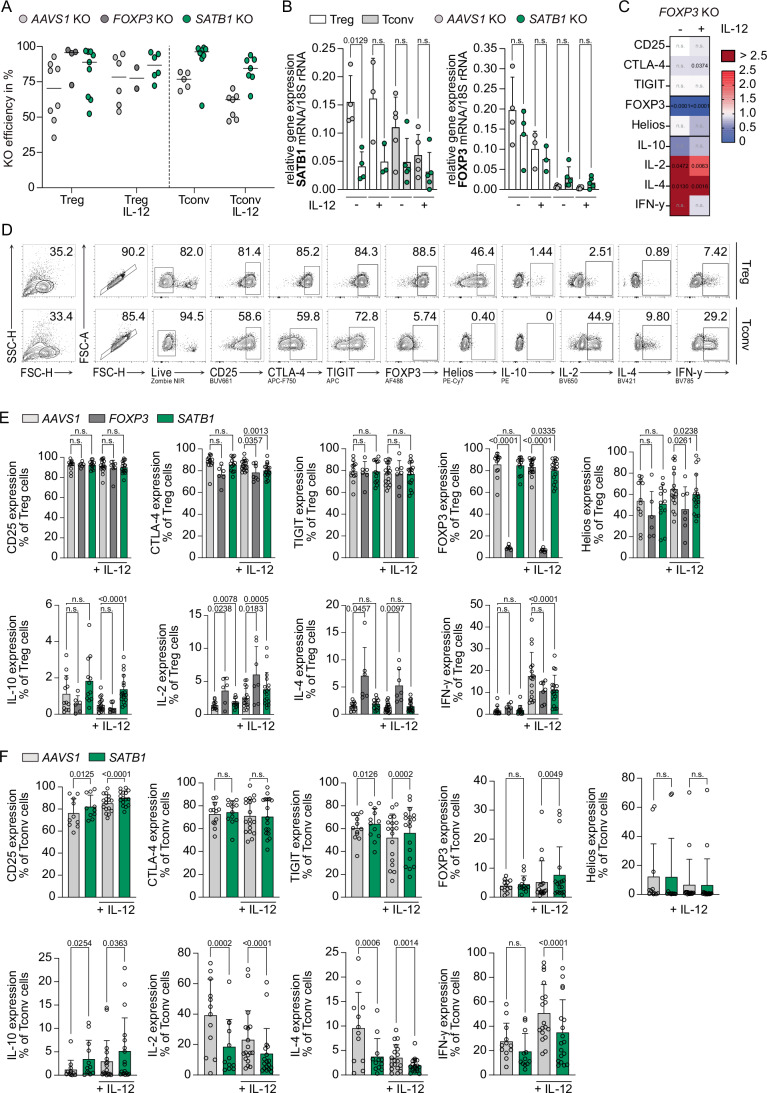
*SATB1* KO validation and overall phenotypic alterations in SATB1-ablated T-cell subsets. Human Treg and Tconv cells were isolated, expanded, activated, and nucleofected with Cas9 RNPs targeting *AAVS1*, *FOXP3*, and *SATB1*. The cells were cultured with or without the pro-inflammatory cytokine IL-12. (**A**) Scatter dot plot displaying KO efficiencies with median. KO efficiencies were determined by amplicon NGS sequencing and TIDE analysis. *n* = 2–9 (biological replicates). (**B**) Bar graphs indicate the mean of relative mRNA expression (ΔCt) of *SATB1* and *FOXP3* in *AAVS*1 and *SATB1* KO Treg cells normalized to 18S rRNA levels. RNA of FACS-sorted living cells was isolated, and qPCR was performed in technical duplicates. *n* = 4–5 (biological replicates), paired *t* test. (**C**) Flow cytometry analysis of canonical pro- and anti-inflammatory markers in *FOXP3* KO Treg cells stimulated with or without IL-12. Percentages of marker-positive cells were normalized to the respective *AAVS1* KO Treg cells with or without IL-12 stimulation. *n* = 6–7 (biological replicates), ratio paired *t* test. (**D**) Flow cytometry gating strategy of *AAVS1* KO control Treg and Tconv cells without IL-12 conditioning. (**E**) Bar graph plots quantifying flow cytometry marker expressions of *AAVS1*, *FOXP3*, and *SATB1* KO Treg cells treated with or without IL-12. Data are also partially shown in Fig. 1B,E. *n* = 6–17 (biological replicates), paired *t* test. (**F**) Bar graph plots quantifying flow cytometry marker expressions of *AAVS1* and *SATB1* KO Tconv cells treated with or without IL-12. Data are also partially shown in Fig. 1B,G. *n* = 12–18 (biological replicates), paired *t* test.

The *AAVS1* and *SATB1* KO rates were comparable in Treg and Tconv cells. IL-12 supplementation reduced the variability in the KO frequencies between donors (Fig. EV1A). Successful *SATB1* KO could also be confirmed on the mRNA level with higher basal SATB1 expression in Treg cells as previously described (Fig. EV1B) (Beyer et al, 2011).

KO cells were phenotypically characterized by flow cytometry after PMA/Iono stimulation (CD25, CTLA-4, TIGIT, FOXP3, Helios, IL-10, IL-2, IL-4, IFNγ). The transcriptional regulator Helios has been associated with Tconv cell activation and Treg cell stability (Allan et al, 2007; Lam et al, 2022). FOXP3 and Helios were both slightly decreased after SATB1 ablation in Treg cells after IL-12 stimulation (Figs. 1B and EV1E; gating strategy: Fig. EV1D). SATB1 has been previously described as a positive regulator of FOXP3 expression (Chaurio et al, 2022; Kitagawa et al, 2017). In our experimental setting, SATB1 ablation in fully differentiated Treg cells resulted in slight reductions of FOXP3 protein as well as mRNA levels (Figs. 1B and EV1B,E). To still exclude a dominant effect of FOXP3 reduction in *SATB1* KO Treg cells, we included *FOXP3* KO Treg cells as an additional control condition to separate FOXP3- from SATB1-dependent effects. These two KO conditions differed in their cytokine profile as well as surface marker expression (Figs. 1B and EV1C,E). Together, these results indicate that in our experimental setting, phenotypic changes in *SATB1* KO Treg cells are largely FOXP3-independent. In *SATB1* KO Tconv, the expression of FOXP3 was heightened, whereas Helios was reduced (Figs. 1B and EV1B,F).

**Figure EV2:**
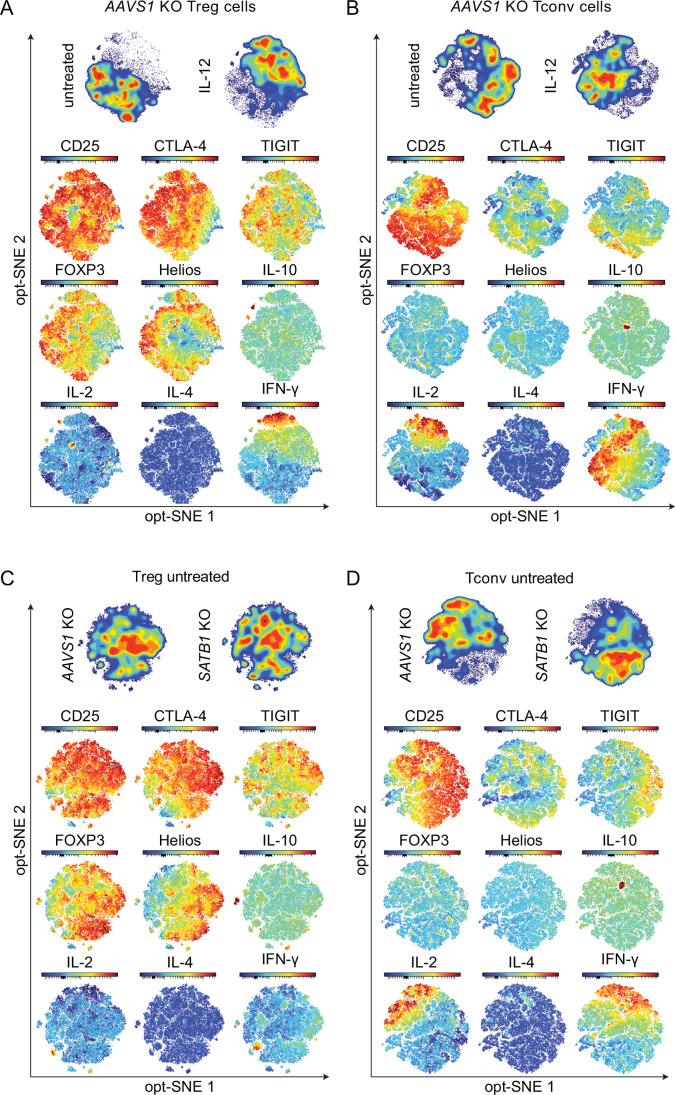
Integrated analysis of protein changes in *AAVS1* and *SATB1* KO Treg and Tconv cells based on flow cytometry. (**A**, **B**) opt-SNE plot of untreated or IL-12-treated *AAVS1* KO Treg (**C**) and Tconv cells (**D**). (**C**, **D**) Expression levels (MFI) of tested flow cytometry markers of untreated *AAVS1* KO and *SATB1* KO Treg (**A**) and Tconv cells (**B**) plotted on opt-SNE density plot. *n*(Treg) = 12–17 (biological replicates), *n*(Tconv) = 12–18 (biological replicates).

Phenotypic changes in KO cell stability were assessed by flow cytometry staining of pro- and anti-inflammatory protein markers (Figs. 1B and EV1E,F; gating strategy: Fig. EV1D). Slight, yet reproducible, changes were observed for the cell activation-dependent surface markers CD25, CTLA-4, and TIGIT. For example, *SATB1* KO Tconv cells upregulated CD25 and TIGIT, especially after IL-12 treatment (Figs. 1B and EV1G). To analyze the impact of these more subtle changes, we plotted the data of IL-12-conditioned cells on opt-SNE density plots. IL-12 treatment resulted in IFNγ-producing cell populations in both cell subsets, with a more pronounced impact on Treg cell clustering driven by IFN-γ induction (Fig. EV2A,B). *SATB1* KO Treg and Tconv cells showed clear shifts compared to their *AAVS1* KO counterparts (Fig. 1C,D). Areas enriched for *SATB1* KO Treg cells were defined by lower levels of FOXP3 or CTLA-4. These areas partially overlapped with reduced Helios expression. However, IL-2-producing SATB1 KO Treg cells clustered separately from these subpopulations (Fig. 1C). *SATB1* KO Tconv cells showed a shift toward higher TIGIT expression and lower IL-2 and IFN-γ (Fig. 1D). Similar patterns could be detected in control-treated cells. However, the differences in control untreated *SATB1* KO Treg cells were less pronounced compared to IL-12 conditioning (Fig. EV2C,D).

Next, we analyzed the cytokine profile of *SATB1* KO Treg and Tconv cells in more depth. *SATB1* KO Treg cells increased their intracellular production of the pro-inflammatory cytokines IL‑2 and IL-4 with or without IL-12 conditioning and IFNγ without IL-12 treatment (Figs. 1B,C,E and EV1E). SATB1 ablation in Tconv reduced pro-inflammatory cytokine production (Figs. 1B,D,G and EV1F). An increase in IL-10 secretion was observed in *SATB*1 KO Treg as well as Tconv cells based on flow cytometry (Figs. 1B–H and EV1E,F). High IL-10 levels have been correlated with heightened Tconv cell activation and Treg cell suppressive capacity (Sun et al, 2009). These changes in IL-10 production were confirmed for *SATB1* KO Treg cells by extracellular detection with LEGENDplex assay and in Tconv without IL-12 conditioning (Fig. 1F,H). In general, taking all the tested markers into account, IL-12 treatment resulted in less phenotypic donor variation and higher statistical significance in most flow cytometry markers tested (Fig. 1B).

Overall, SATB1 ablation in Treg and Tconv cells resulted in subset-specific regulation of pro-inflammatory cytokines.

### SATB1-regulated gene expression and chromatin accessibility are largely distinct in Treg and Tconv cells

Our flow cytometry data showed distinct shifts in pro-inflammatory marker expression in *SATB1* KO Treg and Tconv cells. To test whether these subset-specific gene-regulatory patterns extend to further pathways, *AAVS1* and *SATB1* KO cells were subjected to ATAC-seq and RNA-seq analysis. As we observed more statistically significant changes in protein marker expression after IL-12 stimulation, we focused on KO cells exposed to this pro-inflammatory stimulus (Fig. 1B). Further, we previously identified exposure to a pro-inflammatory environment as an efficient strategy for the dissection of gene networks regulated by individual transcription factors (Schumann et al, 2020).

**Figure 2:**
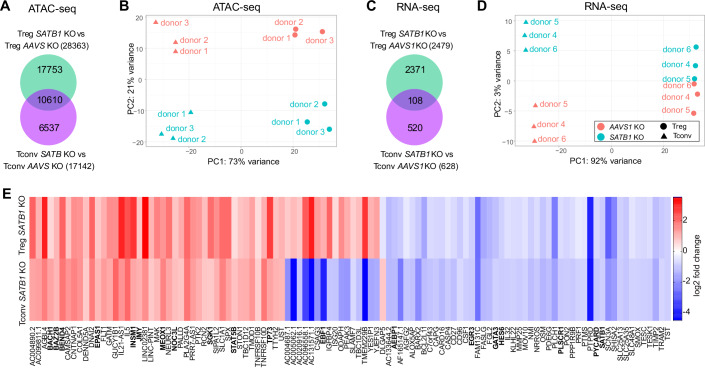
SATB1 controls largely distinct gene signatures in human Treg and Tconv cells after IL-12 treatment. (**A**) Venn diagram of differentially accessible chromatin regions in *SATB1* KO Treg and Tconv cells normalized to *AAVS1* KO control cells (*P* value < 0.05, Wald test using DESeq2). (**A**, **B**) *n* = 3 (biological replicates). (**B**) PCA plot of *AAVS1* and *SATB1* KO Treg and Tconv cells treated with IL-12, analyzed by ATAC-seq. (**C**) Venn diagram of differentially expressed genes in *SATB1* KO Treg and Tconv cells normalized to *AAVS1* KO control cells (*P* value < 0.05, Wald test using DESeq2). (**D**) PCA plot of *AAVS1* and *SATB1* KO Treg and Tconv cells analyzed by RNA-seq after IL-12 conditioning. (**E**) Heatmaps indicating log2 fold change of overlapping RNA-seq data of *SATB1* KO Treg and Tconv cells treated with IL-12. Human transcription factors were indicated in bold. (**C**–**E**) *n* = 3 (biological replicates). Source data are available online for this figure.

**Figure EV3:**
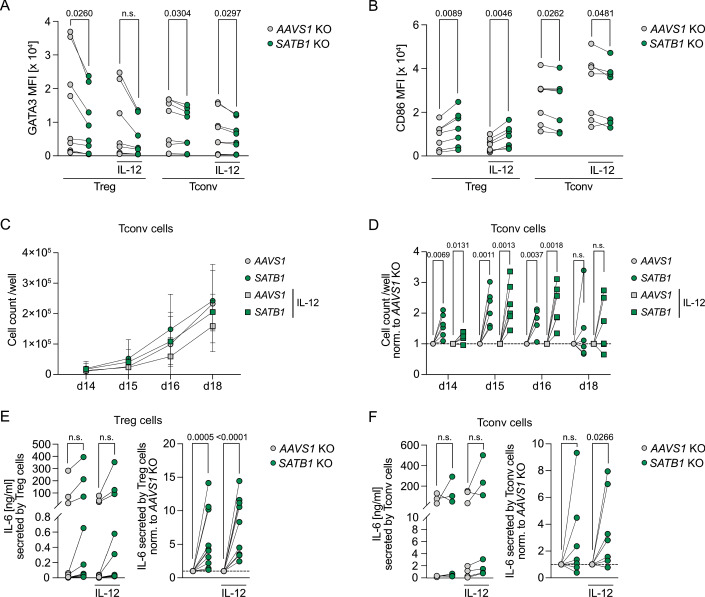
GATA3 and CD86 expression of *SATB1* KO and *AAVS1* control KO Treg and Tconv cells. (**A**) Mean fluorescence intensity (MFI) of GATA3 expression of *SATB1* KO and *AAVS1* control KO Treg and Tconv cells treated with or without IL-12. *n* = 6–9 (biological replicates), paired *t* test. (**B**) Mean fluorescence intensity (MFI) of CD86 and FOXP3 expression of *SATB1* KO and *AAVS1* control KO Treg and Tconv cells treated with or without IL-12. *n* = 8–9 (biological replicates), paired *t* test. (**C**, **D**) *AAVS1* and *SATB1* KO Tconv cell expansion rate at day 14, 15, 16, and 18 of culture with or without the addition of IL-12. (**C**) Absolute cell counts with mean and SD, (**D**) cell counts of *SATB1* KO Tconv cells normalized to respective *AAVS1* KO control cells. *n* = 6 (biological replicates). (**E**, **F**) Extracellular IL-6 levels determined by LEGENDplex^TM^ assay of IL-12-treated *AAVS1* KO and *SATB1* KO Treg (**E**) or Tconv cells (**F**). Absolute values (left), normalized to *AAVS1* KO controls (right), *n* = 7–10 (biological replicates), paired *t* test.

ATAC-seq data revealed a larger number of differently regulated peaks in *SATB1* KO Treg cells (28,363) compared to *SATB1* KO Tconv cells (17,142). 10,610 of these chromatin peaks were regulated in both *SATB1* KO T-cell subsets (Fig. 2A). These widespread changes in chromatin accessibility separated the two T-cell subsets on principal component (PC) 1 in the PCA (PCA plot: 73% variance) but also discriminated *SATB1* KO in comparison to *AAVS1* control cells on PC2 (PCA plot: 21% variance) (Fig. 2B). Analysis of RNA-seq data of *SATB1* KO Treg and Tconv cells revealed 4 times more dysregulated genes in Treg cells (2,479 genes) compared to Tconv cells (628 genes) (Fig. 2C), which resulted in strong separation of the subsets (PCA plot: 92% variance) (Fig. 2D). Only 108 mRNA changes were conserved between both T-cell subsets (Fig. 2C,E). The majority of these mRNA changes were co-regulated, meaning either up- or downregulated in *SATB1* KO Treg and Tconv cells. Several transcription factors are part of these co-regulated genes, including the known SATB1-target GATA3 (Fig. 2E). Reduced GATA3 levels in Tconv cells as well as Treg cells after SATB1 ablation with or without IL-12 treatment could also be confirmed on the protein level via flow cytometry (Fig. EV3A). In murine *Satb1* KO T cells, a shift from Th2 towards Th1 differentiation has been observed, which aligns with our finding in human Tconv cells (Ahlfors et al, 2010; Burute et al, 2012). Overall, these data show largely distinct SATB1-regulated gene signatures in Treg and Tconv cells.

### SATB1 is a transcriptional regulator of JAK-STAT signaling in CD4 T cells

**Figure 3:**
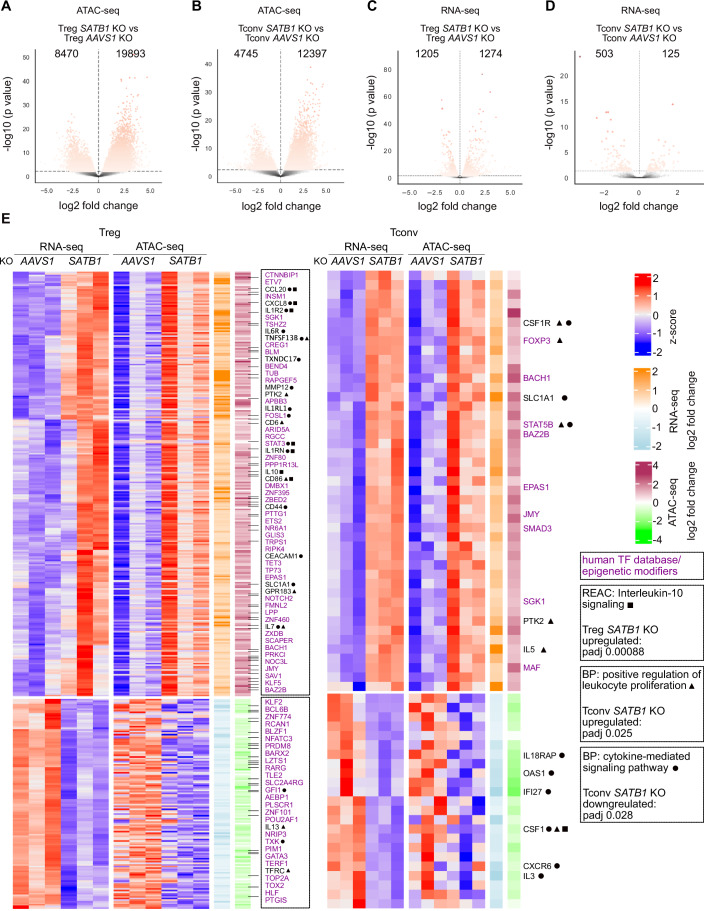
SATB1 differentially controls TF, cytokine expression, and proliferation in human *SATB1* KO Treg and Tconv cells after IL-12 treatment. (**A**, **B**) Volcano plot of differentially accessible chromatin regions of *SATB1* KO Treg versus *AAVS1* KO Treg cells (**A**) and *SATB1* KO Tconv versus *AAVS1* KO Tconv cells (**B**), *n* = 3 (biological replicates). Genes with a *P* value  <  0.05 are highlighted, calculated by a Wald test using DESeq2. (**C**, **D**) Volcano plot of differentially expressed genes of *SATB1* KO Treg versus *AAVS1* KO Treg cells (**C**) and *SATB1* KO Tconv versus *AAVS1* KO Tconv cells (**D**), *n* = 3 (biological replicates). Genes with a *P* value  <  0.05 are highlighted, calculated by a Wald test using DESeq2. (**E**) Heatmaps displaying z-scores of equally regulated chromatin and gene expression changes in *SATB1* KO Treg and Tconv cells treated with IL-12. Differentially regulated TFs are highlighted in purple. Top hits of Reactome (REAC) and biological pathway (BP) analysis and associated adjusted *P* values (*P*adj) if significant are highlighted. *n* = 3 (biological replicates). Source data are available online for this figure.

Next, we examined transcriptional and chromatin changes in Treg and Tconv KO cells individually. In both T-cell subsets, more open chromatin regions could be detected after SATB1 ablation (Fig. 3A,B). However, the RNA-seq data show a more complex pattern: In *SATB1* KO Treg cells, similar numbers of genes were up- or downregulated, whereas in *SATB1* KO Tconv cells, the majority of differentially regulated genes were downregulated (Fig. 3C,D).

**Figure EV4:**
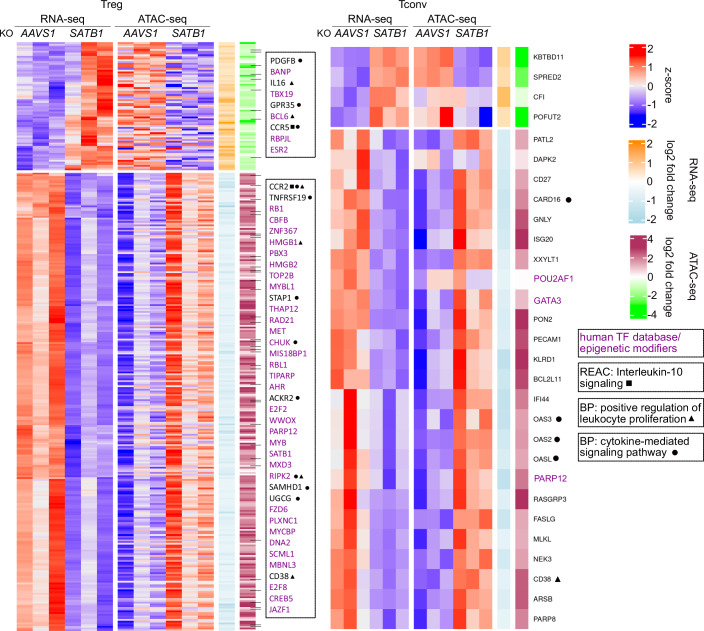
Genes differentially regulated on chromatin and transcription levels in *SATB1* KO Treg and Tconv cells. Heatmaps display z-scores of RNA- and ATAC-seq data of *SATB1* KO and *AAVS1* KO Treg and Tconv cells treated with IL-12. TFs differently regulated in RNA- and ATAC-seq data after *SATB1* KO are highlighted in purple. Genes associated with “Interleukin-10 signaling”, “Positive regulation of leukocyte proliferation”, or “cytokine-mediated signaling” are highlighted. REAC Reactome, BP biological pathway. *n* = 3 (biological replicates).

To integrate RNA-seq and ATAC-seq data, changes in chromatin accessibility in a ± 10 kb window around the transcriptional start site were quantified and integrated with changes in mRNA levels to identify subset-specific signatures (Figs. 3E and EV4) (Thakore et al, 2024). In Fig. 3E, genes are depicted that are regulated in the same manner on the chromatin and mRNA level. Opposite regulatory patterns, which were less frequent, are depicted in Fig. EV4. We identified 267 genes in Treg cells and 45 genes in Tconv cells, which had more open chromatin ±10 kb around the TSS, and correlated with simultaneous mRNA upregulation after SATB1 ablation. Accordingly, 132 genes in *SATB1* KO Treg cells and 23 genes in *SATB1* KO Tconv cells had more closed chromatin and were downregulated (Fig. 3E).

Pathway enrichment analysis of the co-regulated gene sets shown in Fig. 3E elucidated a dysregulation of different cytokine secretion and cytokine signaling in Treg and Tconv cells (full list of pathways: Dataset EV1). Interestingly, in *SATB1* KO Tconv cells, the signature “positive regulation of lymphocyte proliferation” (Biological Pathways (BP)) was enhanced, which is in line with the upregulation of FOXP3, TIGIT, and IL-10 (Fig. 1B,D,G,H). The mentioned BP pathway also affected the oppositely regulated genes in the ATAC-seq and RNA-seq datasets. However, these changes were not significant (Fig. EV4). We compared Tconv cell expansion rates after KO and confirmed stronger cell proliferation in *SATB1* KO Tconv cells compared to *AAVS1* KO cells between day 14 and 16 in culture with or without IL-12 conditioning (Fig. EV3C,D).

**Figure 4:**
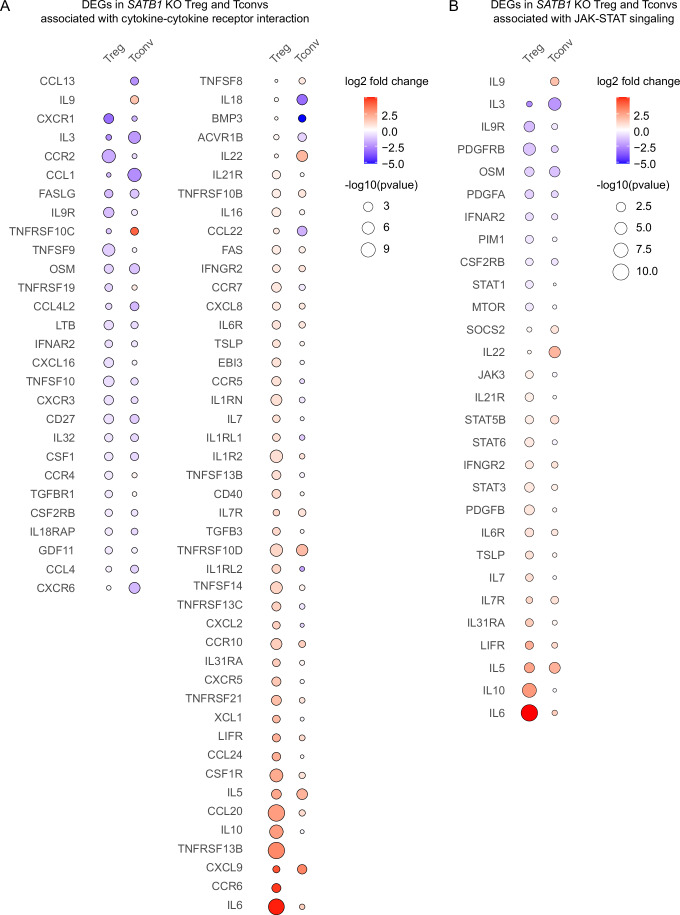
SATB1 differentially regulates cytokine expression and JAK-STAT signaling in human Treg and Tconv cells. Differently expressed genes (DEGs) of the cytokine–cytokine receptor pathway (**A**) and the JAK-STAT signaling pathway (**B**) in *SATB1* KO Treg and Tconv cells treated with IL-12, normalized to the respective IL-12 *AAVS1* KO conditions based on RNA-seq data. Color scheme indicates log2 fold changes of gene expression and the size of the dots the -log10 of the *P* value calculated by a Wald test using DESeq2, *n* = 3 (biological replicates). Source data are available online for this figure.

In *SATB1* KO Treg cells, the IL-10 signaling-related genes, such as *IL10*, *CD86*, and *IL7*, were significantly upregulated (REACTOME database, term “Interleukin 10 signaling”, Dataset EV1). Upregulation of CD86 expression after deletion of SATB1 in Treg cells could be confirmed on the protein level (Fig. EV3B). In *SATB1* KO Tconv cells, genes involved in cytokine-mediated signaling, such as *IL5*, were upregulated, whereas others, including *IL3*, were downregulated (Fig. 3E; BP “cytokine-mediated signaling pathway”, Dataset EV1). Next, we performed KEGG analysis on the global RNA-seq results of *SATB1* KO Treg and Tconv cells to further dissect these patterns. We observed a strong dysregulation of genes related to KEGG “cytokine–cytokine receptor interactions” with more pronounced effects in Treg cells compared to Tconv cells. Again, changes in *SATB1* KO Treg cells were more prominent. Cytokines and cytokine receptors were mostly upregulated (e.g., transcripts of IL-6, IL-10, IL-5, IL7R, IL1LR2, IL6R). The upregulation of IL-6 mRNA in Treg cells was very pronounced after SATB1 ablation (Fig. 4). On protein levels, both *SATB1* KO cell subsets secreted more IL-6. However, the increase was more pronounced in *SATB1* KO Treg cells in both stimulation conditions and more consistent in the different biological replicates (Fig. EV3E,F). Chemokines and chemokine receptors showed a more diverse up- and downregulation. In Tconv cells, overall fewer genes of this pathway were differentially regulated, and their expression was mostly reduced (Fig. 4A).

Besides that, the *SATB1* KO resulted in a massive dysregulation of transcription factors and epigenetic modifiers in Treg cells and, to a lower extent, in Tconv cells (Figs. 2E,  3E, and EV4). STAT3, a transcription factor with known functions in Treg cell stability as well as IL-10 signaling, was upregulated in *SATB1* KO Treg cells (Aqel et al, 2021; Laurence et al, 2012; Poholek et al, 2016). In *SATB1* KO Tconv cells, the STAT family member STAT5b was significantly more accessible in ATAC-seq, which correlated with higher STAT5b mRNA levels (Fig. 3E). KEGG pathway analysis showed significant changes in “JAK-STAT-signaling”, again more pronounced in *SATB1* KO Treg cells compared to Tconv cells. In *SATB1* KO Tconv cell changes in the JAK-STAT pathway could be observed, for example, by increased transcript levels of STAT5B and SOCS2 and minor changes in STAT3 expression levels. In *SATB1* KO Treg cells, transcript levels of JAK3, STAT3, STAT5B, and STAT6 were upregulated, and STAT1 was reduced, among other changes (Fig. 4B).

These results identify SATB1 as a regulator of the JAK-STAT pathway, cytokine expression, and signaling in fully differentiated human CD4 T cells in a subset-specific manner in a pro-inflammatory microenvironment.

### *SATB1* KO impairs Treg cell suppressive functions

**Figure 5:**
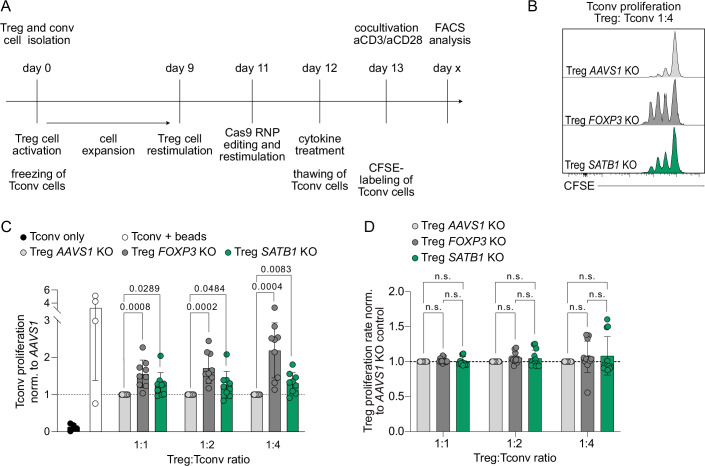
*SATB1* KO impairs Treg suppressive function. (**A**) Workflow of Treg suppression assay. Treg cells were isolated, expanded, and CRISPR-edited. Tconv cells were frozen directly after isolation and thawed on day 12 and rested overnight. Rested Tconv cells were labeled with CFSE and cultivated with KO Treg cells in different Treg:Tconv cell ratios (1:1, 1:2, and 1:4) in the presence of anti-CD3/CD28 stimulus. Final flow cytometry quantification of CFSE-dilution was performed when 40–60% of Tconv cells had undergone at least one cell division. (**B**) Representative histograms of Tconv cell proliferation cultivated with *AAVS1*, *FOXP3*, or *SATB1* KO Treg cells in a Treg: Tconv cell ratio of 1:4. (**C**) Bar plots indicate the mean with SD of Tconv cell proliferation rate normalized to *AAVS1* KO control condition. *n* = 4 (biological replicates), with technical duplicates, two-way ANOVA with Tukey’s multiple comparisons test. (**D**) Bar plots indicating mean with SD of Treg proliferation rate normalized to *AAVS1* KO control condition. *n* = 4 (biological replicates), with technical triplicates, two-way ANOVA. Source data are available online for this figure.

So far, flow cytometry, RNA-seq, and ATAC-seq data analysis depicted a complex pattern of gene dysregulation in *SATB1* KO Treg cells affecting a wide range of cytokine/JAK-STAT proteins with an unclear overall impact on Treg cell functionality. For example, STAT3 has been described as a positive regulator of FOXP3 expression in Treg cells, but also as a driver of Treg cell instability (Laurence et al, 2012; Pallandre et al, 2007). To determine the impact of SATB1-controlled gene signatures on Treg suppressive function, *SATB1* KO Treg cells, as well as *AAVS1* and *FOXP3* KO control Treg cells, were challenged in a T-cell suppression assay (Fig. 5A). As expected, the ablation of FOXP3 had the strongest effect on Treg suppressive capacity. However, in all cell ratios tested, *SATB1* KO Treg cells also had a significantly reduced suppressive capacity compared to AAVS1 control edited cells (Fig. 5B,C). The KO Treg cell numbers were not negatively affected in these assays, excluding a reduced inhibition of Tconv cell proliferation based on diminished Treg survival and/or proliferation (Fig. 5D). Overall, human, ex vivo expanded *SATB1* KO Treg cells are phenotypically and functionally compromised.

### *SATB1* KO in CD4 Tconv cells increases the anti-tumor efficacy of CAR T cell products

Flow cytometry and integration of RNA-seq/ATAC-seq data revealed an enhanced T-cell activation profile, as well as higher STAT5B levels after SATB1 ablation. These changes could hint toward increased cell survival and expansion of Tconv cells after SATB1 ablation, which could potentially be beneficial for CAR T cell therapies.

**Figure 6:**
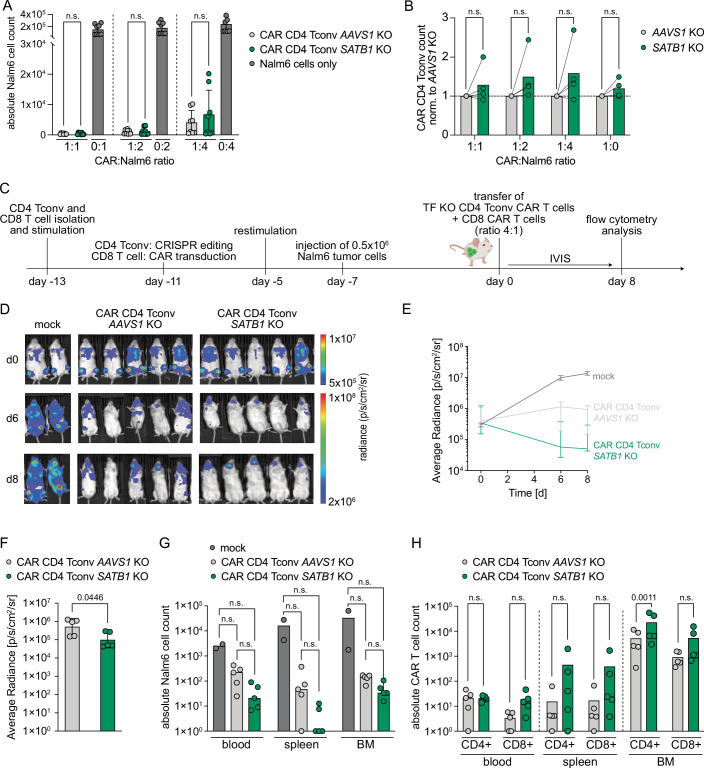
*SATB1* KO enhances CAR Tconv cell-mediated tumor clearance. (**A**, **B**) *AAVS1* and *SATB1* KO Tconv cells were retrovirally transduced with anti-CD19-CAR and co-cultured with or without human CD19+ Nalm6-FFLuc-GFP tumor cells in different CAR T cell to tumor cell ratios (1:1, 1:2, and 1:4). Tumor and CAR T-cell counts were analyzed after 72 h of co-culture. (**A**) Bar plot of absolute counts of Nalm6-FFLuc-GFP tumor cells co-cultured with or without *SATB1* or *AAVS1* KO CAR Tconv cells. *n* = 4 (biological replicates), with technical replicates, bar graphs indicate mean values with SD. Paired *t* test (*AAVS1*, *SATB1*). (**B**) The bar plot indicates fold changes of CD4 CAR T cell expansion. Expansion of *SATB1* KO CAR Tconv cells normalized to *AAVS1* KO control CD4 CAR Tconv cells. *n* = 4 (biological replicates), bar graphs indicate mean values, ratio paired *t* test. (**C**) Schematic workflow of in vivo functional validation of *SATB1* KO CAR CD4 Tconv cells. Nalm6-FFLuc-GFP tumor cells were injected into NSGS mice. After one week, *AAVS1* or *SATB1* KO Tconv cells transduced with anti-CD19-CAR retrovirus were adoptively co-transferred with anti-CD19-CAR-transduced CD8 T cells in a CD4 KO:CD8 cell ratio of 4:1 into Nalm6-FFLuc-GFP tumor-bearing NSGS mice. Tconv and CD8 T cells without CAR transduction served as a mock control. Bioluminescence imaging (IVIS) was performed over time to monitor tumor development. Nalm6-FFLuc-GFP and CAR T cells were quantified in blood, spleen, and bone marrow at day 8. (**D**) Bioluminescence images of Nalm6-FFLuc-GFP-bearing mice treated with mock, *AAVS1* KO, or *SATB1* KO CAR CD4 Tconv cells co-transferred with CAR CD8 T cells. (**E**) Average radiance over time. Tumor burden was quantified as the maximum photon per second per cm^2^ per steradian without the unspecific head signal. Median with SD of average bioluminescence signal on days 0, 6, and 8 is indicated. *n* = 2–5 (biological replicates, mice). (**F**) Bar plot of average radiance. Tumor burden was quantified as the maximum photon per second per cm^2^ per steradian by excluding unspecific head signals. Mean with SD of the average bioluminescence signal on the endpoint is indicated. *n* = 5 (biological replicates, mice), bar graphs indicate mean values, unpaired *t* test. (**G**) Absolute Nalm6-FFLuc-GFP tumor cell count measured via flow cytometry analysis of 100 µl blood, spleen, and bone marrow (BM), log10 scale, n/a values were set to 1, *n* = 2–5 (biological replicates, mice), median is indicated, two-way ANOVA (mixed-effects analysis, Tukey’s multiple comparisons test). (**H**) Bar plots indicating median of absolute CAR T cell count of KO CD4 and CD8 T cells in 100 µl blood, spleen, and bone marrow (BM), log10 scale, n/a values were set to 1, *n* = 5 (biological replicates, mice), two-way ANOVA with Šídák’s multiple comparisons test. (**D**–**H**) T cells of one donor have been transferred. Source data are available online for this figure.

To evaluate the effector functions of *SATB1* KO Tconv cells, we equipped them with anti-CD19 CAR receptors and challenged them in in vitro killing assays. *AAVS1* and *SATB1* KO CD4 CAR Tconv cells were cultivated with CD19-expressing Nalm6-FFLuc-GFP tumor cells in different cell ratios. SATB1 ablation did not negatively affect the killing capacities of these cells (Fig. 6A). Interestingly, we observed an enhanced expansion of *SATB1* KO CAR Tconv cells in most of the biological replicates in this assay, in line with the previously identified gene signature, indicating positive regulation of T-cell expansion (Fig. 6B).

Next, we challenged these cells in vivo. We based this experimental outline on the publication by Ding and colleagues that expressed a constitutively active form of STAT5 specifically in anti-CD19 CAR CD4 Tconv cells. These so-called *CASTAT5* CAR T cells underwent robust expansion and cleared tumors efficiently (Ding et al, 2020). Shortly, *AAVS1* or *SATB1* KO CD4 T cells were transduced with a CD19-targeting CAR and injected together with unedited CD8 CAR T cells into Nalm6-FFLuc-GFP tumor-bearing NSGS mice. SATB1 has been described to prevent premature CD8 T cell exhaustion (Stephen et al, 2017). For that reason, CAR CD8 T cells were applied unedited to avoid biased results in tumor growth. 6 and 8 days after adoptive transfer, CAR T-cell levels and tumor growth were analyzed (Fig. 6C). Interestingly, tumor clearance was more efficient in mice injected with *SATB1* KO CD4 CAR Tconv cells compared to *AAVS1* KO cells (Fig. 6D,E). The average radiance was significantly reduced at the endpoint on day 8 (Fig. 6F). In blood, spleen, and bone marrow, a tendency toward fewer tumor cells could be observed in the *SATB1* KO condition (Fig. 6G). The counts of CD4 and CD8 CAR T cells in blood and spleen were similar in the *AAVS1* and *SATB1* KO conditions. However, in the bone marrow, the preferential site of Nalm6 tumor cell accumulation, an expansion of *SATB1* KO CD4 CAR T cells could be observed. CD8 CAR T cell numbers remained stable in the animals challenged with either *AAVS1* KO or *SATB1* KO CD4 CAR T cells (Fig. 6H). Our results show an improved functionality of CD4 CAR T cells after *SATB1* KO, which affirms the relevance of the CD4 T cell compartment for CAR therapy approaches.

## Discussion

The KO or overexpression of transcription factors for improved CAR T-cell function is currently explored in the context of T-cell exhaustion, T-cell activation, and modulating the plasticity of these cells, which includes overexpression approaches like constitutively activating STAT5 and c-JUN or ablation of NR4A1 and TOX (Dai et al, 2024). Also, in CD4 T cells, especially Treg cells, transcription factors are targets of interest, namely the overexpression of FOXP3 and Helios for enhanced stability and suppressive function (Bittner et al, 2023). Here, we dissected the function of SATB1 in Treg and CD4 CAR T cell function. To be able to apply SATB1 purposefully to genetic engineering approaches, we asked whether SATB1 transcriptional signatures are subset-specific or largely conserved in CD4 T cells. We chose to dissect these networks in an IL-12 microenvironment mimicking inflammation. High doses of IL-12 drive a Th1-like cell phenotype in ex vivo expanded Treg cells defined by an upregulation of IFN-γ and T-bet in mouse and human, while largely maintaining FOXP3 expression (Daniel et al, 2015; Dominguez-Villar et al, 2011; Koenecke et al, 2012; Schumann et al, 2020). These cells have also been associated with positive outcomes in graft-versus-host disease (Koenecke et al, 2012). In this study, we detected cell-specific SATB1 gene-regulatory patterns in both Treg and Tconv cells, with a considerably larger set of affected genes in Treg cells. However, the SATB1-regulated pathways are mostly conserved in these two T-cell subsets. KO of *SATB1* resulted in dysregulated JAK-STAT signaling and changes in cytokine–cytokine receptor interactions, which are closely intertwined.

We observed slightly reduced FOXP3 levels in Treg cells after SATB1 ablation; however, the direct comparison of *SATB1* KO to *FOXP3* KO Treg cells showed a clearly distinct gene-regulatory pattern, which included, for example, increased levels of IL-10. In general, human Treg cells are more prone to downregulate FOXP3 compared to murine Treg cells (Umhoefer et al, 2026). However, SATB1-dependent regulation of murine and human Foxp3/FOXP3 expression is conserved (Beyer et al, 2011). Global analysis by ATAC-seq/RNA-seq highlighted dysregulation of multiple STATs in *SATB1* KO Treg cells. STAT3, STAT5b, and STAT6 transcripts were upregulated, whereas STAT1 transcriptional levels were reduced. All of these proteins have been discussed to different extents in murine Treg cell function. Stat1 and Stat6 signaling have been described as negative regulators of Treg cell induction, stability, and suppressive function, whereas Stat5 is a positive regulator of Foxp3 expression (Arroyo-Olarte et al, 2023; Ma et al, 2011; Yao et al, 2007). Stat3 regulates the balance between Treg and Th17 cells by suppressing Th17 cell differentiation and boosting Treg cell induction (Pallandre et al, 2007; Qin et al, 2024). However, Stat3 inhibition can restore the balance between Treg and Tconv cells during chronic inflammation and can negatively affect Treg stability (Aqel et al, 2021; Laurence et al, 2012).

Overall, changes in individual JAK-STAT signaling pathways potentially contributed to an altered cytokine profile shifted towards pro-inflammatory cytokines such as IL-6.

In contrast to Treg cells, *SATB1* KO Tconv cells displayed reduced levels of pro-inflammatory cytokines. Several publications describe SATB1-mediated regulation of “cytokines-cytokine receptor interactions” in different experimental contexts. Previously, SATB1 has been characterized as a driver of Th2 cell induction (Ahlfors et al, 2010; Cai et al, 2006). In our data, the picture is less clear. *SATB1* KO in Tconv resulted in a downregulation of GATA3 and IL-4, but also in an upregulation of classical Th2 cytokines, including *IL5* and *IL9*, in Tconv cells. However, the in vitro culture conditions applied here—strong stimulation by anti-CD3/CD28 and high doses of IL-2—favor Th0/Th1 cell phenotypes. Gupta et al, described murine *Satb1* KO Tconv cells to be more susceptible to Treg cell suppression in vitro and in vivo due to lower expression levels of CD25 and IL-2, while there was no effect on T-cell proliferation (Gupta et al, 2022). In our experimental setting with human Tconv cells, we also detected decreased IL-2 secretion with only slight effects on IL-2 receptor expression. Nonetheless, integration of RNA-seq and ATAC-seq data of *SATB1* KO Tconv cells highlighted a heightened activation profile. This could be confirmed on a functional level by an increase in vitro and in vivo expansion of CD4 CAR T cells. A heightened activation profile could be (partially) driven by STAT5B. Constitutively active STAT5 has been shown to boost CD4 CAR functionality by increasing cell survival and expansion (Ding et al, 2020). In our experiments, *SATB1* KO CD4 CAR Tconv cells outperformed *AAVS1* KO control cells in a Nalm6 tumor model.

Together, Treg destabilization and simultaneous increased activation of CD4 CAR T cells could be an interesting strategy to boost the efficiency of adoptive CAR T-cell therapies. However, additional work is necessary to fully assess the long-term effects of SATB1 ablation in the human setting and to ensure safety.

### Limitations of our study

Our study strictly focused on the role of SATB1 in human Treg and CD4 Tconv cells, The RNA-seq and ATAC-seq data were acquired after treating the cells with PMA/Iono and IL-12, other conditions were excluded. CAR T cells were stimulated with anti-CD3/CD28 in vitro and in a CAR-specific manner in vivo. Further studies are needed to evaluate the impact of SATB1 on the activity of the JAK-STAT signaling pathways on the protein levels in a global, unbiased, and time-dependent manner, for example, with phosphoproteomics. Nevertheless, in summary, our study shows the distinct impacts of SATB1 transcriptional networks on human Treg and Tconv cell functions.

## Methods

**Table:** Reagents and tools table

Reagent/resource	Reference or source	Identifier or catalog number
**Experimental models**		
Buffy coats/human peripheral blood mononuclear cells (PBMCs)	German Heart Center/Bavarian Red Cross/Donas GmbH	n/a
RD114	in-house	n/a
Nalm6 ffluc-GFP	Stanley Riddell; Fred Hutch Cancer Center	n/a
NSGS mice (NOD.Cg-Prkdcscid Il2rgtm1Wjl Tg(CMV-IL3,CSF2,KITLG)1Eav/MloySzJ)	The Jackson Laboratory	JAX:013062
**Recombinant DNA**		
Cas9 expression plasmid pMJ915	Addgene	plasmid #69090
JCAR021 in pMP72	Juno Therapeutics GmbH, a Bristol-Myers Squibb Company	n/a
**Antibodies**		
Dynabeads Human T-Activator CD3/CD28	Gibco	Cat#: 11132D
Immunocult Human CD3/CD28 T cell activator	Stemcell Technologies	Cat#: 10990
Anti-human CD4-Pacific Blue™ (clone SK3)	BioLegend	Cat#: 344620
Anti-human CD4-PE (clone SK3)	BioLegend	Cat#: 344606
Anti-human CD25-APC (clone BC96)	BioLegend	Cat#: 302610
Anti-human-PE CD127 (clone A019D5)	BioLegend	Cat#: 351304
Anti-human CTLA-4-APC/Fire750 (clone L3D10)	BioLegend	Cat#: 349930
Anti-human IL-2-BV650 (clone MQ1-17H12)	BioLegend	Cat#: 500334
Anti-human IL-10-PE (clone JES-9D7)	BioLegend	Cat#: 501404
Anti-human FOXP3-AF488 (clone 206D)	BioLegend	Cat#: 320112
Anti-human IFNγ-BV785 (clone 4S.B3)	BioLegend	Cat#: 502542
Anti-human Helios-PE-Cy7 (clone 22F6)	BioLegend	Cat#: 137236
Anti-human CD25-BUV661 (clone 2A3)	BD Biosciences	Cat#: 741685
Anti-human IL-4-BV421 (clone MP4-25D2)	BioLegend	Cat#: 500826
Anti-human IL-4-BV785 (clone MP4-25D2)	BioLegend	Cat#: 500846
Anti-human TIGIT-APC (Clone A15153G)	BioLegend	Cat#: 372706
Anti-human EGFR-APC (clone AY13)	BioLegend	Cat#: 352906
Anti-human EGFR-PE (clone AY13)	BioLegend	Cat#: 352904
Anti-human CD19-PE/Dazzle594 (clone HIB19)	BioLegend	Cat#: 302252
Anti-human CD45-PB (clone T29/33)	Dako	Cat#: PB98601-8
Anti-human CD4-PacificOrange (clone RPA-T4)	eBioscience	Cat#: 79-0049-42
Anti-human CD8-APC/Fire™ 750 (clone SK1)	BioLegend	Cat#: 344746
**Oligonucleotides and other sequence-based reagents**		
Alt-R CRISPR-Cas9 tracrRNA	IDT	Cat#. 1072534
Alt-R CRISPR-Cas9 crRNA AAVS1 (5ʹ-GGGACCACCTTATATTCCCA-3ʹ)	IDT, Ursch et al, 2024	n/a
Alt-R CRISPR-Cas9 crRNA FOXP3 (5ʹ-TCATGGCTGGGCTCTCCAGG-3ʹ)	IDT, Schumann et al, 2020	n/a
Alt-R CRISPR-Cas9 crRNA SATB1 (5ʹ-AATGGTCGCACACCACAGTG-3ʹ)	IDT, this study	n/a
AAVS1 fwd primer (5ʹ-AGGGAAGCCTGAGCGCCTCT-3ʹ)	This study	n/a
AAVS1 rv primer (5ʹ-GTCCCGCCTCCCCTTCTTGT-3ʹ)	This study	n/a
AAVS1 sequencing primer (5ʹ-CCACCTCTCCATCCTCTT-3ʹ)	This study	n/a
FOXP3 fwd primer (5ʹ-AGCTCTGCAACTTATTAGCTG-3ʹ)	This study	n/a
FOXP3 rv primer (5ʹ-GCTTAAAGACGGCCATTC-3ʹ)	This study	n/a
FOXP3 sequencing primer (5ʹ-ACTCGATCTCCATGAGCCTCAGT-3ʹ)	This study	n/a
SATB1 fwd primer (5ʹ-CGCATCAGAGCAGGAAGGTAGA-3ʹ)	This study	n/a
SATB1 rv primer (5ʹ-CTGACACTGCCATTCCCTCTCA-3ʹ)	This study	n/a
SATB1 sequencing primer (5ʹ-ACCACAGGCAGCTGATGTATAATTC-3ʹ)	This study	n/a
AAVS1 NSG fwd primer (5ʹ-TCGTCGGCAGCGTCAGATGTGTATAAGAGACAGGGTTCTGGGAGAGGGTAGCGCA-3ʹ)	Ursch et al, 2024	n/a
AAVS1 NSG rv primer (5ʹ-GTCTCGTGGGCTCGGAGATGTGTATAAGAGACAGCCGGGCCCCTATGTCCACTTCA-3ʹ)	Ursch et al, 2024	n/a
FOXP3 NSG fwd primer (5ʹ-TCGTCGGCAGCGTCAGATGTGTATAAGAGACAGCACTCCTCCAGGACAGGCCACA-3ʹ)	This study	n/a
FOXP3 NSG rv primer (5ʹ-GTCTCGTGGGCTCGGAGATGTGTATAAGAGACAGAGACCTGACACCTTTGACCCCCA-3ʹ)	This study	n/a
SATB1 NSG fwd primer (5ʹ-TCGTCGGCAGCGTCAGATGTGTATAAGAGACAGCGCATCAGAGCAGGAAGGTAGA-3ʹ)	This study	n/a
SATB1 NSG rv primer (5ʹ-GTCTCGTGGGCTCGGAGATGTGTATAAGAGACAGTGTGAAAGGGGGCACTCCTTGG-3ʹ)	This study	n/a
18S RNA qPCR primer fw (5ʹ-CTC AAC ACG GGA AAC CTC AC-3ʹ)	This study	n/a
18S RNA qPCR primer rv (5ʹ-CGC TCC ACC AAC TAA GAA CG-3ʹ)	This study	n/a
FOXP3 qPCR primer fw (5ʹ-ACCTACGCCACGCTCATC-3ʹ)	Sturm et al, 2010	n/a
FOXP3 qPCR primer rv (5ʹ-TCATTGAGTGTCCGCTGCT-3ʹ)	Sturm et al, 2010	n/a
SATB1 qPCR primer fw (5ʹ-GTT CTC CCA CAG GGT TCT-3ʹ)	Meng et al, 2012	n/a
SATB1 qPCR primer rv (5ʹ-AAA TGA AGC GTG CTA AAG T-3ʹ)	Meng et al, 2012	n/a
ATAC-seq barcode v2_Ad1.1_TAGATCGC (5ʹ- AATGATACGGCGACCACCGAGATCTACACTAGATCGCTCGTCGGCAGCGTCAGATGTGTAT-3ʹ)	Corces et al, 2017	n/a
ATAC-seq barcode v2_Ad1.2_CTCTCTAT (5ʹ- AATGATACGGCGACCACCGAGATCTACACCTCTCTATTCGTCGGCAGCGTCAGATGTGTAT-3ʹ)	Corces et al, 2017	n/a
ATAC-seq barcode v2_Ad1.3_TATCCTCT (5ʹ- AATGATACGGCGACCACCGAGATCTACACTATCCTCTTCGTCGGCAGCGTCAGATGTGTAT-3ʹ)	Corces et al, 2017	n/a
ATAC-seq barcode v2_Ad1.4_AGAGTAGA (5ʹ- AATGATACGGCGACCACCGAGATCTACACAGAGTAGATCGTCGGCAGCGTCAGATGTGTAT-3ʹ)	Corces et al, 2017	n/a
ATAC-seq barcode v2_Ad1.5_GTAAGGAG (5ʹ- AATGATACGGCGACCACCGAGATCTACACGTAAGGAGTCGTCGGCAGCGTCAGATGTGTAT-3ʹ)	Corces et al, 2017	n/a
ATAC-seq barcode v2_Ad2.1_TAAGGCGA (5ʹ- CAAGCAGAAGACGGCATACGAGATTCGCCTTAGTCTCGTGGGCTCGGAGATGTG-3ʹ)	Corces et al, 2017	n/a
ATAC-seq barcode v2_Ad2.2_CGTACTAG (5ʹ- CAAGCAGAAGACGGCATACGAGATCTAGTACGGTCTCGTGGGCTCGGAGATGTG-3ʹ)	Corces et al, 2017	n/a
ATAC-seq barcode v2_Ad2.3_AGGCAGAA (5ʹ- CAAGCAGAAGACGGCATACGAGATTTCTGCCTGTCTCGTGGGCTCGGAGATGTG-3ʹ)	Corces et al, 2017	n/a
ATAC-seq barcode v2_Ad2.4_TCCTGAGC (5ʹ- CAAGCAGAAGACGGCATACGAGATGCTCAGGAGTCTCGTGGGCTCGGAGATGTG-3ʹ)	Corces et al, 2017	n/a
ATAC-seq barcode v2_Ad2.5_GGACTCCT (5ʹ- CAAGCAGAAGACGGCATACGAGATAGGAGTCCGTCTCGTGGGCTCGGAGATGTG-3ʹ)	Corces et al, 2017	n/a
**Chemicals, enzymes, and other reagents**		
RPMI 1640	Thermo Fisher Scientific	Cat#: 31870074
X-VIVO	Lonza	Cat#: 02-060 F
Recombinant *S.* *pyrogenes* Cas9-NLS protein	in-house	n/a
Recombinant human IL-2	Peprotech	Cat#: 200-02
Recombinant human IL-12	Miltenyi	Cat#: 130-096-704
MojoSort™ Human CD4 T cell Isolation Kit	BioLegend	Cat#: 480130
P3 Primary Cell 96-well Nucleofector® Kit	Lonza Bioscience	Cat#: V4SP-3960
FOXP3 Fix/Perm Buffer Set	BioLegend	Cat#: 421403
CFSE	BioLegend	Cat#: 423801
PMA	Sigma-Aldrich	Cat#: P1585-1mg
Ionomycine	Sigma-Aldrich	Cat#: I9657-1MG
GolgiPlug™	BD Bioscience	Cat#: 555029
123count eBeads	Thermo Fisher Scientific	Cat#: 01-1234-42
Streptavidin-eF450	eBioscience^TM^	Cat#: 48-4317-82
Streptavidin-FITC	BioLegend	Cat#: 405201
DNA Quick Extraction solution	Biosearch Technologies	Cat#: 101094
Quick-RNA Microprep Kit	Zymo Research	Cat#: R1051
RetroNectin	Takara	Cat#: T100B
LEGENDplex™ Human IL-6 Capture Bead B3	BioLegend	Cat#: 740940
LEGENDplex™ Human IL-10 Capture Bead B4	BioLegend	Cat#: 740941
Zombie NIR™	BioLegend	Cat#: 423106
Transposase	Illumina	Cat#: 20034197
GoTaq® Long PCR Master Mix	Promega	Cat#: M4021
NEBNext High Fidelity 2x PCR Mastermix	NEB	Cat#: M0541L
**Software**		
Benchling	https://www.benchling.com	n/a
TIDE webtool	https://tide.nki.nl	n/a
CRISPResso (version 2.2.7)	http://crispresso2.pinellolab.org/submission	n/a
FlowJo (version 10.8.0)	https://www.flowjo.com	n/a
CytExpert 2.3	https://www.beckman.de/flow-cytometry/research-flow-cytometers/cytoflex/software	n/a
GraphPad Prism 10 (version 10.3.1)	https://www.graphpad.com	n/a
OMIQ	https://www.omiq.ai	n/a
RStudio	https://posit.co/download/rstudio-desktop	n/a

### Mouse model

NSGS mice (NOD.Cg-Prkdcscid Il2rgtm1Wjl Tg (CMV-IL3,CSF2,KITLG)1Eav/MloySzJ) (female, 6–8 weeks old, 18–22 g) were acquired from The Jackson Laboratory and kept at the mouse facility at the Technical University Munich, Institute for Medical Microbiology, Immunology and Hygiene. The mice were housed in groups under special, pathogen-free conditions at a constant temperature of 20 °C with constant availability of food and water and subjected to a 12:12 day/night cycle. Littermates of the same sex were randomly allocated to the experimental groups. The performed animal experiments were approved by the district government of Upper Bavaria (Department 5—Environment, Health and Consumer Protection ROB-55.2-2532.Vet_02-18-162).

### Primary human T cells

Buffy coats were collected by the Bavarian Red Cross, Donas GmbH, or the German Heart Center Munich, with the approval of the local institutional review board (Ethics Committee TUM School of Medicine, Technical University of Munich, Ethics Committee of the University of Konstanz) and with the informed consent of the patients. Information about the age and gender of donors is not available. The study conforms to the standards of the Declaration of Helsinki. PBMCs were isolated using gradient density centrifugation with Pancoll (PAN-Biotech) and cultured in T cell medium (TCM) as described in detail below.

### Isolation and expansion of primary human T cells

PBMCs were isolated with Pancoll (Density: 1.077 g/ml; PAN-Biotech) and SepMate^TM^ tubes (STEMCELL Technologies) out of buffy coats. CD4 T cells were pre-enriched with MojoSort^TM^ Human CD4 T cell Isolation kit (BioLegend). For Treg and Tconv cell isolation, CD4 cells were flow cytometry-sorted (anti-human CD4-Pacific Blue™ (clone SK3, BioLegend), anti-human CD25-APC (clone BC96, BioLegend), and anti-human-PE CD127 (clone A019D5, BioLegend)) based on CD4 expression and CD25^high^CD127^low^ (Treg cells) or CD25^low^CD127^high^ (Tconv cells) using a FACS Aria III (Software: FACS Diva 8.0; Becton Dickinson) or a MoFlo Astrios EQ cell sorter (Software: Summit 6.3; Beckman Coulter).

Isolated Treg and Tconv cells were stimulated with Dynabeads™ Human T-Activator CD3/CD28 (Gibco, 25 µl per 1 × 10^6^ cells) in a cell:bead ratio of 1:1 and cultivated in complete Roswell Park Memorial Institute medium (T cell medium (TCM), consisting of RPMI 1640 (Thermo Fisher Scientific) supplemented with 5 mmol/l HEPES (PAN-Biotech), 2 mmol/l glutamine (PAN-Biotech), 50 µg/ml penicillin/streptomycin (PAN-Biotech), 5 mmol/l nonessential amino acids (PAN-Biotech), 5 mmol/l sodium pyruvate (PAN-Biotech) and 10% FCS (fetal calf serum, Gibco)) at 37 °C. Treg cells were cultured at 0.25 × 10^6^ cells/ml with 600 U/ml IL-2 (Peprotech) and Tconv cells at 0.5 × 10^6^ cells/ml with 200 U/ml IL-2 (Peprotech). Expanded Treg cells were tested for FOXP3 and CTLA-4 expression levels at day 7 for quality control (> 95% FOXP3 + CTLA-4 + ).

### CRISPR/Cas9 RNP editing

On day 9 post-isolation, 0.7 × 10^6^ Treg and Tconv cells were stimulated with plate-coated anti-human CD3 (BioLegend, clone UCHT1, 10 µg/ml in 150 µl PBS/48-Well) and soluble anti-human CD28 (BioLegend, clone CD28.2, 5 μg/ml) in the presence of 600 or 200 U/ml IL-2 (Peprotech). 48 h after activation, 0.3 × 10^6^ to 1 × 10^6^ cells were nucleofected with 4 µl Cas9 RNPs and 1 µl 100 µM electroporation enhancer (Sigma-Aldrich) in 20 µl buffer P3 with supplement (Lonza). Cas9 RNPs were generated by mixing 100 µM crRNA (IDT, protospacer sequences: Reagents and Tools Table, (Schumann et al, 2020; Ursch et al, 2024)) and 100 µM tracrRNA (IDT) in a 1:1 ratio and incubated for 5 min at 96 °C. After cooling down to room temperature (RT) 40 μM *Streptococcus pyogenes* Cas9-NLS (in-house production; Cas9 expression plasmid pMJ915 (Addgene) (Lingeman et al, 2017)) was slowly added to the 50 μM crRNA:tracrRNA duplexes and incubated for 15 min at RT. Nucleofections were performed with Amaxa 4D-Nucleofector (Lonza) using program EH-115 for Treg cells and DK-100 for Tconv cells. After nucleofection, cells received additional stimulation using 25 µl ImmunoCult^TM^ (STEMCELL Technologies)/1 × 10^6^ cells and 600 or 200 U/ml IL-2. Treg cells were cultivated with TCM, whereas Tconv cells were cultured in X-VIVO medium (X-VIVO 15 Lonza, supplemented with 50 µg/ml penicillin/streptomycin (PAN-Biotech) and 10% FCS (Gibco)). One day after nucleofection, expanded Treg and Tconv cells were split into two separate conditions treated either with 600 or 200 U/ml IL-2, respectively, or with an additional 1 µg/ml hIL-12 (Miltenyi).

### Flow cytometry analysis

Cells were stimulated with 6.25 ng/ml PMA (Sigma-Aldrich), 1 µg/ml Ionomycin (Sigma-Aldrich), and 1:1200 GolgiPlug™ (BD Bioscience) for 5 h in 160 µl TCM. Surface antibody staining was performed in 30 µl PBS with respective amounts of fluorophore-labeled antibodies at 4 °C in the dark. FOXP3 Fix/Perm Buffer Set (BioLegend) was used to fixate cells for 30 min at RT in the dark. Intracellular staining was performed in 30 µl PERM buffer (1:10 diluted with PBS) for 30 min at RT in the dark. Phenotyping of KO Treg and Tconv cells was performed with Zombie NIR™ (BioLegend), anti-human CTLA-4-APC/Fire750 (clone L3D10MQ1, BioLegend), anti-human TIGIT-APC (clone A15153G, BioLegend), anti-human CD25-BUV661 (clone 2A3, BD Biosciences) anti-human IL-2-BV650 (clone 17H12, BioLegend), anti-human IL-10-PE (clone JES-9D7, BioLegend), anti-human IL-4-BV421 (clone MP4-25D2, BioLegend), anti-human FOXP3-AF488 (clone 206D, BioLegend), anti-human IFN-γ-BV785 (clone 4S.B3, BioLegend), and anti-human Helios-PE-Cy7 (clone 22F6, BioLegend). Cell acquisition was conducted on a Cytoflex LX or Cytoflex S instrument (software CytExpert 2.4, Beckman Coulter) or Aurora analyzer (software SpectroFlo®, Cytek). Cells were analyzed with FlowJo (v10.6). Optionally, FCS files were analyzed in Omiq (OMIQ), and the respective compensation matrix of the FlowJo analysis was applied. Cells were pregated for viable singlets, and the datasets were subsetted depending on the respective CD4 T cell subset and cytokine treatment. 4000 viable singlets of each sample were included in the analysis. opt-SNE clustering algorithm embedded in the software was applied for optimized local structure resolution based on all FACS markers (CD25, CTLA-4, TIGIT, FOXP3, Helios, IL-10, IL-2, IL-4, IFN-γ) (Belkina et al, 2019).

### Legendplex assay

To determine the absolute amount of secreted cytokines LEGENDplex^TM^ HU Essential Immune Response Kit (Biolegend) containing capture beads for human IL-4, IL-2, TNF-α, IL-17 A, IL-6, IL-10, IFN-γ, and active TGFβ was applied. Supernatant of the samples and standards was measured in duplicates. 12.5 µl of the prepared standard dilutions or samples and 12.5 µl assay buffer were transferred to a polypropylene v-bottom plate. 12.5 µl of the diluted capture bead mixture were added and incubated overnight at 4 °C in the dark while continuously shaking. After three washing steps, 12.5 µl of the detection antibodies were added and the samples incubated for 1 h in the dark, while shaking at 800 rpm at RT. Without washing 12.5 µl of the Streptavidin-PE solution was added and incubated for another 30 min at RT while shaking at 800 rpm. For acquisition, the plate was washed, and samples were resuspended in 150 µl of 1× wash buffer. Samples were acquired at the Cytoflex LX or Cytoflex S instrument (software CytExpert 2.4, Beckman Coulter) that was previously set up with the Setup Beads 3 (Raw beads) and Setup Beads 2 (PE beads), provided by the manufacturer.

### Quantification of KO efficiencies with Amplicon Sanger sequencing

To isolate the genomic DNA out of the genetically modified cells, 10 µl cell suspension containing approximately 1 × 10^4^ cells was added to 30 µl QuickExtract DNA Extraction Solution (Biosearch Technologies) and incubated for 6 min at 65 °C followed by 2 min at 96 °C. One PCR reaction contained 12.5 µl of GoTaq® Long PCR Master Mix (Promega), 1.25 µl of 10 µM forward and reverse primer (Microsynth), respectively, 2 µl of extracted DNA solution, and 8 µl of H_2_O. The primers were designed to generate an amplicon of approximately 750 bp around the expected gRNA cut site (see Reagents and Tools Table). The thermocycler setup was as follows: initial denaturation at 95 °C, 3 min; 14 cycles of 98 °C 20 s, 65 °C 20 s, 72 °C 60 s with a touchdown of −1 C/4 s and 29 cycles of 98 °C 20 s, 58 °C 20 s, 72 °C 60 s. Sanger sequencing was performed by Microsynth AG, and KO efficiencies were determined using the TIDE webtool (Brinkman et al, 2014).

### Quantification of KO efficiencies with Amplicon NGS

The indel patterns of CRISPR/Cas9-edited human T cells were determined by amplicon PCR followed by NGS. Primers were designed to result in an amplicon length of 350–500 bps using Benchling (see Reagents and Tools Table, (Ursch et al, 2024)). A 25 µl PCR reaction was performed as described above with the respective primers. The PCR products were cleaned up using AMPure beads according to the manufacturer’s recommendations (Beckmann Coulter) and eluted in 52.5 µl 10 mM Tris. For barcoding, 2 µl of the purified DNA were added to 10 µl of 2x GoTaq® Long PCR Master Mix, 2 µl of Nextera XT index 1 (i7) primer, 2 µl of Nextera XT index 2 (i5) primer (Illumina), and 4 µl of H_2_O. The PCR reactions were heated up to 95 °C for 3 min, followed by eight cycles of 95 °C for 30 s, 55 °C for 30 s, and 72 °C for 30 s, and an elongation step for 5 min at 72 °C. The barcoded PCR products were again purified with AMPure beads and eluted in 27.5 µl 10 mM Tris. Cleaned-up PCR products were quantified using the SpectraMax Quant AccuBlue HighRange dsDNA Kit (Molecular Devices) on the SpectraMax 3x instrument (Molecular Devices). Equal amounts of DNA/sample were pooled and sequenced on an Illumina MiSeq instrument (Illumina) with a MiSeq Reagent Nano Kit v2 (500-cycles) (Illumina) according to the manufacturer’s recommendations. NGS sequencing results were analyzed using CRISPResso (version 2.2.7) with the following prompt: CRISPRessoBatch –batch_settings [name.batch] –amplicon_seq [sequence amplicon] -g [sequence gRNA] -n nhej -gn [name gRNA] -w 30 –skip_failed -o [name of output folder].

### RT-qPCR

In total, 0.5–1 × 10^6^ viable KO Treg and Tconv cells treated with IL-12 were flow cytometry-sorted on day 4 post-nucleofection. RNA isolation was performed with the Quick-RNA Microprep Kit (Zymo Research). cDNA was generated out of 500 ng to 1 mg of RNA. RNA was incubated at 70 °C for 5 min with 150 ng random hexamer primers (Promega). Nuclease-free water was added to the RNA-primer mix to a final volume of 18 µl and mixed with the RT reaction mix: 1 µl reverse transcriptase (M-MLV, Promega), 5 µl M-MLV RT buffer (Promega), and 1 µl or 10 mM dNTPs (New England Biolabs). cDNA synthesis was performed with the initial primer extension step at 22 °C for 10 min, followed by the reverse transcriptase reaction step at 50 °C for 50 min, and finalized with the heat inactivation at 70 °C for 15 min. cDNA was diluted 1:10 to quantify FOXP3 and SATB1 expression and 1:10,000 for the 18S rRNA used as a housekeeping gene. 5 µl 2× GoTaq qPCR master mix (Promega) was mixed with 0.5 µl of forward and reverse primer (Microsynth, see Reagents and Tools Table, (Meng et al, 2012; Sturm et al, 2010)) 10 µM primer stock, and 4 µl of the diluted cDNA. The qPCR protocol was as follows: 95 °C for 300 s, 40 cycles of 95 °C for 10 s, and 60 °C for 30 s on the CFX Connect Real-Time PCR Detection System (Biorad). Data analysis of the RT-qPCR was done by the ΔΔC_T_ method. After normalization to a housekeeping gene, the cycle threshold (CT) values were compared with a sample control.

### RNA-seq

In total, 0.5–2 × 10^6^ viable Treg or Tconv cells were flow cytometry-sorted by applying propidium iodide (BioLegend) 1:500 to the cells. RNA was isolated with the Quick-RNA Microprep Kit (Zymo Research). Novogene, UK, performed library preparation, sequencing, and analysis. In brief, library preparation was performed as follows: mRNA was purified from total RNA using poly-T oligo-attached magnetic beads. After fragmentation, the first strand cDNA was synthesized using random hexamer primers, followed by the second-strand cDNA synthesis using dTTP for the non-directional library (Parkhomchuk et al, 2009). The non-directional library was ready after end repair, A-tailing, adapter ligation, size selection, amplification, and purification. Qubit and real-time PCR were used for quality control and quantification, and bioanalyzer measurements were applied for size distribution detection. Quantified libraries were pooled and sequenced using a Novaseq 6000 X Plus pair-end 150 sequencing strategy.

Raw data (raw reads) of fastq format were cleaned by removing reads containing adapters, poly-N, and low-quality reads from the raw data. The alignment to the reference genome was performed with Hisat2 v2.0.5 (Mortazavi et al, 2008). FeatureCounts v1.5.0-p3 (Liao et al, 2014) was used to count the read numbers mapped to each gene. Then, the FPKM of each gene was calculated based on the length of the gene and the read count mapped to this gene. Read counts were normalized and transformed using VST with DESeq2 (v1.44.0) (Love et al, 2014). Two additional targets (“H” and “D”) were incorporated into the batch correction process to enhance the robustness of the dataset. However, their individual results were excluded from the scope of this study. The transformed data were visualized using PCA and plotted with the R package ggplot2 (v3.5.1) (Wickham, 2010).

Differential expression analysis was performed with DESeq2 (v.1.20.0) (Anders and Huber, 2010; Love et al, 2014). The resulting *P* values were adjusted using Benjamini and Hochberg’s approach to control the false discovery rate. Differentially expressed genes (DEGs) were defined as genes with FDR < 0.05 and log2 fold change >0.5. To visualize the DEGs, volcano plots were drawn using the Python package ngs-toolkit (v0.25.1) (https://github.com/afrendeiro/toolkit, André F. Rendeiro).

### ATAC-seq

At day 4 after Cas9 RNP nucleofection, 5.5 × 10^4^ flow cytometry-sorted, live *AAVS1* or *SATB1* KO Treg and Tconv cells treated with IL-12 were washed with 500 µl ice-cold ATAC buffer (10 mM Tris-HCl pH 7.4; 10 mM NaCl in Ambion H_2_O). Cell lysis was performed with 50 µl cold ATAC buffer supplemented with 10% NP40 (Sigma-Aldrich), 10% TWEEN-20 (Sigma-Aldrich), and 1% Digitonin (Promega) for 3 min at 4 °C. In total, 1 ml cold ATAC buffer with 0.1% TWEEN-20 (Sigma-Aldrich) was used to stop the reaction. The cells were resuspended in 50 µl Transposase Mastermix: TD buffer (Illumina), 16.5 µl DPBS, no calcium, no magnesium (Thermo Fisher Scientific), 0.5 µl 1% Digitonin (Promega), 0.5 µl 10% Tween-20 (Sigma-Aldrich), and 100 nM Transposase (Illumina) and incubated for 30 min at 37 °C. The cleanup of the transposed fragments was performed with the Zymo DNA Clean and Concentrator-5 Kit (Zymo Research) according to the manufacturer’s instructions. 23 µl DNA Elution Buffer (Zymo Research) was added to elute the transposed fragments from the columns. To determine the optimal number of PCR cycles, 10% of the purified ATAC-seq DNA sample was subjected to qPCR to avoid over-amplification of libraries.

In all, 5 µl of NEBNext High Fidelity 2× PCR Mastermix (NEB), 1.9 µl nuclease-free water (Invitrogen), 0.5 µl of 25 µM Ad1.1 primer and Ad2.1 primer (Corces et al, 2017), and 0.1 µl of 100× SYBR green (Thermo Fisher Scientific) were mixed with 2 µl of the purified sample. The qPCR protocol was as follows: 5 min at 72 °C followed by 30 s at 98 °C and 25 cycles of 10 s at 98 °C, 30 s at 63 °C, and 1 min at 72 °C. The optimal cycle number for the final enrichment PCR was determined by rounding up the Ct value of each sample individually. For the final enrichment PCR, 20 µl of the purified sample was mixed with 25 µl NEBNext High Fidelity 2× PCR Mastermix (NEB), 2.5 µl of 25 µM Ad1.xx primer, and 2.5 µl of 25 µM Ad2.xx primer using different barcode combinations for each sample (Thermo Fisher Scientific, see Reagents and Tools Table). The PCR program was performed with the individual cycling numbers described above, and adding a final extension step for 1 min at 72 °C. The PCR product was purified with the Zymo DNA Clean and Concentrator-5 Kit after the vendor’s manual using 15 µl DNA Elution Buffer.

The size selection of 150 to 580 bp fragments was performed with AMPure XP Beads (Beckman Coulter). In total, 0.47× AMPure XP Beads were mixed with the purified tagmented sample and incubated for 10 min at RT. The sample was placed on a magnet for 5 min, and the supernatant was transferred into a new collection tube to eliminate smaller fragments. To remove larger fragments, a final concentration of 1.8× vortexed AMPure XP beads were mixed with the supernatant and incubated 10 min at RT. After 5 min incubation time on the magnet, the supernatant was discarded, and the beads coupled to the DNA were washed two times with 180 µl 80% ethanol. The beads were air-dried for 4 min, and the DNA was eluted using 15 µl TRIS pH 8 (5 min, RT). To remove the beads, the sample was placed for 5 min on the magnet, and the supernatant was transferred to a new collection tube.

The DNA concentration was measured with the Qubit dsDNA HS Assay Kit (Invitrogen). Samples were sent for sequencing to Novogene, UK. The raw data from ATAC-seq were analyzed as described before (Delacher et al, 2021).

### Preprocessing and analysis of ATAC-seq data

ATAC-seq reads were trimmed using Skewer (Jiang et al, 2014) and aligned to the hg19 assembly of the human genome using Bowtie2 (Langmead and Salzberg, 2012) with the ‘-very-sensitive’ parameter and a maximum fragment length of 2000 bp. Duplicate and unpaired reads were removed using the sambamba_v0.7. ‘markdup’ command, and reads with mapping quality >30 and alignment to the nuclear genome were kept. All downstream analyses were performed on these filtered reads. For visualization purposes only, coverage files from filtered bam files were produced using deeptools_v3.5.1 (Ramirez et al, 2014) with the parameters ‘--binSize 10 --normalizeUsing RPGC --effectiveGenomeSize 3300000000 --extendReads 175’.

Peak calling for each sample was performed using MACS2 (Zhang et al, 2008) with the parameters ‘--nomodel --extsize 147’. Peaks overlapping blacklisted features as defined by the ENCODE project (The ENCODE Project Consortium 2012) were discarded. For the analysis of sample sets, a consensus region was created by merging the called peaks from all involved samples, and we quantified the accessibility of each area in each sample by counting the number of reads from the filtered bam file that overlapped each region.

DESeq2 was used on the raw count values for each sample and regulatory element to identify differential chromatin accessibility between samples after normalization of a matrix using the Variance Stabilization Transformation (VST) method and considering the donor as a covariate to remove batch effects (Love et al, 2014). Two additional targets (“H” and “D”) were incorporated into the batch correction process to enhance the robustness of the dataset. However, their individual results were excluded from the scope of this study. Significant regions were defined as having an FDR-corrected *P* value below 0.05 and an absolute log2 fold change above 2. Peaks were assigned to their nearest transcription start site using the HOMER promoter annotation (Heinz et al, 2010).

### Integration of the RNA-seq and ATAC-seq data

The gene-to-peak correlation for ATAC-seq and RNA-seq data was done according to Thakore et al (Thakore et al, 2024) for groups Tconv *SATB1* KO vs Tconv *AAVS1* KO and Treg *SATB1* KO vs Treg *AAVS1* KO. For each differentially expressed gene, a window of ±10 kb around the TSS was defined. All differentially accessible ATAC peaks of the respective groups intersecting this gene window by at least one base were assigned to the gene. When multiple peaks overlapped the same gene window, the peaks and their log2 fold changes were aggregated by calculating the mean. RNA counts and ATAC peaks were transformed using VST with DESeq2 (Love et al, 2014). The z-scores of the gene counts and their assigned peaks, along with their respective log2 fold changes, were visualized using the R library ComplexHeatmap (Gu et al, 2016). The source code used for this analysis is found at https://github.com/daisybio/Tconv-treg-satb1-analysis.

### Treg cell suppression assay

Treg cells were isolated, expanded, and edited with Cas9 RNPs as described previously. One day after Treg CRISPR-editing, a mixture of Tconv cells of different donors (standardized “responder T cells”) was thawed and rested overnight. On the following day, Tconv cells were labeled with CFSE as follows: Up to 1 × 10^7^ Tconv cells were washed with PBS and resuspended in 1 ml PBS. CFSE (2.78 µg/µl, BioLegend) was diluted 1:2000 in PBS. 1 ml of diluted CFSE was added to 1 ml cell suspension and incubated for 5 min at RT in the dark. To stop the staining, 2 ml TCM was added and incubated again for 1 min at RT. The cells were washed once. In total, 1 × 10^5^ CFSE-labeled Tconv cells were cultured together with 1 × 10^4^ Dynabeads™ Human T-Activator CD3/CD28 (Gibco) and different amounts of edited Treg cells ranging from 1:1, 1:2, and 1:4 Tconv:Treg cell ratios. The final readout was conducted after 40–60% of CFSE-labeled Tconv cells had divided at least once upon activation (see Fig. EV4).

### Retrovirus production

In total, 1.2 × 10^6^ RD114 cells (human rhabdomyosarcoma cell line, tested negative for mycoplasma) were seeded in 3 ml DMEM (PAN-Biotech) supplemented with 10% FCS (Gibco) and 1% Penicillin-Streptomycin (PAN-Biotech) in six-well culture plates. 18 µg plasmid DNA (JCAR021 in pMP72, a mutant of JCAR017 (clone: FMC63) from Juno Therapeutics—a Bristol-Myers Squibb company) and 15 µl CaCl_2_ solution (3.31 M, Sigma-Aldrich) were mixed with H_2_O in 150 µl. The mixture was added dropwise while vortexing to 150 µl transfection buffer (1.6 g NaCl (Sigma-Aldrich), 74 mg KCl (Sigma-Aldrich), 50 mg H_2_PO_4_ (Sigma-Aldrich), 1 g HEPES (Sigma-Aldrich) in final 100 ml H_2_O pH 6.76). After 20 min at RT, the transfection reagent was added dropwise to RD114 cells. The medium was exchanged after 4 h to TCM. Retrovirus was harvested 48 h and 72 h post-transfection.

### Nalm6-tumor cell killing assay

CD4 Tconv cells were isolated via flow cytometry-sorting and activated with ImmunoCult^TM^ (5 µl/ 1 × 10^6^ cells, STEMCELL Technologies) in the presence of 200 U/ml IL-2. After 48 h, CRISPR-editing was performed as indicated above. After 2 h of resting, the cells were transduced with retrovirus. Non-treated tissue culture plates were coated with 0.06 µg/ml RetroNectin (Takara) in 300 µl PBS in a 24 well-plate. Overall, 900 µl CAR retrovirus supernatant was coated on the RetroNectin-coated wells by centrifugation (2 h at 3000×*g* and 32 °C). In all, 700 µl supernatant was replaced by CD4 Tconv cell suspension (0.5 × 10^6^ cells/well) supplemented with 200 U/ml IL-2 in the same volume. After one week of cultivation, cell counts were adjusted according to the transduction rate. In total, 2 × 10^4^ CAR-positive *AAVS1* or *SATB1* KO CD4 Tconv cells were cultured together with CD19^+^ Nalm6-FFLuc-GFP tumor cells (acute lymphoblastic leukemia (ALL), tested negative for mycoplasma) in different T cell to tumor cell ratios (1:1, 1:2, and 1:4). CAR CD4 T cell expansion and tumor cell numbers were determined 24 h and 72 h after co-culture. For the final flow cytometry-based readout, the following reagents were used: 7 × 10^3^ 123count eBeads™ (Thermo Fisher Scientific), Zombie NIR (BioLegend), anti-human CD4-PE (clone SK3, BioLegend), anti-human EGFR-APC (clone AY13, BioLegend), and Streptavidin-eF480 (eBioscience^TM^).

### Nalm6-FFLuc-GFP tumor model

Seven days prior to T-cell transfer, 6 to 10-week-old NSGS mice were injected with 0.5 × 10^6^ CD19^+^ Nalm6-FFLuc-GFP cells. Tumor growth in mice was quantified by IVIS imaging one day prior to T cell transfer. Mice were injected intraperitoneally with 150 mg/kg XenoLight D-Luciferin Potassium Salt (PerkinElmer) dissolved in PBS. After 5 min, mice were anesthetized with 2.5% isoflurane RAS-4 Rodent Anesthesia system (PerkinElmer) and imaged in the IVIS Lumina Imaging System (PerkinElmer LAS). The analysis was performed by quantification of photons/s/cm^2^/sr with Living Image 4.5 software (PerkinElmer).

KO CD4 Tconv cells, as well as non-edited CD8 + T cells transduced with JCAR021, were adjusted according to their transduction rate to a final cell ratio of 1.2 × 10^6^ transduced TF KO CD4+ Tconv cells and 0.3 × 10^6^ transduced CD8 + T cells. Mock control mice received CD4 Tconv cells and CD8 T cells without CAR. Tumor growth was determined once per week and at the endpoint by IVIS bioluminescence imaging. Eight days after T-cell transfer, mice were sacrificed, and lymphocytes in blood, spleen, and bone marrow were harvested. 100 µl blood was added to 10 ml ACT buffer (0.17 M NH_4_Cl (Sigma-Aldrich), 0.3 M Tris-HCl (Sigma-Aldrich) pH 7.5) and incubated for 10 min at RT. The lysis was stopped by adding 4 ml of cold TCM. The step was repeated with 5 ml ACT buffer for 5 min after pelleting the cells for 5 min at 1500 rpm. Spleens were mashed through a 100-µm cell strainer, and ACT lysis was carried out with 5 ml ATC buffer for 5 min. The bone marrow was isolated from the femur and tibia of both legs, followed by lysis with 3 ml ACT buffer for 3 min. Isolated cells were characterized by flow cytometry: 1 × 10^4^ 123count eBeads™ (Thermo Fisher Scientific) per condition, anti-human CD19-PE/Dazzle594 (clone HIB19, BioLegend), anti-human CD45-PB (clone T29/33, Dako), anti-human CD4-PacificOrange (clone RPA-T4, eBioscience^TM^), anti-human CD8-APC/Fire™ 750 (clone SK1, BioLegend), Streptavidin-FITC (BioLegend), anti-human EGFR-PE (clone AY13, BioLegend), live/dead staining with propidium iodide (BioLegend).

### Statistical analysis

The specific statistical tests used for each experiment are provided in the accompanying figure legends. No statistical methods were used to predetermine sample sizes. Sample sizes applied in this study were similar to those commonly used in the field. Plots were constructed and analyzed with GraphPad Prism 10 (version 10.3.1). During the experiments and evaluation of the results, the researchers were not blinded to the experimental groups. The only data exclusion criteria applied were low cell survival of primary cells or failed Treg cell quality control at day 7.

## Supplementary information

Peer Review File

Dataset EV1

Source data Fig. 1

Source data Fig. 2

Source data Fig. 3

Source data Fig. 4

Source data Fig. 5

Source data Fig. 6

Expanded View Figures

## Data Availability

The datasets produced in this study are available in the following databases: Flow cytometry data: Zenodo 10.5281/zenodo.19346468. ATAC-seq data: European Genome-phenome Archive, EGAD50000001276. RNA-seq data: European Genome-phenome Archive, EGAD50000001277. The source data of this paper are collected in the following database record: biostudies:S-SCDT-10_1038-S44319-026-00812-6.
