# Supplementary figures and images for: SATB1 is a targetable modulator of JAK-STAT signaling and cytokines in human Treg and Tconv cells
Source: EMBO Rep. 2026 Jun 12;27(14):4029–53. doi: 10.1038/s44319-026-00812-6 (PMC13400746; doi:10.1038/s44319-026-00812-6)

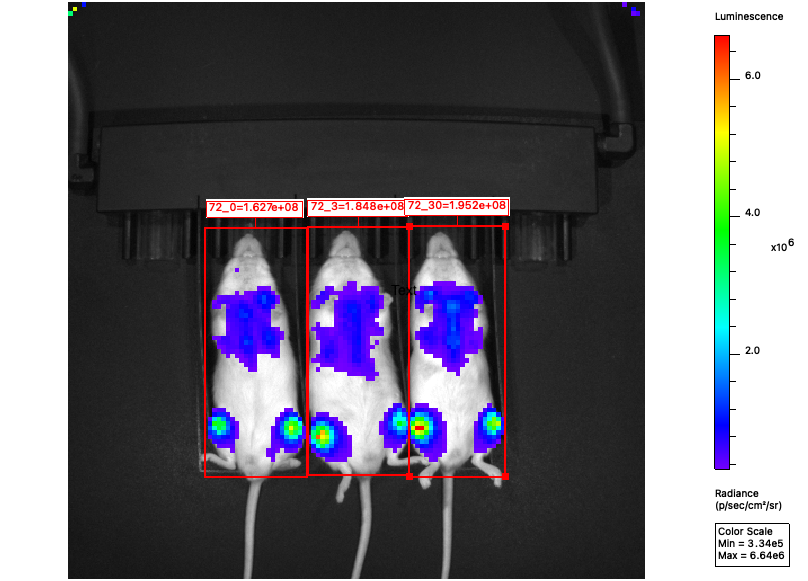

Supplement: Supplementary file 8 — Source data Fig. 6 [file 44319_2026_812_MOESM8_ESM.zip › Figure 6/6D/6D_IVIS_d0/Fig6D_IVIS_d0_72.tif]

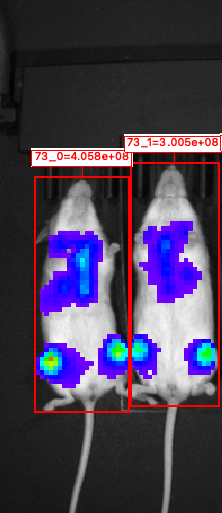

Supplement: Supplementary file 8 — Source data Fig. 6 [file 44319_2026_812_MOESM8_ESM.zip › Figure 6/6D/6D_IVIS_d0/Fig6D_IVIS_d0_73.tif]

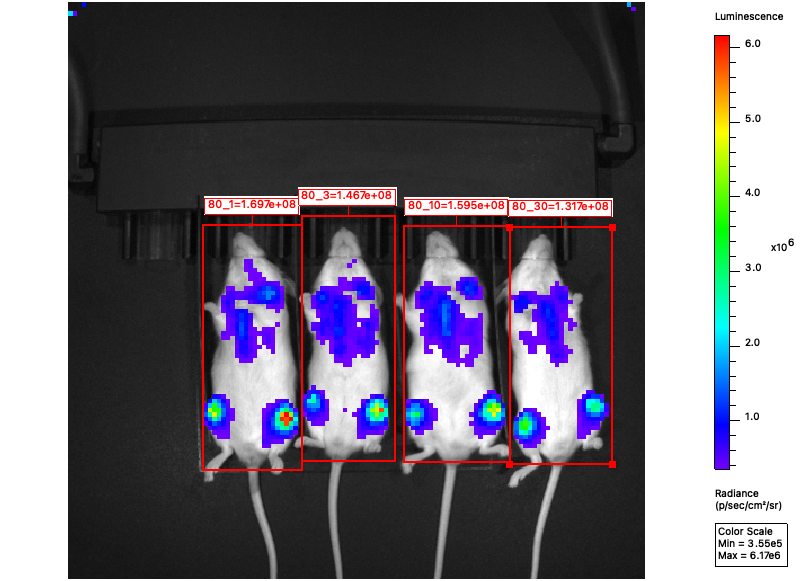

Supplement: Supplementary file 8 — Source data Fig. 6 [file 44319_2026_812_MOESM8_ESM.zip › Figure 6/6D/6D_IVIS_d0/Fig6D_IVIS_d0_80.tif]

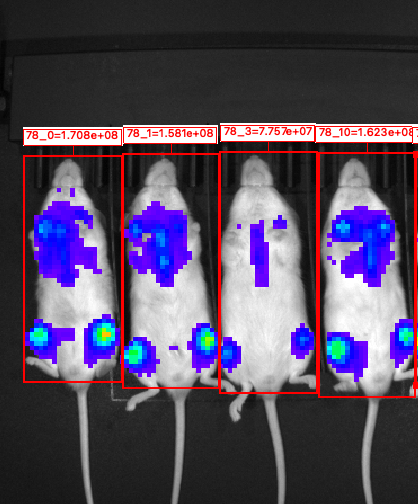

Supplement: Supplementary file 8 — Source data Fig. 6 [file 44319_2026_812_MOESM8_ESM.zip › Figure 6/6D/6D_IVIS_d0/Fig6D_IVIS_d0_78.tif]

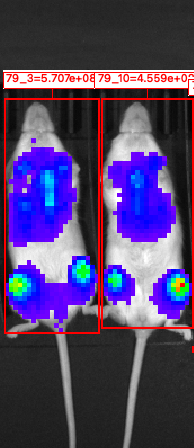

Supplement: Supplementary file 8 — Source data Fig. 6 [file 44319_2026_812_MOESM8_ESM.zip › Figure 6/6D/6D_IVIS_d0/Fig6D_IVIS_d0_79.tif]

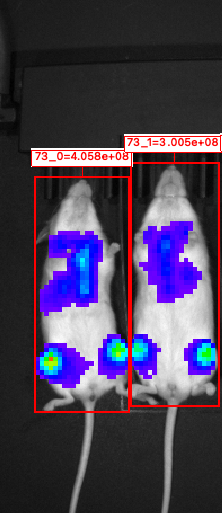

Supplement: Supplementary file 8 — Source data Fig. 6 [file 44319_2026_812_MOESM8_ESM.zip › Figure 6/6D/IVIS_d8/Fig6D_IVIS_d8_73_0-73_1.tif]

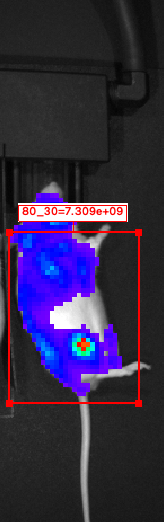

Supplement: Supplementary file 8 — Source data Fig. 6 [file 44319_2026_812_MOESM8_ESM.zip › Figure 6/6D/IVIS_d8/Fig6D_IVIS_d8_80_30.tif]

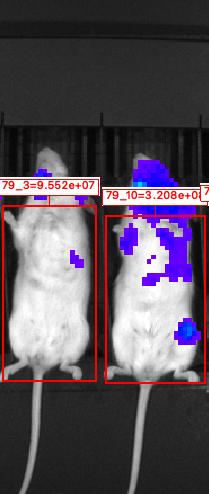

Supplement: Supplementary file 8 — Source data Fig. 6 [file 44319_2026_812_MOESM8_ESM.zip › Figure 6/6D/IVIS_d8/Fig6D_IVIS_d8_79_3-79_10.tif]

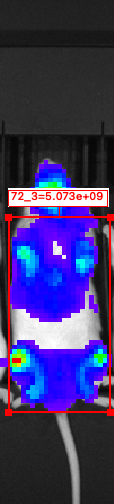

Supplement: Supplementary file 8 — Source data Fig. 6 [file 44319_2026_812_MOESM8_ESM.zip › Figure 6/6D/IVIS_d8/Fig6D_IVIS_d8_72_3.tif]

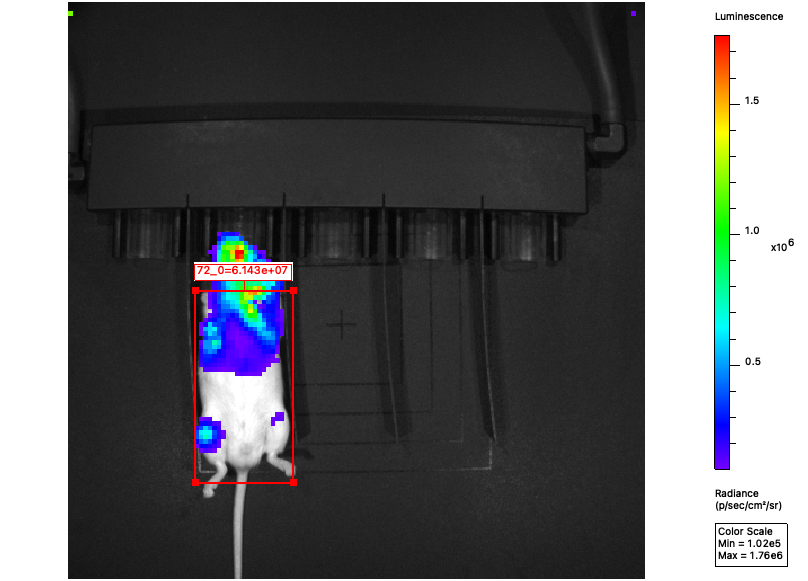

Supplement: Supplementary file 8 — Source data Fig. 6 [file 44319_2026_812_MOESM8_ESM.zip › Figure 6/6D/IVIS_d8/Fig6D_IVIS_d8_72_0.tif]

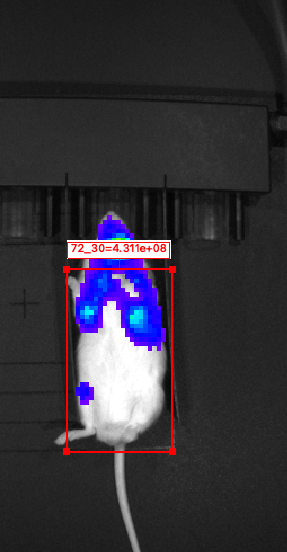

Supplement: Supplementary file 8 — Source data Fig. 6 [file 44319_2026_812_MOESM8_ESM.zip › Figure 6/6D/IVIS_d8/Fig6D_IVIS_d8_72_30.tif]

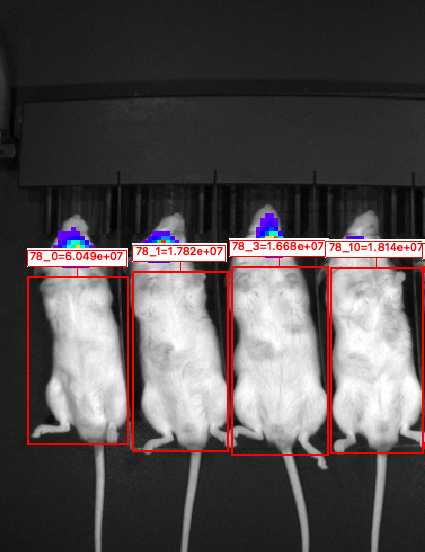

Supplement: Supplementary file 8 — Source data Fig. 6 [file 44319_2026_812_MOESM8_ESM.zip › Figure 6/6D/IVIS_d8/Fig6D_IVIS_d8_78.tif]

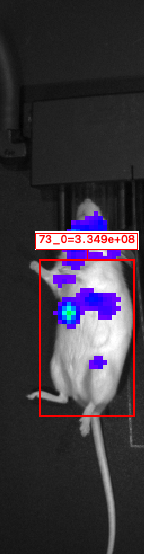

Supplement: Supplementary file 8 — Source data Fig. 6 [file 44319_2026_812_MOESM8_ESM.zip › Figure 6/6D/IVIS_d6/Fig6D_IVIS_d6_73_0.tif]

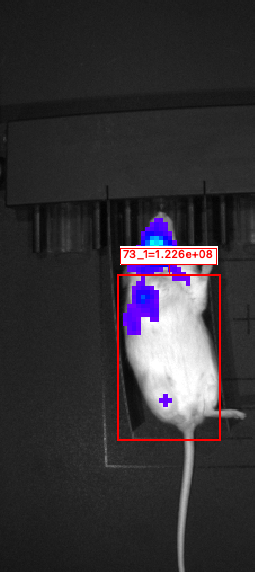

Supplement: Supplementary file 8 — Source data Fig. 6 [file 44319_2026_812_MOESM8_ESM.zip › Figure 6/6D/IVIS_d6/Fig6D_IVIS_d6_73_1.tif]

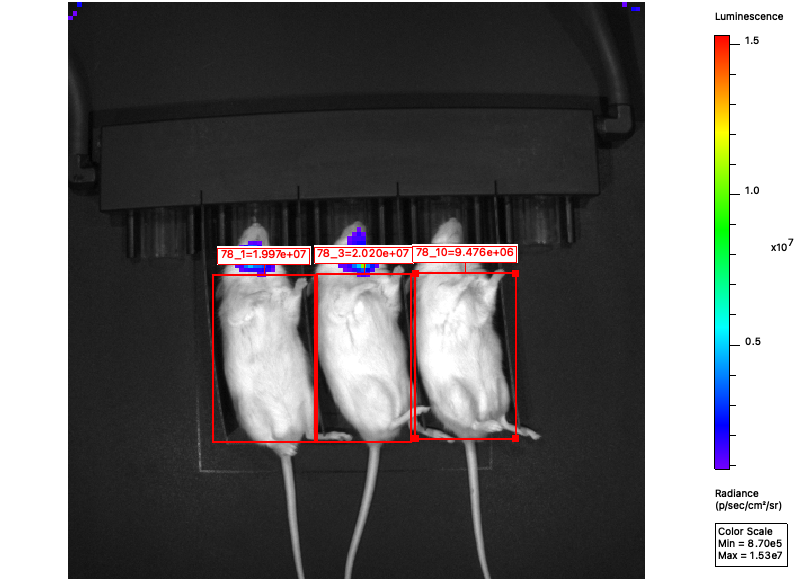

Supplement: Supplementary file 8 — Source data Fig. 6 [file 44319_2026_812_MOESM8_ESM.zip › Figure 6/6D/IVIS_d6/Fig6D_IVIS_d6_78_1-78_3-78_10.tif]

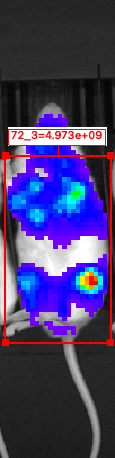

Supplement: Supplementary file 8 — Source data Fig. 6 [file 44319_2026_812_MOESM8_ESM.zip › Figure 6/6D/IVIS_d6/Fig6D_IVIS_d6_72_3.tif]

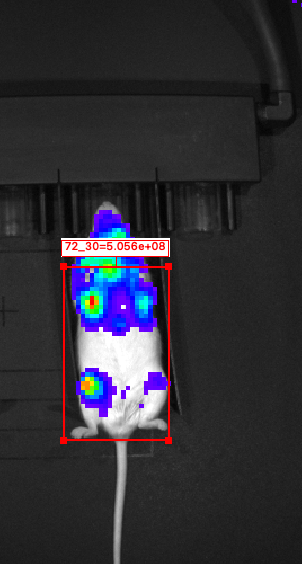

Supplement: Supplementary file 8 — Source data Fig. 6 [file 44319_2026_812_MOESM8_ESM.zip › Figure 6/6D/IVIS_d6/Fig6D_IVIS_d6_72_30.tif]

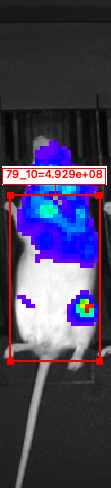

Supplement: Supplementary file 8 — Source data Fig. 6 [file 44319_2026_812_MOESM8_ESM.zip › Figure 6/6D/IVIS_d6/Fig6D_IVIS_d6_79_10.tif]

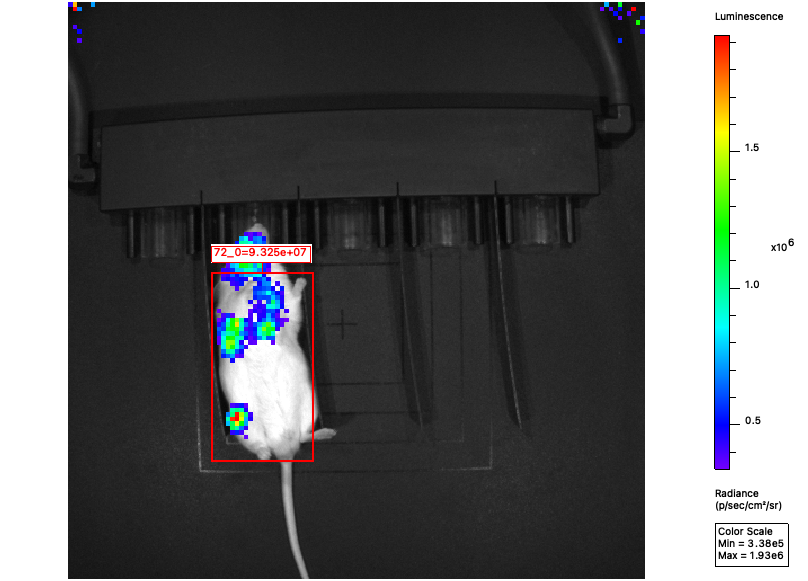

Supplement: Supplementary file 8 — Source data Fig. 6 [file 44319_2026_812_MOESM8_ESM.zip › Figure 6/6D/IVIS_d6/Fig6D_IVIS_d6_72_0.tif]

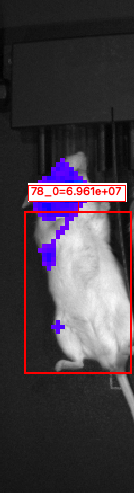

Supplement: Supplementary file 8 — Source data Fig. 6 [file 44319_2026_812_MOESM8_ESM.zip › Figure 6/6D/IVIS_d6/Fig6D_IVIS_d6_78_0.tif]

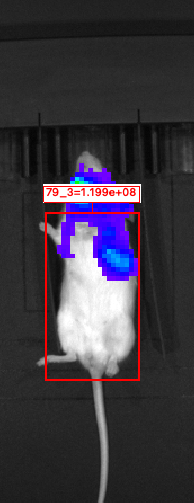

Supplement: Supplementary file 8 — Source data Fig. 6 [file 44319_2026_812_MOESM8_ESM.zip › Figure 6/6D/IVIS_d6/Fig6D_IVIS_d6_79_3.tif]

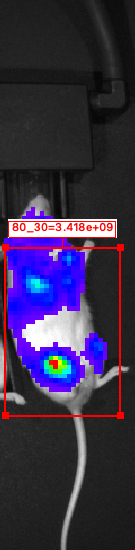

Supplement: Supplementary file 8 — Source data Fig. 6 [file 44319_2026_812_MOESM8_ESM.zip › Figure 6/6D/IVIS_d6/Fig6D_IVIS_d6_80_30.tif]
