# Supplementary material for: SATB1 is a targetable modulator of JAK-STAT signaling and cytokines in human Treg and Tconv cells
Source: EMBO Rep. 2026 Jun 12;27(14):4029–53. doi: 10.1038/s44319-026-00812-6 (PMC13400746; doi:10.1038/s44319-026-00812-6)
Supplement: Supplementary file 9 — Expanded View Figures [file 44319_2026_812_MOESM9_ESM.pdf]

Expanded View Figures

Figure EV1. *SATB1* KO validation and overall phenotypic alterations in SATB1-ablated T-cell subsets.

Human Treg and Tconv cells were isolated, expanded, activated, and nucleofected with Cas9 RNPs targeting *AAVS1*, *FOXP3*, and *SATB1*. The cells were cultured with or without the pro-inflammatory cytokine IL-12. (**A**) Scatter dot plot displaying KO efficiencies with median. KO efficiencies were determined by amplicon NGS sequencing and TIDE analysis. $n = 2$–9 (biological replicates). (**B**) Bar graphs indicate the mean of relative mRNA expression (ΔCt) of *SATB1* and *FOXP3* in *AAVS*1 and *SATB1* KO Treg cells normalized to 18S rRNA levels. RNA of FACS-sorted living cells was isolated, and qPCR was performed in technical duplicates. $n = 4$–5 (biological replicates), paired *t* test. (**C**) Flow cytometry analysis of canonical pro- and anti-inflammatory markers in *FOXP3* KO Treg cells stimulated with or without IL-12. Percentages of marker-positive cells were normalized to the respective *AAVS1* KO Treg cells with or without IL-12 stimulation. $n = 6$–7 (biological replicates), ratio paired *t* test. (**D**) Flow cytometry gating strategy of *AAVS1* KO control Treg and Tconv cells without IL-12 conditioning. (**E**) Bar graph plots quantifying flow cytometry marker expressions of *AAVS1*, *FOXP3*, and *SATB1* KO Treg cells treated with or without IL-12. Data are also partially shown in Fig. 1B,E. $n = 6$–17 (biological replicates), paired *t* test. (**F**) Bar graph plots quantifying flow cytometry marker expressions of *AAVS1* and *SATB1* KO Tconv cells treated with or without IL-12. Data are also partially shown in Fig. 1B,G. $n = 12$–18 (biological replicates), paired *t* test.

 © The Author(s)

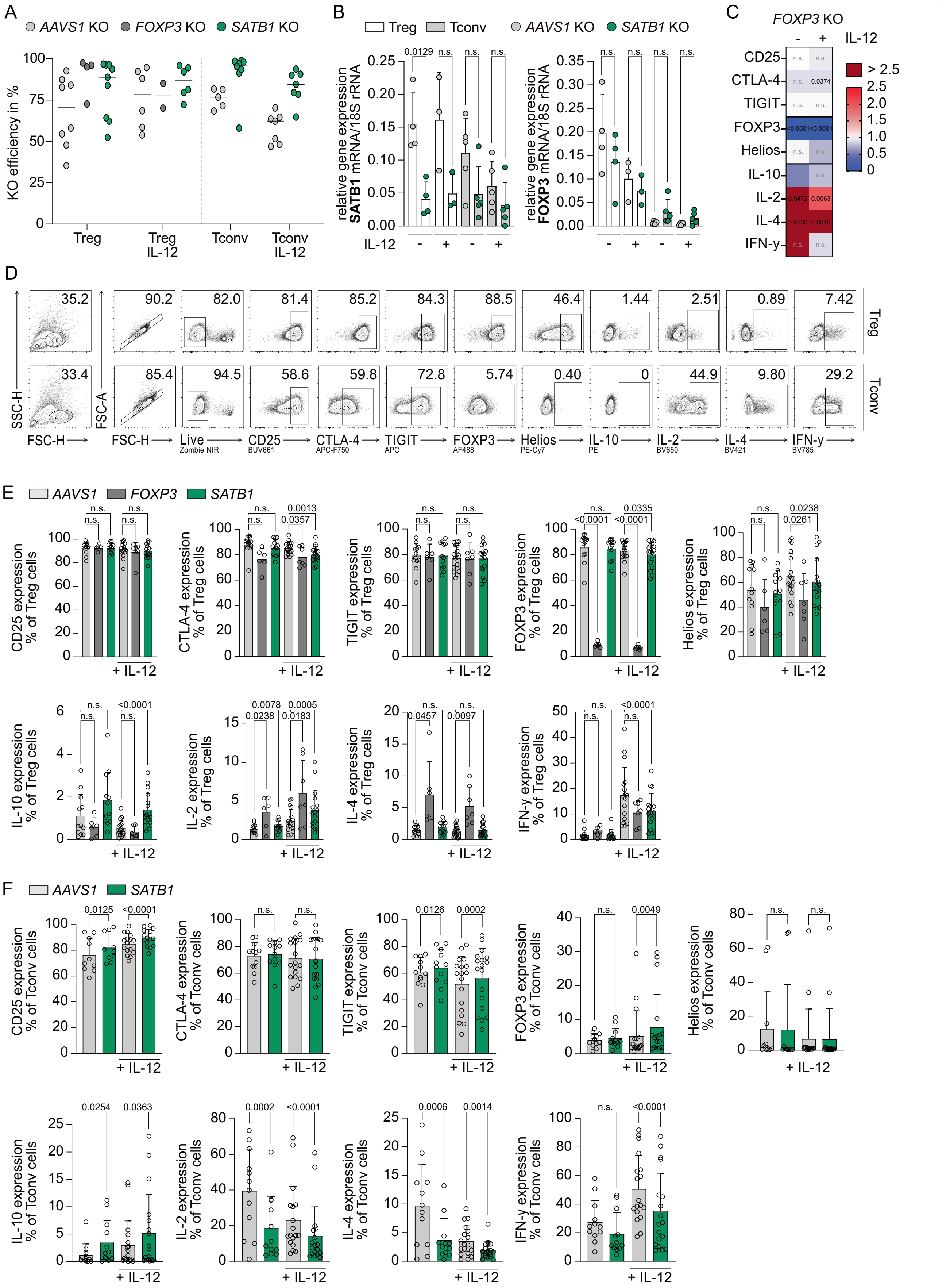
A
AAVS1 KO FOXP3 KO SATB1 KO
KO efficiency in %
Treg
Treg IL-12
Tconv
Tconv IL-12
B
Treg Tconv AAVS1 KO SATB1 KO
relative gene expression SATB1 mRNA/18S rRNA
relative gene expression FOXP3 mRNA/18S rRNA
IL-12
C
FOXP3 KO
IL-12
CD25
CTLA-4
TIGIT
FOXP3
Helios
IL-10
IL-2
IL-4
IFN-y
> 2.5
D
SSC-H
FSC-A
FSC-H
Live Zombie NIR
CD25 BUV661
CTLA-4 APC-F750
TIGIT APC
FOXP3 AF488
Helios PE-Cy7
IL-10 PE
IL-2 BV650
IL-4 BV421
IFN-y BV785
Treg
Tconv
E
AAVS1 FOXP3 SATB1
CD25 expression % of Treg cells
CTLA-4 expression % of Treg cells
TIGIT expression % of Treg cells
FOXP3 expression % of Treg cells
Helios expression % of Treg cells
IL-10 expression % of Treg cells
IL-2 expression % of Treg cells
IL-4 expression % of Treg cells
IFN-y expression % of Treg cells
+ IL-12
F
AAVS1 SATB1
CD25 expression % of Tconv cells
CTLA-4 expression % of Tconv cells
TIGIT expression % of Tconv cells
FOXP3 expression % of Tconv cells
Helios expression % of Tconv cells
IL-10 expression % of Tconv cells
IL-2 expression % of Tconv cells
IL-4 expression % of Tconv cells
IFN-y expression % of Tconv cells
+ IL-12

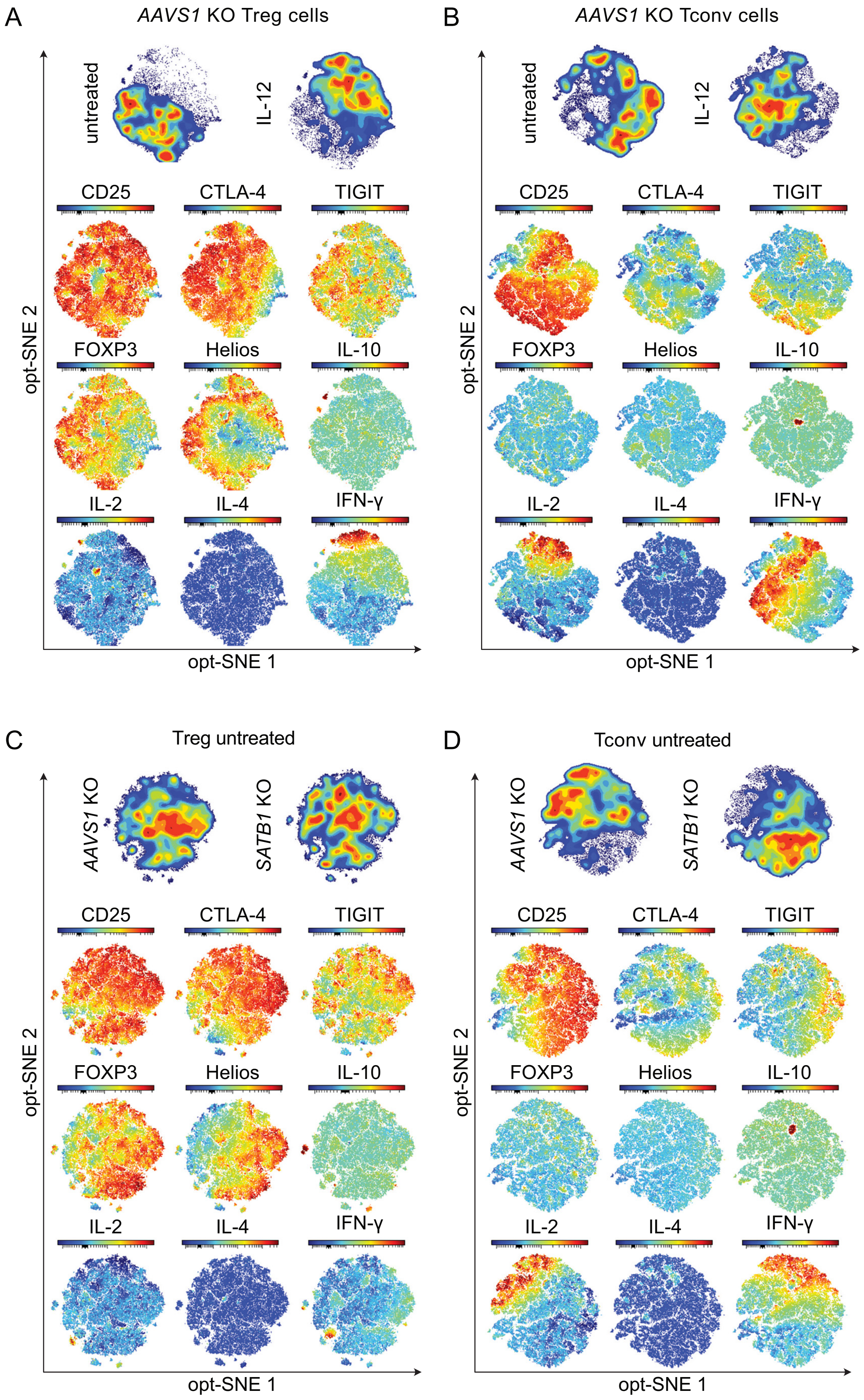

A
AAVS1 KO Treg cells
untreated
IL-12
CD25
CTLA-4
TIGIT
FOXP3
Helios
IL-10
IL-2
IL-4
IFN-γ
opt-SNE 2
opt-SNE 1
B
AAVS1 KO Tconv cells
untreated
IL-12
CD25
CTLA-4
TIGIT
FOXP3
Helios
IL-10
IL-2
IL-4
IFN-γ
opt-SNE 2
opt-SNE 1
C
Treg untreated
AAVS1 KO
SATB1 KO
CD25
CTLA-4
TIGIT
FOXP3
Helios
IL-10
IL-2
IL-4
IFN-γ
opt-SNE 2
opt-SNE 1
D
Tconv untreated
AAVS1 KO
SATB1 KO
CD25
CTLA-4
TIGIT
FOXP3
Helios
IL-10
IL-2
IL-4
IFN-γ
opt-SNE 2
opt-SNE 1

 © The Author(s)

Figure EV2. Integrated analysis of protein changes in *AAVS1* and *SATB1* KO Treg and Tconv cells based on flow cytometry.

(**A**, **B**) opt-SNE plot of untreated or IL-12-treated *AAVS1* KO Treg (**C**) and Tconv cells (**D**). (**C**, **D**) Expression levels (MFI) of tested flow cytometry markers of untreated *AAVS1* KO and *SATB1* KO Treg (**A**) and Tconv cells (**B**) plotted on opt-SNE density plot. *n*(Treg) = 12–17 (biological replicates), *n*(Tconv) = 12–18 (biological replicates).

© The Author(s)

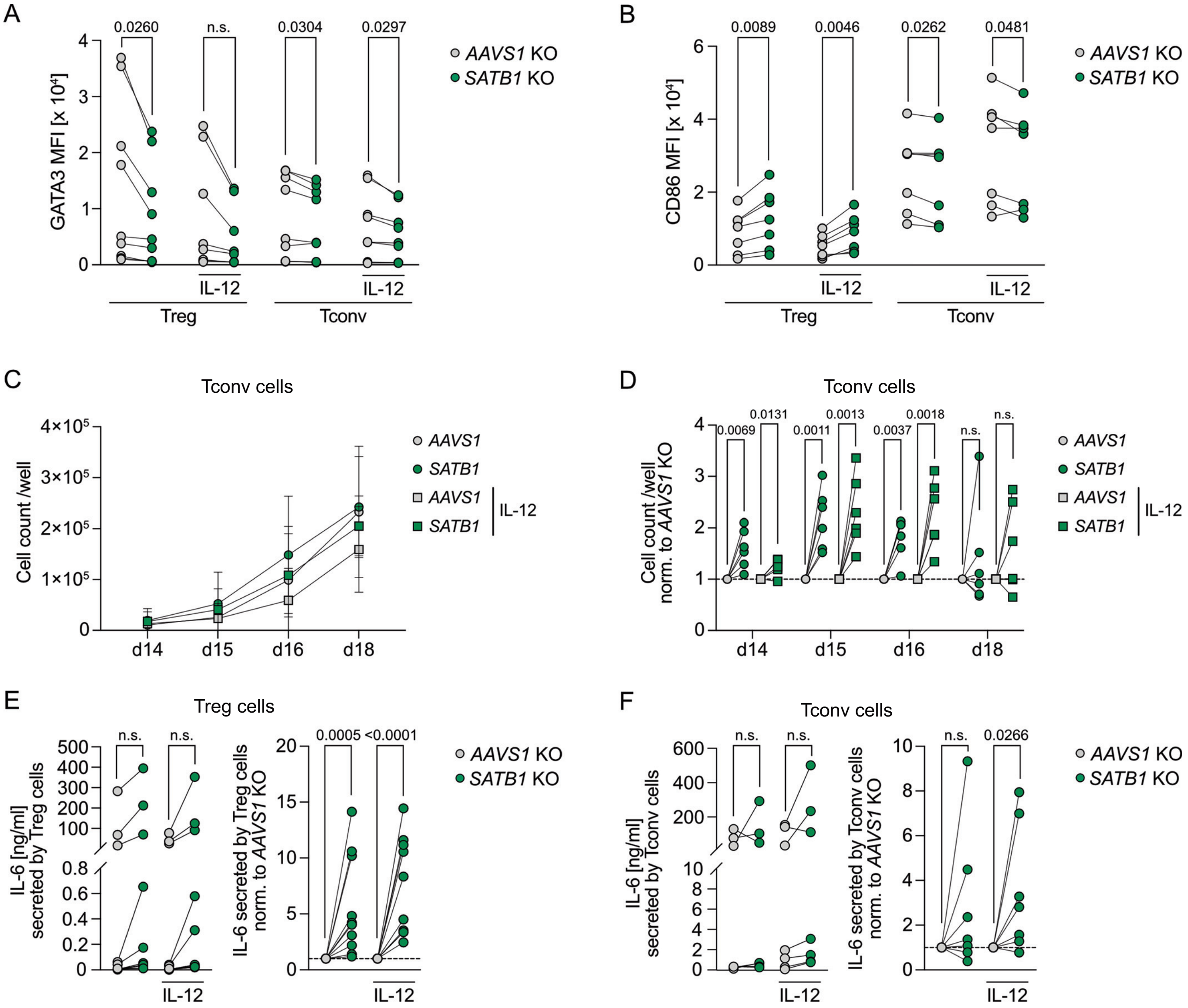

Figure EV3. GATA3 and CD86 expression of *SATB1* KO and *AAVS1* control KO Treg and Tconv cells.

(**A**) Mean fluorescence intensity (MFI) of GATA3 expression of *SATB1* KO and *AAVS1* control KO Treg and Tconv cells treated with or without IL-12. $n = 6–9$ (biological replicates), paired *t* test. (**B**) Mean fluorescence intensity (MFI) of CD86 and FOXP3 expression of *SATB1* KO and *AAVS1* control KO Treg and Tconv cells treated with or without IL-12. $n = 8–9$ (biological replicates), paired *t* test. (**C**, **D**) *AAVS1* and *SATB1* KO Tconv cell expansion rate at day 14, 15, 16, and 18 of culture with or without the addition of IL-12. (**C**) Absolute cell counts with mean and SD, (**D**) cell counts of *SATB1* KO Tconv cells normalized to respective *AAVS1* KO control cells. $n = 6$ (biological replicates). (**E**, **F**) Extracellular IL-6 levels determined by LEGENDplex™ assay of IL-12-treated *AAVS1* KO and *SATB1* KO Treg (**E**) or Tconv cells (**F**). Absolute values (left), normalized to *AAVS1* KO controls (right), $n = 7–10$ (biological replicates), paired *t* test.

 © The Author(s)

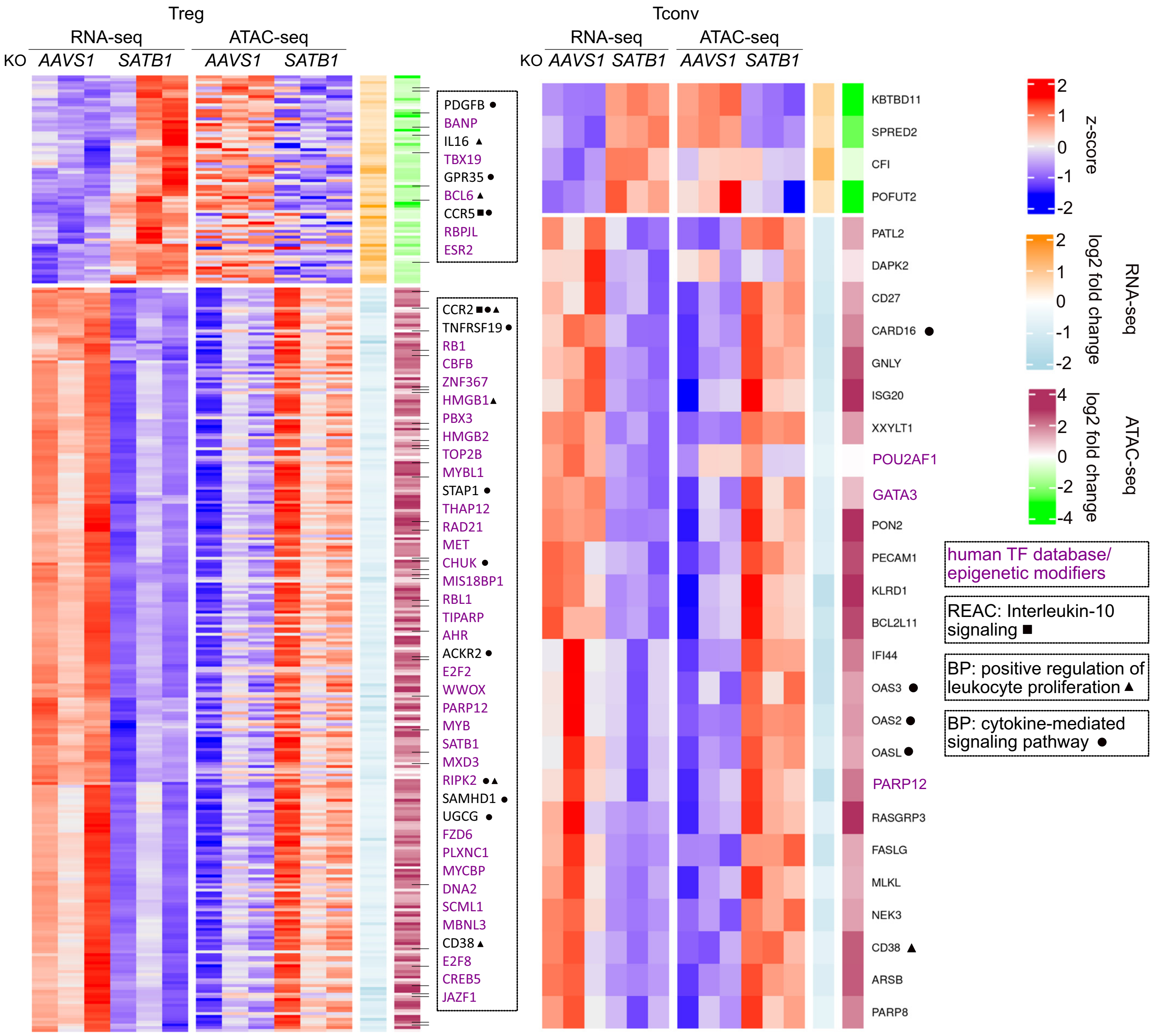

Figure EV4. Genes differentially regulated on chromatin and transcription levels in *SATB1* KO Treg and Tconv cells.

Heatmaps display z-scores of RNA- and ATAC-seq data of *SATB1* KO and *AAVS1* KO Treg and Tconv cells treated with IL-12. TFs differently regulated in RNA- and ATAC-seq data after *SATB1* KO are highlighted in purple. Genes associated with "Interleukin-10 signaling", "Positive regulation of leukocyte proliferation", or "cytokine-mediated signaling" are highlighted. REAC Reactome, BP biological pathway. $n = 3$ (biological replicates).
