# Supplementary material for: SATB1 is a targetable modulator of JAK-STAT signaling and cytokines in human Treg and Tconv cells
Source: EMBO Rep. 2026 Jun 12;27(14):4029–53. doi: 10.1038/s44319-026-00812-6 (PMC13400746; doi:10.1038/s44319-026-00812-6)
Supplement: Supplementary file 1 — Peer Review File [file 44319_2026_812_MOESM1_ESM.pdf]

EMBO reports

SATB1 is a targetable modulator of JAK-STAT signaling and cytokines in human Treg and Tconv cells

Saskia Kolb, Leonie Diekmann, Elizabeth Lochert, Linda Warmuth, Julia Ritter, Gunter Schmidtke, Michael Weber, Markus Hoffmann, Markus List, Daniel Kotlarz, Isabelle Serr, Carolin Daniel, Dirk Busch, Christian Schmidl, and Kathrin Schumann

Corresponding author(s): Kathrin Schumann (kathrin.schumann@uni-konstanz.de)

Review Timeline:

Submission Date:	1st Dec 25
Editorial Decision:	2nd Jan 26
Revision Received:	31st Mar 26
Editorial Decision:	30th Apr 26
Revision Received:	7th May 26
Accepted:	19th May 26

Editor: Achim Breiling

Transaction Report:

(Note: With the exception of the correction of typographical or spelling errors that could be a source of ambiguity, letters and reports are not edited. Depending on transfer agreements, referee reports obtained elsewhere may or may not be included in this compilation. Referee reports are anonymous unless the Referee chooses to sign their reports.)

Dear Prof. Schumann,

Thank you for the submission of your manuscript to EMBO reports. I have now received reports from the three referees that were asked to evaluate your study, which can be found at the end of this email. As you will see, the referees have several comments, concerns, and suggestions, indicating that a major revision of the manuscript is necessary to allow publication of the study in EMBO reports. As the reports are below, I will not detail them here.

Given the constructive referee comments, I would thus like to invite you to revise your manuscript with the understanding that the concerns of the referees must be addressed in the revised manuscript and/or in a detailed point-by-point response. Acceptance of your manuscript will depend on a positive outcome of a second round of review. It is EMBO reports policy to allow a single round of revision only and acceptance of the manuscript will therefore depend on the completeness of your responses included in the next, final version of the manuscript.

Revised manuscripts should be submitted within three months of a request for revision. Please contact me to discuss the revision (also by video chat) if you have questions or comments regarding the revision, or should you need additional time.

When submitting your revised manuscript, please also carefully review the instructions that follow below.

PLEASE NOTE THAT upon resubmission revised manuscripts are subjected to an initial quality control prior to exposition to re-review. Upon failure in the initial quality control, the manuscripts are sent back to the authors, which may lead to delays. Frequent reasons for such a failure are the lack of the data availability section (please see below) and the presence of statistics based on n=2 (the authors are then asked to present scatter plots or provide more data points).

When submitting your revised manuscript, we will require:

1) a .docx formatted version of the final manuscript text (including legends for main figures, EV figures and tables), but without the figures included. Figure legends should be compiled at the end of the manuscript text.

2) individual production quality figure files as .eps, .tif, .jpg (one file per figure), of main figures and EV figures. Please upload these as separate, individual files upon re-submission.

The Expanded View format, which will be displayed in the main HTML of the paper in a collapsible format, has replaced the Supplementary information. You can submit up to 6 images as Expanded View. Please follow the nomenclature Figure EV1, Figure EV2 etc. The figure legend for these should be included in the main manuscript document file in a section called Expanded View Figure Legends after the main Figure Legends section. Additional Supplementary material should be supplied as a single pdf file labeled Appendix. The Appendix should have page numbers and needs to include a table of content on the first page (with page numbers) and legends for all content. Please follow the nomenclature Appendix Figure Sx, Appendix Table Sx etc. throughout the text, and also label the figures and tables according to this nomenclature.

For more details, please refer to our guide to authors:
https://link.springer.com/journal/44319/submission-guidelines#cms-Revised-submissions

Please consult our guide for figure preparation:
https://media.springernature.com/original/springer-cms/rest/v1/content/27825798/data/v1

3) a .docx formatted letter INCLUDING the reviewers' reports and your detailed point-by-point responses to their comments. As part of the EMBO Press transparent editorial process, the point-by-point response is part of the Review Process File (RPF), which will be published alongside your paper.

4) a complete author checklist, which you can download from our author guidelines (https://link.springer.com/journal/44319/submission-guidelines#cms-Revised-submissions). Please insert page numbers in the checklist to indicate where the requested information can be found in the manuscript. The completed author checklist will also be part of the RPF.

Please also follow our guidelines for the use of living organisms, and the respective reporting guidelines:
https://link.springer.com/partners/embo-press/editorial-policies#Animal%20and%20human%20studies

5) that primary datasets produced in this study (e.g. RNA-seq, ChIP-seq, structural and array data) are deposited in an appropriate public database. If no primary datasets have been deposited, please also state this in a dedicated section (e.g. 'No primary datasets have been generated and deposited'), see below.

See also: https://link.springer.com/partners/embo-press/editorial-policies#Data%20deposition

Please remember to provide a reviewer password if the datasets are not yet public.

The accession numbers and database should be listed in a formal "Data Availability" section that follows the model below. This is now mandatory (like the COI statement). Please note that the Data Availability Section is restricted to new primary data that are part of this study. This section is mandatory. As indicated above, if no primary datasets have been deposited, please state this in this section

Data availability

The datasets produced in this study are available in the following databases:

- RNA-Seq data: Gene Expression Omnibus GSE46843 (https://www.ncbi.nlm.nih.gov/geo/query/acc.cgi?acc=GSE46843)
- [data type]: [name of the resource] [accession number/identifier/doi] ([URL or identifiers.org/DATABASE:ACCESSION])

*** Note - All links should resolve to a page where the data can be accessed. ***

See also: https://link.springer.com/partners/embo-press/editorial-policies#Data%20availability%20statement

Moreover, I have these editorial requests:

6) We now request the publication of original source data with the aim of making primary data more accessible and transparent to the reader. You will receive a separate email with instructions for providing source data with your revised manuscript, including information how to upload and organize the files.

7) Our journal encourages inclusion of *data citations in the reference list* to directly cite datasets that were re-used and obtained from public databases. Data citations in the article text are distinct from normal bibliographical citations and should directly link to the database records from which the data can be accessed. In the main text, data citations are formatted as follows: "Data ref: Smith et al, 2001" or "Data ref: NCBI Sequence Read Archive PRJNA342805, 2017". In the Reference list, data citations must be labeled with "[DATASET]". A data reference must provide the database name, accession number/identifiers and a resolvable link to the landing page from which the data can be accessed at the end of the reference. Further instructions are available at: https://link.springer.com/journal/44319/submission-guidelines#cms-Reference-guidelines

8) Regarding data quantification and statistics, please make sure that the number "n" for how many independent experiments were performed, their nature (biological versus technical replicates), the bars and error bars (e.g. SEM, SD) and the test used to calculate p-values is indicated in the respective figure legends (also for EV and Appendix figures). Please also check that all the p-values are explained in the legend, and that these fit to those shown in the figure. Please provide statistical testing where applicable. Please avoid the phrase 'independent experiment' but clearly state if these were biological or technical replicates. Please also indicate (e.g. with n.s.) if testing was performed, but the differences are not significant. In case n=2, please show the data as separate datapoints without error bars and statistics. See also: https://link.springer.com/journal/44319/submission-guidelines#cms-Figure-and-data-presentation

If n<5, please show single datapoints for diagrams.

9) Please add scale bars of similar style and thickness to all microscopic images, using clearly visible black or white bars (depending on the background). Please place these in the lower right corner of the images themselves. Please do not write on or near the bars in the image but define the size in the respective figure legend.

10) Please also note our reference format: https://link.springer.com/journal/44319/submission-guidelines#cms-Reference-guidelines

11) We updated our journal's competing interests policy in January 2022 and request authors to consider both actual and perceived competing interests. Please review the policy https://www.embopress.org/competing-interests and update your competing interests if necessary. Please name this section 'Disclosure and Competing Interests Statement' and put it after the Acknowledgements section.
See: https://link.springer.com/partners/embo-press/editorial-policies#Competing%20interest%20disclosures

12) We now use CRediT to specify the contributions of each author in the journal submission system. CRediT replaces the author contribution section. Please use the free text box to provide more detailed descriptions and do NOT provide your final manuscript text file with an author contributions section. See also our guide to authors (section 'Author contributions'): https://link.springer.com/journal/44319/submission-guidelines#cms-Revised-submissions

13) All Materials and Methods need to be described in the main text using our 'Structured Methods' format, which is required for all research articles. According to this format, the Methods section should include a Reagents and Tools Table (listing key

reagents, experimental models, software, and relevant equipment and including their sources and relevant identifiers), uploaded as separate file, and a Methods section in which we encourage the authors to describe their methods using a step-by-step protocol format with bullet points, to facilitate the adoption of the methodologies across labs. More information on how to adhere to this format as well as downloadable templates (.doc) for the Reagents and Tools Table can be found in our author guidelines (section 'Structured Methods'):

https://link.springer.com/journal/44319/submission-guidelines#cms-Manuscript-organisation-and-formatting

Please add all primer information directly to the Reagents and Tools table.

The pathway analysis (Tables S6?) is a dataset. Please name this and upload it as a Dataset file (e.g. Dataset EV1) and call it out like this. Please also add a legend for each on the first TAB of the excel file.

14) Please order the manuscript sections like this, using only these names:
Title page - Abstract (max. 175 words) - Keywords (max. 5) - Introduction - Results - Discussion - Methods - Data availability section - Acknowledgements (please include here all the funding information) - Disclosure and Competing Interests Statement - References - Figure legends - Expanded View Figure legends

15) Please make sure that all the funding information is also entered into the online submission system and that it is complete and similar to the one in the acknowledgement section of the manuscript text file.

We would also welcome the submission of cover suggestions, or motifs to be used by our Graphics Illustrator in designing a cover.

As part of the EMBO publication's Transparent Editorial Process, EMBO reports publishes online a Review Process File (RPF) to accompany accepted manuscripts. This File will be published in conjunction with your paper and will include the referee reports, your point-by-point response and all pertinent correspondence relating to the manuscript.

You are able to opt out of this by letting the editorial office know (emboreports@embo.org). If you do opt out, the Review Process File link will point to the following statement: "No Review Process File is available with this article, as the authors have chosen not to make the review process public in this case."

I look forward to seeing a revised form of your manuscript when it is ready.

Yours sincerely,

Achim Breiling
Senior Editor
EMBO Reports

Referee #1:

In this manuscript by Kolb and colleagues, the authors take a reverse genetics approach to investigate the implications of SATB1 knockout in human Treg and Tconv cells, particularly in inflammatory IL-12-exposed conditions. The authors perform comprehensive profiling of control and perturbed cells by flow cytometry, ATAC-seq, and RNA-seq, and identify particularly strong effects on cytokine signaling and production pathways in Tregs. The authors hypothesize that based on the gene signatures SATB1 knockout Tregs have reduced, but CD4+ Tconv cells have increased function. The authors then show that Tregs do indeed have increased function in an in vitro suppression assay and CD4+ CAR T cells have modestly improved performance in an in vivo tumor control experiment.

This is a well thought-out and executed study, with appropriate controls and mostly appropriate conclusions throughout. Furthermore, it is well written and organized, with nice figure quality. At this point I do not recommend the need for any further experimentation, but just minor corrections/clarifications in the text, analysis, and figures outlined below.

Minor points:

• I found these sentences in the introduction confusing. If SATB1 is a positive regulator of FOXP3, wouldn't one expect overexpression to increase immunosuppression / decrease inflammatory cytokines? I feel like this paradox should be explained. "Interestingly, in fully differentiated human Treg cells, SATB1 is a positive regulator of FOXP3 expression (Schumann et al, 2020). Foxp3/FOXP3 is counteracting this upregulation by inducing microRNAs, which in turn downregulate SATB1 mRNA levels in human and murine Treg cells indicating tight regulation for proper cellular function (Beyer et al, 2011). Overexpression of SATB1 in Treg cells results in increased levels of multiple pro-inflammatory cytokines associated with Th1, Th2, and Th17

effector functions, which overall resulted in reduced suppressive capacity (Beyer et al., 2011; Burute et al, 2012)."
• I find the t-SNE representations in Figs. 1C-D difficult to interpret and ineffective. Box or violin plots comparing the groups for each marker would be far more effective. Two-way scatter plots would be more effective at identifying co-expressed markers if the authors are trying to make that point.
• In figures 1F, 1H it would be nice to put Treg or Tconv somewhere in the figure so the reader does not have to refer to the legend as much
• I am not sure Fig. 1H is called out in the text. The authors conclude the first results section with "Overall, SATB1 ablation in Treg and Tconv cells resulted in subset-specific regulation of the pro-inflammatory cytokines and a general boost in IL-10 production", however it looks like that is not the case for Tconv according to 1H
• In figure 2 it would be nice to see a scatter plot comparing LFC(SATB1ko/AAVS1ko) in Tconv and LFC(SATB1ko/AAVS1ko) in Treg for all ~3000 DEGs (or the entire transcriptome). That would be a bit nicer than somewhat arbitrary significance cutoffs to show that the regulated gene profiles are indeed very different between the two cell types.
• Is there a stimulation done at the time of CFSE staining in figure 5? It would be helpful to show that in the schematic
• Figure 6B. Is this statistically significant? It would be better to show this as paired representations (e.g. as in Fig. 1F) and show absolute expansion values with paired T tests (as opposed to normalizing everything)

Referee #2:

This study presents a comprehensive analysis of the role of the SATB1 transcription factor in conventional T cells. The research group has strong expertise in both the analytical methodologies employed and the manipulation of human T cells and Tregs. Functionally, the authors show enhanced tumor cell clearance mediated by SATB1-knockout $CD4^+$ T cells, thereby highlighting novel opportunities for the development and optimization of T cell-based immunotherapies. I have some suggestions which may improve the clarity and quality of the manuscript.

Major Comments:
It would be interesting, at least in the AAVS1 knockout (KO) condition, to draw conclusions about the effect of IL-12 on Tregs, as this may be a critical confounding factor in this study.

Could the authors comment on whether the lack of cytokine production observed in SATB1 KO conventional T cells (Tconv) is related to the fact that these cells appear to be already activated at baseline and therefore respond poorly to PMA/ionomycin stimulation? It would be informative to report expansion folds upon restimulation and, more importantly, to monitor CD25 expression over time. Repeating the experiments when cells become CD25low (if applicable) could be considered. In this context, did SATB1 KO Tconv expand faster than AAVS1 KO Tconv?

The authors nicely demonstrate upregulation of CD25 in Tconv but not in Tregs. Have they analyzed CD25 mean fluorescence intensity (MFI), given that Tregs constitutively express CD25?

The authors report a strong upregulation of the IL-6 cytokine pathway. A sub-analysis correlating these transcriptomic findings with IL-6 protein detection in the supernatant, as well as modulation of the JAK-STAT pathway, would strengthen the conclusions. Such mechanisms may contribute to the observed reduction in Treg suppressive function.

Minor Comments:
It would be helpful for the reader if the authors explicitly described how SATB1 knockout efficiency was assessed in the Results section.

Can the authors confirm whether the heatmap displaying fold changes of pro- and anti-inflammatory flow cytometry markers in SATB1 KO Tregs and SATB1 KO Tconv was normalized to AAVS1 KO control Tregs and Tconv, respectively?

Were Tregs cultured with IL-2 between days 12 and 15?

In Figure 1C, the subpopulation of Tregs producing IL-2 represents only a minority of the opt-SNE contour plot and appears FOXP3-negative. Could this reflect contamination by effector T cells?

In Figure 1E, given the variability in the data, should the results be paired by donor?

It would be helpful to clearly state in the Results section that PMA/ionomycin stimulation was used, as this could represent a confounding factor.

The statement, "Overall, SATB1 ablation in Treg and Tconv cells resulted in subset-specific regulation of pro-inflammatory cytokines and a general boost in IL-10 production," appears to be an overinterpretation, as only a very small subset of cells produces IL-10 (based on Figure 1B).

Typographical correction needed: "SATB1 KO Tconv cellschanges" → add a space between "cells" and "changes."

A more detailed discussion of the opposing results observed between murine and human SATB1 KO Treg cells would strengthen the manuscript.

A limitations paragraph should be included in the Discussion section. For example:
CD8 T cells were not analyzed (particularly in vivo), which limits conclusions regarding the suitability of targeting SATB1 in multiple T cell subsets for immunotherapy.
Differentiation and exhaustion profiles of CAR-T cells were not assessed in vivo.
Flow cytometry, ATAC-seq, and RNA-seq analyses were performed exclusively after PMA/ionomycin stimulation, with no comparison to other conditions (e.g., no stimulation, anti-CD3/CD28 bead stimulation, or CAR-specific stimulation).

Referee #3:

This study provides a thorough and well-executed analysis of SATB1 function in human CD4 T cell subsets, with a particular focus on Treg and CD4 CAR T cells. The authors convincingly demonstrate that SATB1 acts as a key regulator of subset-specific gene programs, controlling cytokine expression and JAK-STAT signaling in a cell-type-specific manner. Functionally, SATB1 ablation weakens Treg suppressive activity while enhancing CD4 CAR T cell-mediated tumor clearance in a humanized mouse model, highlighting the translational relevance of modulating SATB1 to improve CAR T therapy. The work is strengthened by a multi-layered dataset, including chromatin, gene expression, and functional analyses, which together robustly support the conclusions.

With modest revisions, the manuscript could be even more accessible and precise. Specifically, grammatical errors (e.g., "Despite of the distinct gene-regulatory patterns") and typographical issues (e.g., "cellschanges," spacing inconsistencies, truncated labels) should be corrected. Quantitative descriptions should accompany terms like "slight" to improve clarity, and terminology should be consistent across figures and legends (e.g., "Tconv" vs. "Teff"). Finally, statistical annotations would benefit from briefly restating the tests used in figure legends when multiple tests are presented. Overall, this is a strong and impactful study with clear mechanistic insights and translational relevance.

Overall: We thank all reviewers for their time and their helpful comments. By addressing their suggestions and recommendations, we believe that the revised version of our manuscript has further gained in strength as well as clarity.
The revised manuscript now includes additional experimental data showing:

- Quantification of IL-6 protein levels in the supernatants of *AAVS1* and *SATB1* KO Treg and Tconv cells, showing an upregulation of IL-6 production after SATB1 ablation.
- An increased proliferation rate of *SATB1* KO Tconv cells with or without IL-12 stimulation compared to *AAVS1* KO control cells.
- We also added a more detailed analysis of the impact of IL-12 on *AAVS1* KO control Treg cells based on flow cytometry data.

We additionally provide in the response letter data to the modulation of the JAK-STAT signaling pathway after SATB1 ablation in Treg and Tconv cells, CD25 expression levels in *SATB1* and *AAVS1* KO Tconv cells as well as the response of *AAVS1* and *SATB1* KO Tconv cells to PMA/Iono restimulation.

Referee #1:

In this manuscript by Kolb and colleagues, the authors take a reverse genetics approach to investigate the implications of SATB1 knockout in human Treg and Tconv cells, particularly in inflammatory IL-12-exposed conditions. The authors perform comprehensive profiling of control and perturbed cells by flow cytometry, ATAC-seq, and RNA-seq, and identify particularly strong effects on cytokine signaling and production pathways in Tregs. The authors hypothesize that based on the gene signatures SATB1 knockout Tregs have reduced, but CD4+ Tconv cells have increased function. The authors then show that Tregs do indeed have increased function in an in vitro suppression assay and CD4+ CAR T cells have modestly improved performance in an in vivo tumor control experiment.

This is a well thought-out and executed study, with appropriate controls and mostly appropriate conclusions throughout. Furthermore, it is well written and organized, with nice figure quality. At this point I do not recommend the need for any further experimentation, but just minor corrections/clarifications in the text, analysis, and figures outlined below.

We thank the reviewer for this very positive evaluation of our work.

Minor points:

• I found these sentences in the introduction confusing. If SATB1 is a positive regulator of FOXP3, wouldn't one expect overexpression to increase immunosuppression / decrease inflammatory cytokines? I feel like this paradox should be explained. "Interestingly, in fully differentiated human Treg cells, SATB1 is a positive regulator of FOXP3 expression (Schumann et al, 2020). Foxp3/FOXP3 is counteracting this upregulation by inducing microRNAs, which in turn downregulate SATB1 mRNA levels in human and murine Treg cells indicating tight regulation for proper cellular function (Beyer et al, 2011). Overexpression of

SATB1 in Treg cells results in increased levels of multiple pro-inflammatory cytokines associated with Th1, Th2, and Th17 effector functions, which overall resulted in reduced suppressive capacity (Beyer et al., 2011; Burute et al, 2012)."

We thank the reviewer for pointing this out. We revised this section for clarity.

• I find the t-SNE representations in Figs. 1C-D difficult to interpret and ineffective. Box or violin plots comparing the groups for each marker would be far more effective. Two-way scatter plots would be more effective at identifying co-expressed markers if the authors are trying to make that point.

We chose multidimensional opt-SNE plots to integrate all nine flow cytometry markers tested to detect phenotypes of subpopulations also defined by more than two markers and how these cell populations shift between *AAVS1* KO and *SATB1* KO conditions. In this data representation, a significant population of *SATB1* KO Treg cells were defined by lower levels of FOXP3 and CTLA-4, however, IL-2- secreting and IFNγ-secreting KO Treg cells clustered separately in two distinct subpopulations. *SATB1* KO Treg cells were defined by lower levels of FOXP3, CTLA-4 and Helios, and higher IL-2 (Fig. 1C). *SATB1* KO Tconv cells depicted higher levels of TIGIT and reduced IL-2 and IFNγ (Fig. 1D). We changed the language in the results section of the manuscript to better explain these changes in cell subpopulations. Additionally, bar graph plots of all makers in all tested conditions can be found in Figure EV1.

• In figures 1F, 1H it would be nice to put Treg or Tconv somewhere in the figure so the reader does not have to refer to the legend as much.

Thanks for pointing this out. We included these labels in the revised Figure 1 (now Figure 1E-H).

• I am not sure Fig. 1H is called out in the text. The authors conclude the first results section with "Overall, SATB1 ablation in Treg and Tconv cells resulted in subset-specific regulation of the pro-inflammatory cytokines and a general boost in IL-10 production", however it looks like that is not the case for Tconv according to 1H

We added the missing reference in the text. We added data of 4 additional donors to analyze IL-10 phenotypes after *SATB1* ablation in more detail. *SATB1* KO Treg cells with or without IL-12 stimulation show a significant increase in IL-10 production compared with *AAVS1* KO Treg cells. Also, in *SATB1* KO Tconv cells we see an increase in IL-10 production without the addition of IL-12, however, after IL-12 treatments we see only in some of the donors an increase in IL-10 production. We discuss this effect now in more detail and overall revised the language in this section. We also display now both absolute and normalized values (now Figure 1F and 1H).

• In figure 2 it would be nice to see a scatter plot comparing LFC(SATB1ko/AAVS1ko) in Tconv and LFC(SATB1ko/AAVS1ko) in Treg for all ~3000 DEGs (or the entire transcriptome). That would be a bit nicer than somewhat arbitrary significance cutoffs to show that the regulated gene profiles are indeed very different between the two cell types.

The reviewer is asking about the data display in Figure 2 and suggests a comparison between LFC(SATB1ko/AAVS1ko) in Tconv and LFC(SATB1ko/AAVS1ko) in Treg for all DEGs. The significance cutoff was set to the arbitrary, but scientifically accepted standard of $p < 0.05$. We had to set a cutoff for our data to reduce data background. No cut offs for log2 fold changes were applied. We tested the suggested data presentation style (shown in Figure Letter 1). By using this strategy, we cannot fully convey the differences between the two cell types, as many of DEGs are only differentially expressed in one of the cell types and are therefore located on the x- and y-axis. However, here we also show that most overlapping DEGs between both cell types are coregulated on mRNA level, rather than contrary regulated which is in accordance to Figure 2D. Additionally, in Figure 3E and Figure EV4 we show equally or differentially regulated chromatin and gene expression changes in *SATB1* KO and *AAVS1* KO Treg and Tconv cells to give a global overview over the global differences in these two cell subsets after *SATB1* KO.

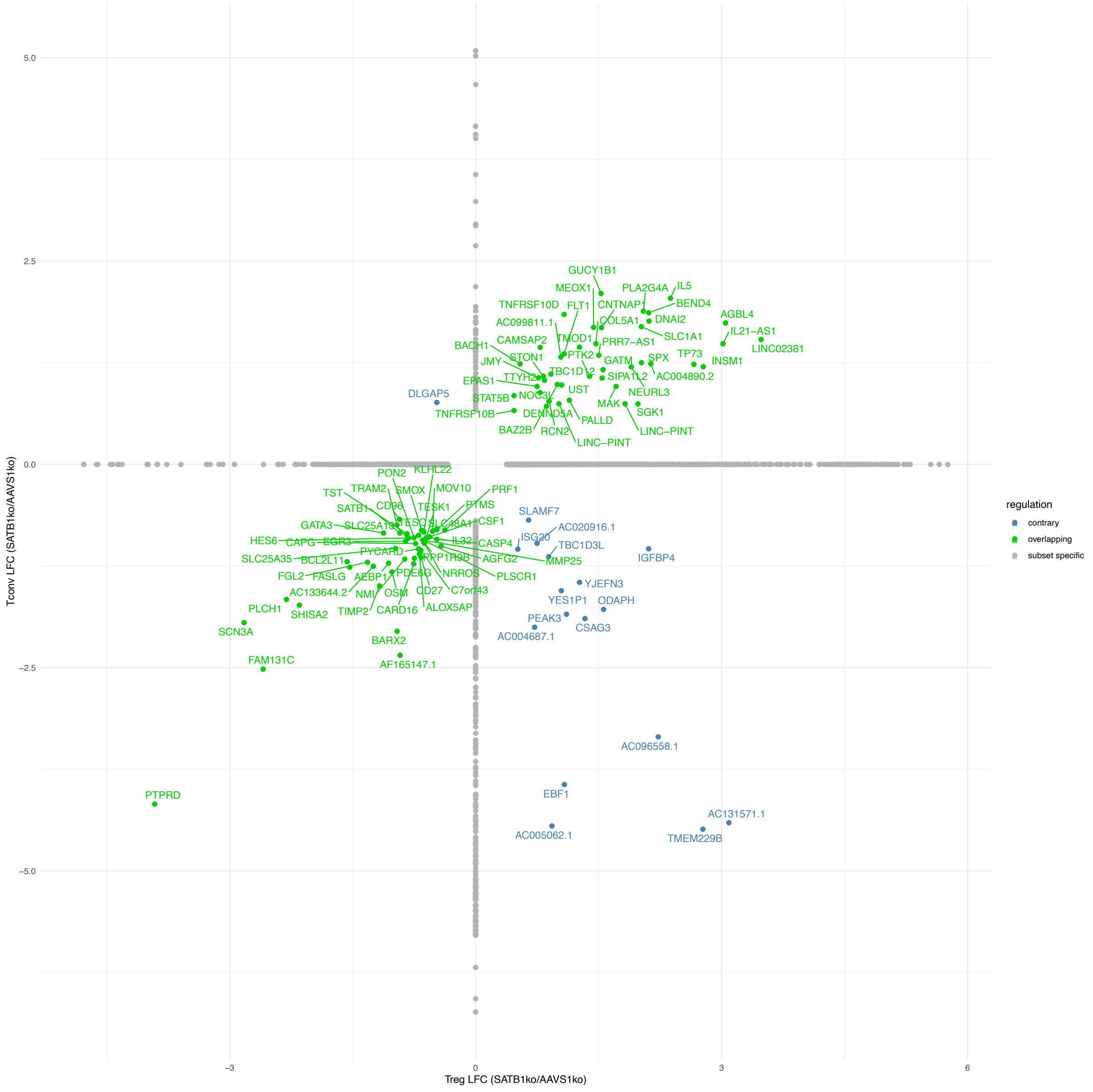

Figure Letter 1: Log2 fold change (LFC) of significantly differently expressed genes ($p < 0.05$) between SATB1 KO Treg and Tconv compared to their respective AAVS1 KO control.

• *Is there a stimulation done at the time of CFSE staining in figure 5? It would be helpful to show that in the schematic.*

We thank the reviewer for raising this point. We now added this information to Figure 5A for improved clarity.

• Figure 6B. Is this statistically significant? It would be better to show this as paired representations (e.g. as in Fig. 1F) and show absolute expansion values with paired T tests (as opposed to normalizing everything)

We thank the reviewer for this suggestion. We depict in Figure Letter 2 the absolute values and in Figure 6B the normalized values in the paired representations. We chose to show in the manuscript the normalized, paired data as we think it shows the increase of CD4 Tconv cell numbers due to SATB1 ablation more clearly.

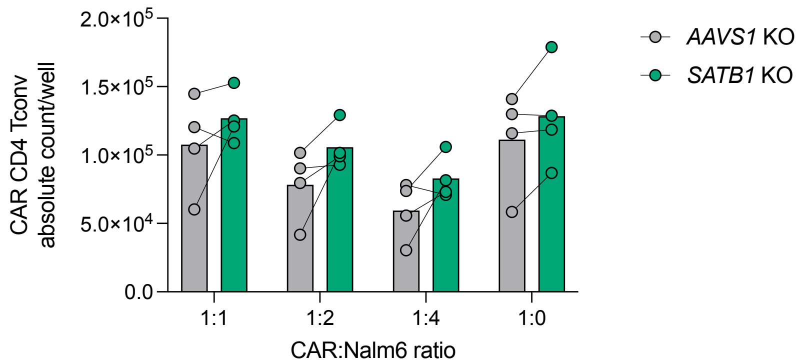

Figure Letter 2: Absolute counts of CAR CD4 Tconv cells (corresponds to Figure 6B). n = 4 (biological replicates)

Referee #2:

This study presents a comprehensive analysis of the role of the SATB1 transcription factor in conventional T cells. The research group has strong expertise in both the analytical methodologies employed and the manipulation of human T cells and Tregs. Functionally, the authors show enhanced tumor cell clearance mediated by SATB1-knockout $CD4^+$ T cells, thereby highlighting novel opportunities for the development and optimization of T cell-based immunotherapies. I have some suggestions which may improve the clarity and quality of the manuscript.

We thank the reviewer for critically evaluating our manuscript and the instrumental suggestions and comments.

Major Comments:

It would be interesting, at least in the AAVS1 knockout (KO) condition, to draw conclusions about the effect of IL-12 on Tregs, as this may be a critical confounding factor in this study.

The reviewer is raising an important point - we mimic a proinflammatory microenvironment in a reductionistic manner by adding IL-12 which also affects Treg cell phenotypes. IL-12 has been widely described as an inducer of Th1-like Treg cells with distinct features such as the increased production of IFNγ and the induction of Tbet (Dominguez-Villar et al., 2011 *Nature Medicine*, Daniel et al., 2015 *BMC Immunol*, Koenecke et al., 2012 *J Immunol*, Schumann et al., *Nature Immunology* 2020). To analyze in more detail the impact of IL-12 in our system on Treg cells, we reanalyzed the FACS data shown in Figure 1 with the multidimensional analysis platform OMIQ. *AAVS1* KO Treg cells treated with IL-12 form a largely separate cluster compared to *AAVS1* KO Treg cells only treated with IL-2 (shown in Figure EV2C, D). This distinction is mainly driven by the induction of IFNγ by IL-12 We discuss now also in more detail the impact of IL-12 on Treg cell function.

Could the authors comment on whether the lack of cytokine production observed in SATB1 KO conventional T cells (Tconv) is related to the fact that these cells appear to be already activated at baseline and therefore respond poorly to PMA/ionomycin stimulation? It would be informative to report expansion folds upon restimulation and, more importantly, to monitor CD25 expression over time. Repeating the experiments when cells become CD25low (if applicable) could be considered. In this context, did SATB1 KO Tconv expand faster than AAVS1 KO Tconv?

The reviewer is raising an interesting point: Is the changed activation status of *SATB1* KO Tconv negatively impacting their response towards PMA/Iono? To test this hypothesis, we expanded KO Tconv cells for up to 18 days and monitored the CD25 levels of the cells, their expansion rates and how these cells react to different restimulation conditions (antiCD3, antiCD3/28, PMA/Iono) over time (day 14, day 15, day 16 and day 18 (previously the readouts were performed only at day 15)). Because the cells have received several rounds of antiCD3/CD28 stimulation the majority of the Tconv cells express CD25, going in hand with relatively high MFI values for CD25 in *AAVS1* and *SATB1* KO Tconv cells. *SATB1* KO Tconv had slightly higher CD25 values until day 15, the standard readout in our protocols. After day 15, CD25 is slightly downregulated in all tested conditions (Figure Letter 3).

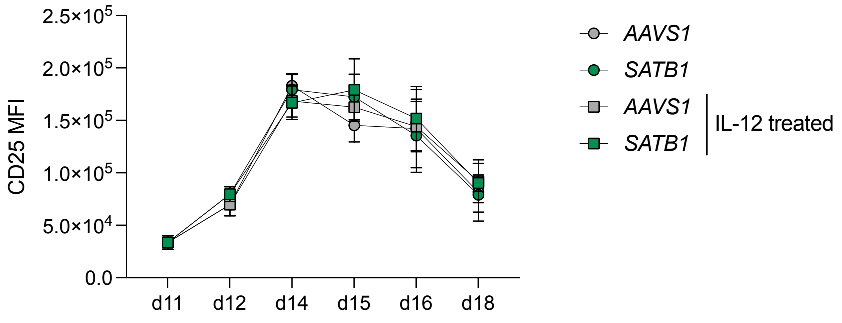

Figure Letter 3: CD25 MFI values of AAVS1 and SATB1 KO Tconv cells treated with or without IL-12. n = 6, biological replicates.

Cytokine analysis after different restimulation conditions at day 15, the readout day we applied throughout the manuscript, and day 18, a readout timepoint with lower CD25 expression in Tconv cells, showed an effect of PMA/Iono on all tested cytokines in all four tested stimulation conditions. At day 18 of culture, the cytokine production in *AAVS1* and *SATB1* KO Tconv with or without IL-12 incubation was lower. However, at day 18, PMA/ionomycin stimulation

resulted in strong IFNγ induction, accompanied by an increase in all other cytokines, albeit to a lesser extent. Therefore, we concluded that the relatively high activation status of the cells at our readout timepoint does not negatively affect the response of the *SATB1* KO Tconv cells towards PMA/Iono stimulation (Figure Letter 4).

We also evaluated the Tconv expansion rates at day 14, 15, 16 and day 18. Until day 16 *SATB1* KO Tconv have a proliferative advantage with or without IL-12 treatment. At day 18, these differences are not detectable anymore, potentially because of higher cell densities in the individual wells or a decrease in the overall activation status of the cells in control and SATB1 KO Tconv cells. The Tconv expansion rates are shown in Figure EV3C, D (absolute values and normalized values).

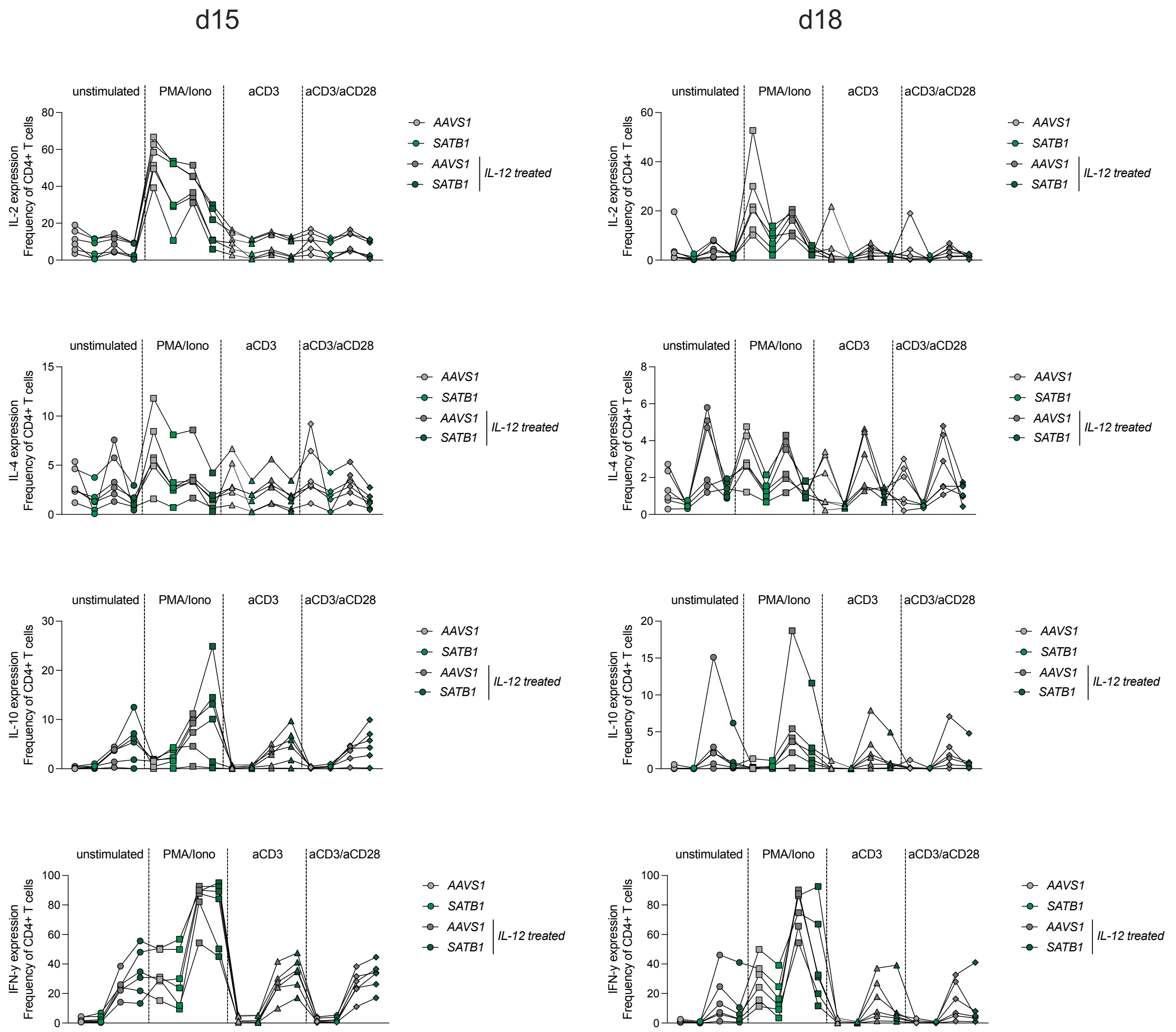

Figure Letter 4: AAVS1 and SATB1 KO Tconv cells control treated or IL-12 conditioned were restimulated on day 15 or day 18 of in vitro culture with PMA/Iono, antiCD3 or antiCD3/CD28 and intracellularly stained n = 6, biological replicates.

The authors nicely demonstrate upregulation of CD25 in Tconv but not in Tregs. Have they analyzed CD25 mean fluorescence intensity (MFI), given that Tregs constitutively express CD25?

Clustering in Figure 1C is based on the MFI values, in Figure 1B we show the normalized frequencies of CD25 positive cells and in figure EV1E, F the absolute frequencies. We show in Figure Letter 5 the CD25 MFI values for all tested conditions in bar graph plots. We observe in both data representations, % CD25-positive (Figure 1B) and CD25 MFI values (Figure Letter 5), the same tendencies in *SATB1* KO Treg cells compared to AAVS1 control cells - a slight reduction in the CD25 signal (significant only by MFI representation).

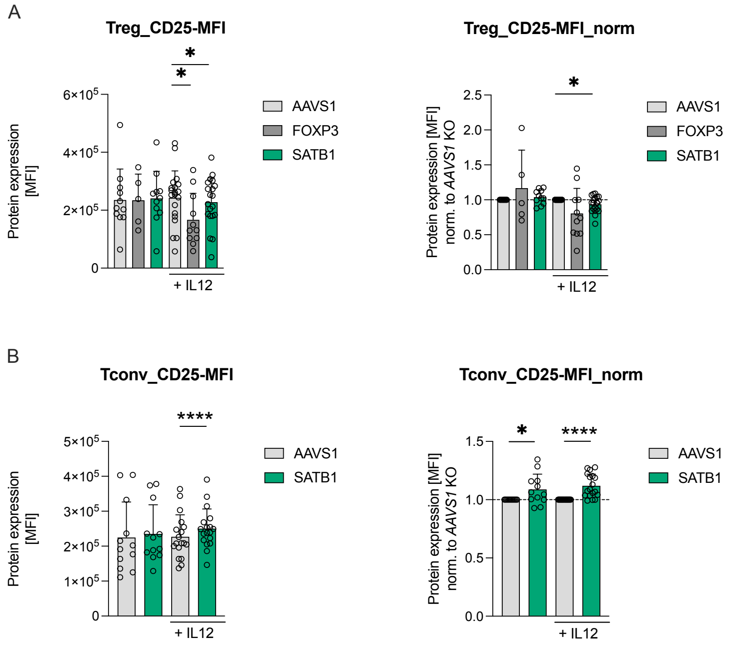

Figure Letter 5: CD25 MFI values of AAVS1, FOXP3 and SATB1 KO Treg (A) and Tconv cells (B. Left: absolute MFI values, right: SATB1 KO cells normalized to AAVS1 controls. n = 5-12, biological replicates.

The authors report a strong upregulation of the IL-6 cytokine pathway. A sub-analysis correlating these transcriptomic findings with IL-6 protein detection in the supernatant, as well as modulation of the JAK-STAT pathway, would strengthen the conclusions.Such mechanisms may contribute to the observed reduction in Treg suppressive function.

The reviewer is raising important points regarding the IL-6 protein levels and the downstream effects after *SATB1* ablation in Treg and Tconv cells. We now added Legendplex quantification of IL-6 levels in Figure EV3 E, F. In the supernatants of *SATB1* KO Treg and *SATB1* KO Tconv cells IL-6 levels are significantly increased compared to the respective *AAVS1* KO control cells, validating the strong changes of mRNA on protein level.

STAT3 transcript levels were upregulated in *SATB1* KO Treg cells and showed a very slight upregulation in *SATB1* KO Tconv cells. Active STAT3 serves as the main transcriptional regulator downstream of IL-6. We tested by western blot analysis if the observed changes in

mRNA expression levels result in a modulation of STAT3 activity, defined as the ratio of pSTAT3(Tyr705)/STAT3. Therefore, we treated KO Treg and Tconv cells with IL-2 only or together with IL-12 for 3 days, our standard conditions, or additionally with recombinant IL-6 for 1 h to boost STAT3 signaling. We detected in *SATB1* KO Treg a significantly reduced activity of STAT3 after short-term IL-6 stimulation (Figure Letter 6). The role of STAT3 in Treg cell function is highly context dependent. Stat3 regulates the balance between Treg and Th17 cells by suppressing Th17 cell differentiation and boosting Treg cell induction (Pallandre et al., 2007 J Immunol, Qin et al. 2024 J Exp Med). However, Stat3 inhibition can restore the balance between Treg and Tconv cells during chronic inflammation and can also negatively affect Treg stability (Aquel et al., 2021 JCI Insight, Laurence et al., 2012 Immunity). Discrepancies between mRNA levels and protein levels and activity have been previously reported in Treg cells (Cuadrado et al., 2017 Immunity). Besides that, JAK-STAT signaling is highly time dependent (Wang et al., 2013 PNAS). As we incubated *SATB1* KO Treg cells for 72 hrs with IL-12, we are probably not in the acute signaling phase anymore, and potentially detect downstream effects of STAT3 signaling such as changed cytokine expression. The complexity of the system is further increased by the possibility of further posttranslational modifications of STAT3. For example, phosphorylation at serine 727 boosts the transcriptional activity of STAT3 (Arevalo et al., 2023 Scientific Reports). Furthermore, we observed additional changes in JAK2, STAT1, STAT5 and STAT6 transcripts which can impact the experimental outcome. Therefore, we think that global analysis of the JAK-STAT signaling in the KO cells in a cytokine and time-dependent manner is needed, for example by phosphoproteomics. However, these experiments are beyond the scope of this current study and will be addressed in detail in the future in independent experiments. We added this important point to “limitation of our study”.

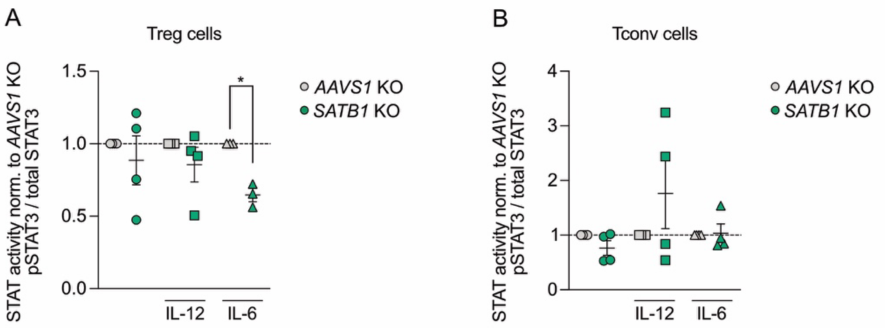

Figure Letter 6: STAT3 activity in SATB1 KO Treg and Tconv cells. pSTAT3(Tyr705) and STAT3 levels were detected in AAVS1 and SATB1 KO Treg and Tconv cells by western blot analysis and normalized to γ-Tubulin. STAT3 activity was calculated as pSTAT3/STAT3 and normalized to the respective AAVS1 KO controls. n = 4, biological replicates.

Minor Comments:
It would be helpful for the reader if the authors explicitly described how SATB1 knockout efficiency was assessed in the Results section.

The methodologies to determine the KO efficiencies for SATB1 and AAVS1 control KOs are described in the sections “Amplicon Sanger Sequencing” and “Amplicon NGS”. For clarification reasons we changed the titles to “Quantification of KO efficiencies with Amplicon Sanger Sequencing” and “Quantification of KO efficiencies with Amplicon NGS” of these paragraphs and revised the language in these Material and Methods sections.

Can the authors confirm whether the heatmap displaying fold changes of pro- and anti-inflammatory flow cytometry markers in SATB1 KO Tregs and SATB1 KO Tconv was normalized to AAVS1 KO control Tregs and Tconv, respectively?

Yes, the data displayed in Figure 1B were normalized to the respective *AAVS1* KO control cells. This information is added to the figure legend and is depicted within Figure 1B.

Were Tregs cultured with IL-2 between days 12 and 15?

Yes, all KO Tregs cells were cultured with 600 U/ml IL-2 (saturation) between days 12 and 15. We clarified this important point in the results section and in the figure legends. Details can be found in the Material and Methods section.

In Figure 1C, the subpopulation of Tregs producing IL-2 represents only a minority of the opt-SNE contour plot and appears FOXP3-negative. Could this reflect contamination by effector T cells?

We shared the concern of the reviewer about potential contamination of the Treg cultures with Tconv. Treg cells were sorted in a purity of minimum 98% and cultured without recombinant IL-2 for the first 48 hrs to minimize the risk of the expansion of potential contaminant Tconv cells. On day 7 of Treg cell culture, we conducted a quality control and only continue with cultures that contain over 95% FOXP3+ CTLA-4+ double positive Treg cells. A representative example of expanded Treg cells at day7 is shown in Figure Letter 7. We mention now this quality control step in the Material and Method section.
Furthermore, the color scale applied in Figure 1C or Figure EV2A, C, FOXP3-negative cells are displayed in dark blue. We see a small proportions of Treg cells which are FOXP3-negative in Figure 1C (top right). This cell population does not correlate with proinflammatory cytokine production. IL-2- and IFNγ-producing Treg cells still show high to medium FOXP3 levels (Figure 1C, bottom). IL-2 secreting FOXP3+ memory Treg cells have been reported previously (Benfeldt et al., 2012 Front. Immunol.).

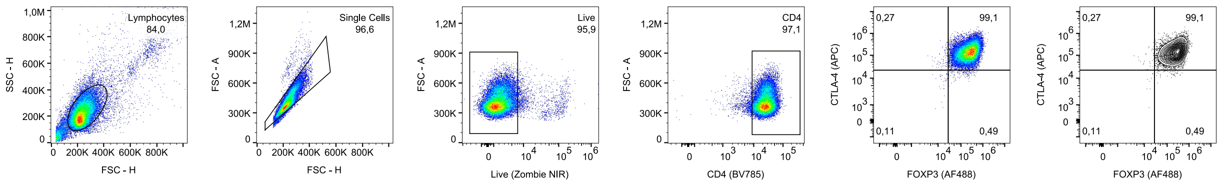

Figure Letter 7: Representative examples of ex vivo expanded human Treg cells at day 7 of culture. Experiments were only conducted with Treg cell products with a frequency of FOXP3+CTLA-4+ double positive Treg cells > 95%.

In Figure 1E, given the variability in the data, should the results be paired by donor?

We agree with the reviewer and changed the data presentation in Figure 1E.

It would be helpful to clearly state in the Results section that PMA/ionomycin stimulation was used, as this could represent a confounding factor.

We highlight now in the revised manuscript the stimulation conditions for the cytokine stainings in the results section.

The statement, "Overall, SATB1 ablation in Treg and Tconv cells resulted in subset-specific regulation of pro-inflammatory cytokines and a general boost in IL-10 production," appears to be an overinterpretation, as only a very small subset of cells produces IL-10 (based on Figure 1B).

We agree with the reviewer that this sentence needs rephrasing. We wanted to highlight that we see an increase in IL-10 protein levels in supernatants of *SATB1* KO cells. We now also added additional biological replicates to show this effect more clearly. We rephrased this sentence to make clear that we see a consistent difference in IL-10 protein levels, but that these changes derive from an IL-10 producing subpopulation of *SATB1* KO Treg and Tconv cells.

Typographical correction needed: "SATB1 KO Tconv cellschanges" → add a space between "cells" and "changes."

We corrected this error.

A more detailed discussion of the opposing results observed between murine and human SATB1 KO Treg cells would strengthen the manuscript.

We agree with the reviewer that this is a very important point and discuss now differences in Treg cell function and phenotypes between the different species in more detail in the discussion section of the manuscript.

A limitations paragraph should be included in the Discussion section. For example: CD8 T cells were not analyzed (particularly in vivo), which limits conclusions regarding the suitability of targeting SATB1 in multiple T cell subsets for immunotherapy. Differentiation and exhaustion profiles of CAR-T cells were not assessed in vivo. Flow cytometry, ATAC-seq, and RNA-seq analyses were performed exclusively after PMA/ionomycin stimulation, with no comparison to other conditions (e.g., no stimulation, anti-CD3/CD28 bead stimulation, or CAR-specific stimulation).

We would like to thank the reviewer for this helpful suggestion. We now added a section in the discussion part pointing out the limitations of our study and the future perspectives.

Referee #3:

This study provides a thorough and well-executed analysis of SATB1 function in human CD4

T cell subsets, with a particular focus on Treg and CD4 CAR T cells. The authors convincingly demonstrate that SATB1 acts as a key regulator of subset-specific gene programs, controlling cytokine expression and JAK-STAT signaling in a cell-type-specific manner. Functionally, SATB1 ablation weakens Treg suppressive activity while enhancing CD4 CAR T cell-mediated tumor clearance in a humanized mouse model, highlighting the translational relevance of modulating SATB1 to improve CAR T therapy. The work is strengthened by a multi-layered dataset, including chromatin, gene expression, and functional analyses, which together robustly support the conclusions.

We are grateful for the reviewer`s positive comments and for evaluating our study as “thorough and well-executed”.

With modest revisions, the manuscript could be even more accessible and precise. Specifically, grammatical errors (e.g., "Despite of the distinct gene-regulatory patterns") and typographical issues (e.g., "cellschanges," spacing inconsistencies, truncated labels) should be corrected. Quantitative descriptions should accompany terms like "slight" to improve clarity, and terminology should be consistent across figures and legends (e.g., "Tconv" vs. "Teff"). Finally, statistical annotations would benefit from briefly restating the tests used in figure legends when multiple tests are presented. Overall, this is a strong and impactful study with clear mechanistic insights and translational relevance.

We thank the reviewer for his/her critical evaluation of our manuscript. In the revised version of our manuscript, we corrected the grammatical and typographical errors. We also added more quantitative descriptions to improve the clarity of the manuscript (highlighted in the text). Based on the reviewer´s feedback we added more information (e.g. biological replicates), to the figure legends.

Dear Prof. Schumann,

Thank you for the submission of your revised manuscript to our editorial offices. I have now received the reports from the two referees that were asked to re-evaluate the study, you will find below. As you will see, both referees now fully support the publication of the study in EMBO reports.

Before we can proceed with formal acceptance, I have the editorial requests below I ask you to address in a final revised manuscript. Please also provide a final p-b-p-response to the editorial requests.

Editorial requests:

- Please order the manuscript sections like this, using only these names:
Title page - Abstract - Keywords - Introduction - Results - Discussion - Methods - Data availability section - Acknowledgements - Disclosure and Competing Interests Statement - References - Figure legends - Expanded View Figure legends

- Please check again that the number "n" for how many independent experiments were performed, their nature (biological versus technical replicates), the bars and error bars (e.g. SEM, SD) and the test used to calculate p-values is indicated in the respective figure legends (main and EV figures). Please also check that all the p-values are explained in the legend, and that these fit to those shown in the figure. Please provide statistical testing where applicable. Please avoid the phrase 'independent experiment' but clearly state if these were biological or technical replicates. Please also indicate (e.g. with n.s.) if testing was performed, but the differences are not significant. In case n=2, please show the data as separate datapoints without error bars and statistics. See also:

https://link.springer.com/journal/44319/submission-guidelines#cms-Figure-and-data-presentation

If n<5, please show single datapoints for diagrams. Presently, some diagrams show incomplete statistics or miss the 'n.s.'. Please provide proper and complete statistical testing for all diagrams shown (except when n=2). Moreover:
- Please note that the exact p values are not provided in the legends of figures 1E-H; 5C, D; 6F, H; EV1 E, F; EV3 A, B, D, E, F
- Please indicate the statistical test used for data analysis in the legends of figures 3A-D; 4A, B

- Please remove now the referee access from the data availability section and add direct links to each dataset. Please make sure the datasets are public latest upon online publication of the paper.

- Please make sure that all the funding information is also entered into the online submission system and that it is complete and similar to the one in the acknowledgement section of the manuscript text file. Presently, the grant 'Project Nr. 659389' is not listed in the submission system. Please check.

- Please add the primer/sequence information provided in Dataset EV1 to the Reagents and Tools table and update any callouts. Please also remove the instructions text from the reagents and tools table. Finally, please remove Dataset EV1 from the submission, rename present Dataset EV2 into Dataset EV1 and update the callouts.

- Please provide a legend for present Dataset EV2 (to be re-named into Dataset EV1) on the first TAB of the excel file.

- Thanks for providing the source data. Please upload the source data as one folder per main figure, grouping together the separate files for the panels (clearly labelled), and ZIPed together.

In addition, I would need from you uploaded separately:
- a short, two-sentence summary of the manuscript (not more than 35 words).
- two to four short (!) bullet points highlighting the key findings of your study (two lines each).
- a schematic summary figure as separate file that provides a sketch of the major findings (not a data image) in jpeg or tiff format (with the exact width of 550 pixels and the exact height of 300 pixels) that can be used as a visual synopsis on our website.

I look forward to seeing the further revised version of your manuscript when it is ready. Please let me know if you have questions regarding the revision.

Achim Breiling
Senior Editor
EMBO Reports

Referee #1:

The authors have adequately addressed mine and the other reviewers' comments. I recommend for publication without reservation.

Referee #2:

The authors have satisfactorily addressed all comments.

The authors have addressed all minor editorial requests.

Prof. Kathrin Schumann
University of Konstanz
Biology
Universitätsstrasse 10
Konstanz 78457
Germany

Dear Prof. Schumann,

I am very pleased to accept your manuscript for publication in the next available issue of EMBO reports. Thank you for your contribution to our journal.

Your manuscript will be processed for publication by EMBO Press. It will be copy edited and you will receive page proofs prior to publication. Please note that you will be contacted by Springer Nature Author Services to complete licensing and payment information.

You may qualify for financial assistance for your publication charges - either via a Springer Nature fully open access agreement or an EMBO initiative. Check your eligibility: https://link.springer.com/journal/44319/how-to-publish-with-us

Should you be planning a Press Release on your article, please get in contact with embo_production@springernature.com as early as possible in order to coordinate publication and release dates.

If you have any questions, please do not hesitate to contact the Editorial Office. Thank you for your contribution to EMBO Reports.

Yours sincerely,

Achim Breiling
Senior Editor
EMBO Reports

>>> Please note that it is EMBO Reports policy for the transcript of the editorial process (containing referee reports and your response letter) to be published as an online supplement to each paper. If you do NOT want this, you will need to inform the Editorial Office via email immediately. More information is available here: https://link.springer.com/partners/embo-press/editorial-policies#Peer%20review

EMBO Press Author Checklist

Corresponding Author Name: Kathrin Schumann
Journal Submitted to: EMBO Reports
Manuscript Number: EMBOR-2025-63316V2

USEFUL LINKS FOR COMPLETING THIS FORM
The EMBO Journal - Author Guidelines
EMBO Reports - Author Guidelines
Molecular Systems Biology - Author Guidelines
EMBO Molecular Medicine - Author Guidelines

Reporting Checklist for Life Science Articles (updated January
This checklist is adapted from Materials Design Analysis Reporting (MDAR) Checklist for Authors. MDAR establishes a minimum set of requirements in transparent reporting in the life sciences (see Statement of Task: 10.31222/osf.io/9sm4x). Please follow the journal's guidelines in preparing your
Please note that a copy of this checklist will be published alongside your article.

Abridged guidelines for figures
1. Data
The data shown in figures should satisfy the following conditions:
- ➞ the data were obtained and processed according to the field's best practice and are presented to reflect the results of the experiments in an accurate and unbiased manner.
- ➞ ideally, figure panels should include only measurements that are directly comparable to each other and obtained with the same assay.
- ➞ plots include clearly labeled error bars for independent experiments and sample sizes. Unless justified, error bars should not be shown for technical
- ➞ if n<5, the individual data points from each experiment should be plotted. Any statistical test employed should be justified.
- ➞ Source Data should be included to report the data underlying figures according to the guidelines set out in the authorship guidelines on Data

2. Captions
Each figure caption should contain the following information, for each panel where they are relevant:
- ➞ a specification of the experimental system investigated (eg cell line, species name).
- ➞ the assay(s) and method(s) used to carry out the reported observations and measurements.
- ➞ an explicit mention of the biological and chemical entity(ies) that are being measured.
- ➞ an explicit mention of the biological and chemical entity(ies) that are altered/varied/perturbed in a controlled manner.
- ➞ the exact sample size (n) for each experimental group/condition, given as a number, not a range;
- ➞ a description of the sample collection allowing the reader to understand whether the samples represent technical or biological replicates (including how many animals, litters, cultures, etc.).
- ➞ a statement of how many times the experiment shown was independently replicated in the laboratory.
- ➞ definitions of statistical methods and measures:
  - common tests, such as t-test (please specify whether paired vs. unpaired), simple χ2 tests, Wilcoxon and Mann-Whitney tests, can be unambiguously identified by name only, but more complex techniques should be described in the methods section;
  - are tests one-sided or two-sided?
  - are there adjustments for multiple comparisons?
  - exact statistical test results, e.g., P values = x but not P values < x;
  - definition of 'center values' as median or average;
  - definition of error bars as s.d. or s.e.m.

Please complete ALL of the questions below.
Select "Not Applicable" only when the requested information is not relevant for your study.

Materials

Newly Created Materials	Information included in the manuscript?	In which section is the information available? (Reagents and Tools Table, Materials and Methods, Figures, Data Availability Section)
New materials and reagents need to be available; do any restrictions apply?	Not Applicable	

Antibodies	Information included in the manuscript?	In which section is the information available? (Reagents and Tools Table, Materials and Methods, Figures, Data Availability Section)
For **antibodies** provide the following information:<br>- Commercial antibodies: RRID (if possible) or supplier name, catalogue number and or/clone number<br>- Non-commercial: RRID or citation	Yes	Reagents an Tools Table, Material and Methods

DNA and RNA sequences	Information included in the manuscript?	In which section is the information available? (Reagents and Tools Table, Materials and Methods, Figures, Data Availability Section)
**Short novel DNA or RNA including primers, probes**: provide the sequences.	Yes	Dataset EV1

Cell materials	Information included in the manuscript?	In which section is the information available? (Reagents and Tools Table, Materials and Methods, Figures, Data Availability Section)
**Cell lines:** Provide species information, strain. Provide accession number in repository **OR** supplier name, catalog number, clone number, and/**OR** RRID.	Yes	Reagents an Tools Table, Material and Methods
**Primary cultures:** Provide species, strain, sex of origin, genetic modification status.	Yes	Material and Methods (human T cells, genetic modifications/CRISPR editing)
Report if the cell lines were recently **authenticated** (e.g., by STR profiling) and tested for mycoplasma contamination.	Yes	Material and Methods (mycoplasma testing; no STR profiling)

Experimental animals	Information included in the manuscript?	In which section is the information available? (Reagents and Tools Table, Materials and Methods, Figures, Data Availability Section)
**Laboratory animals or Model organisms:** Provide species, strain, sex, age, genetic modification status. Provide accession number in repository **OR** supplier name, catalog number, clone number, **OR** RRID.	Yes	Reagents an Tools Table, Material and Methods
**Animal observed in or captured from the field:** Provide species, sex, and age where possible.	Not Applicable	
Please detail **housing and husbandry conditions**.	Yes	Material and Methods

Plants and microbes	Information included in the manuscript?	In which section is the information available? (Reagents and Tools Table, Materials and Methods, Figures, Data Availability Section)
**Plants:** provide species and strain, ecotype and cultivar where relevant, unique accession number if available, and source (including location for collected wild specimens).	Not Applicable	
**Microbes:** provide species and strain, unique accession number if available, and source.	Not Applicable	

Human research participants	Information included in the manuscript?	In which section is the information available? (Reagents and Tools Table, Materials and Methods, Figures, Data Availability Section)
If collected and within the bounds of privacy constraints report on age, sex and gender or ethnicity for all study participants.	Not Applicable	

Core facilities	Information included in the manuscript?	In which section is the information available? (Reagents and Tools Table, Materials and Methods, Figures, Data Availability Section)
If your work benefited from core facilities, was their service mentioned in the acknowledgments section?	Yes	Acknowledgments

Design

Study protocol	Information included in the manuscript?	In which section is the information available? (Reagents and Tools Table, Materials and Methods, Figures, Data Availability Section)
If study protocol has been **pre-registered, provide DOI in the manuscript**. For clinical trials, provide the trial registration number **OR** cite DOI.	Not Applicable	
Report the **clinical trial registration number** (at ClinicalTrials.gov or equivalent), where applicable.	Not Applicable	

Laboratory protocol	Information included in the manuscript?	In which section is the information available? (Reagents and Tools Table, Materials and Methods, Figures, Data Availability Section)
Provide DOI OR other citation details if **external detailed step-by-step protocols** are available.	Not Applicable	comment: standard methodologies

Experimental study design and statistics	Information included in the manuscript?	In which section is the information available? (Reagents and Tools Table, Materials and Methods, Figures, Data Availability Section)
Include a statement about **sample size** estimate even if no statistical methods were used.	Yes	Material and Methods
Were any steps taken to minimize the effects of subjective bias when allocating animals/samples to treatment (e.g. **randomization procedure**)? If yes, have they been described?	Not Applicable	
Include a statement about **blinding** even if no blinding was done.	Yes	Material and Methods
Describe **inclusion/exclusion criteria** if samples or animals were excluded from the analysis. Were the criteria pre-established?<br><br>If sample or data points were omitted from analysis, report if this was due to attrition or intentional exclusion and provide justification.	Yes	Material and Methods
For every figure, are **statistical tests** justified as appropriate? Do the data meet the assumptions of the tests (e.g., normal distribution)? Describe any methods used to assess it. Is there an estimate of variation within each group of data? Is the variance similar between the groups that are being statistically compared?	Yes	Figure legends

Sample definition and in-laboratory replication	Information included in the manuscript?	In which section is the information available? (Reagents and Tools Table, Materials and Methods, Figures, Data Availability Section)
In the figure legends: state number of times the experiment was **replicated** in laboratory.	Yes	biological, technical replicates are indicated
In the figure legends: define whether data describe **technical or biological replicates**.	Yes	biological, technical replicates are indicated

Ethics

Ethics	Information included in the manuscript?	In which section is the information available? (Reagents and Tools Table, Materials and Methods, Figures, Data Availability Section)
Studies involving **human participants**: State details of **authority granting ethics approval** (IRB or equivalent committee(s), provide reference number for approval.	Yes	Material and Methods
Studies involving **human participants**: Include a statement confirming that **informed consent** was obtained from all subjects and that the experiments conformed to the principles set out in the WMA Declaration of Helsinki and the Department of Health and Human Services Belmont Report.	Yes	Material and Methods
Studies involving **human participants:** For publication of **patient photos**, include a statement confirming that consent to publish was obtained.	Not Applicable	
Studies involving experimental **animals**: State details of **authority granting ethics approval** (IRB or equivalent committee(s), provide reference number for approval. Include a statement of compliance with ethical regulations.	Yes	Material and Methods
Studies involving **specimen and field samples:** State if relevant **permits** obtained, provide details of authority approving study; if none were required, explain why.	Not Applicable	

Dual Use Research of Concern (DURC)	Information included in the manuscript?	In which section is the information available? (Reagents and Tools Table, Materials and Methods, Figures, Data Availability Section)
Could your study fall under dual use research restrictions? Please check biosecurity documents and list of **select agents and toxins** (CDC): https://www.selectagents.gov/sat/list.htm	Not Applicable	
If you used a select agent, is the security level of the lab appropriate and reported in the manuscript?	Not Applicable	
If a study is subject to dual use research of concern regulations, is the name of the **authority granting approval and reference number** for the regulatory approval provided in the manuscript?	Not Applicable	

Reporting

The MDAR framework recommends adoption of discipline-specific guidelines, established and endorsed through community initiatives. Journals have their own policy about requiring specific guidelines and recommendations to complement MDAR.

Adherence to community standards	Information included in the manuscript?	In which section is the information available? (Reagents and Tools Table, Materials and Methods, Figures, Data Availability Section)
State if relevant guidelines or checklists (e.g., **ICMJE, MIBBI, ARRIVE, PRISMA**) have been followed or provided.	Not Applicable	
For **tumor marker prognostic studies,** we recommend that you follow the **REMARK** reporting guidelines (see link list at top right). See author guidelines, under 'Reporting Guidelines'. Please confirm you have followed these guidelines.	Not Applicable	
For **phase II and III randomized controlled trials**, please refer to the **CONSORT** flow diagram (see link list at top right) and submit the CONSORT checklist (see link list at top right) with your submission. See author guidelines, under 'Reporting Guidelines'. Please confirm you have submitted this list.	Not Applicable	

Data Availability

Data availability	Information included in the manuscript?	In which section is the information available? (Reagents and Tools Table, Materials and Methods, Figures, Data Availability Section)
Have **primary datasets** been deposited according to the journal's guidelines (see 'Data Deposition' section) and the respective accession numbers provided in the Data Availability Section?	Yes	Data accessibility section
Were **human clinical and genomic datasets** deposited in a public access-controlled repository in accordance to ethical obligations to the patients and to the applicable consent agreement?	Yes	Data accessibility section
Are **computational models** that are central and integral to a study available without restrictions in a machine-readable form? Were the relevant accession numbers or links provided?	Yes	Material and Methods
If publicly available data were reused, provide the respective **data citations in the reference list**.	Not Applicable	
